# Multi-Component One-Pot Reaction of Aromatic Carbonyl Compounds, Tosylhydrazide, and Arylboronic Acids

**DOI:** 10.3390/molecules22122168

**Published:** 2017-12-07

**Authors:** Ningning Gu, Yu Wei, Ping Liu, Yan Liu, Bin Dai

**Affiliations:** Key Laboratory for Green Processing of Chemical Engineering of Xinjiang Bingtuan, School of Chemistry and Chemical Engineering, Shihezi University, Shihezi 832003, China; tenggeli116@126.com (N.G.); yuweichem@126.com (Y.W.); db_tea@shzu.edu.cn (B.D.)

**Keywords:** one-pot reaction, tosylhydrazide, arylboronic acid, 4-benzyl-1,1′-biphenyl derivatives

## Abstract

In this paper, we developed a new method using 4-bromoacetophenone as the starting material, with tosylhydrazide and two arylboronic acids using Barluenga and Suzuki couplings in a four-component one-pot reaction to afford the target product 4-benzyl-1,1′-biphenyls. This system that we have developed enables the use of easily accessible starting materials and can be employed on a wide variety of substrates with good functional group tolerance. In particular, this protocol can be applied to the synthesis of 4-(1-([1,1′-biphenyl]-4-yl)ethyl)pyridine derivatives, a class of potential analogs of CPY17 inhibitors of prostate cancer.

## 1. Introduction

**Scheme 1:**
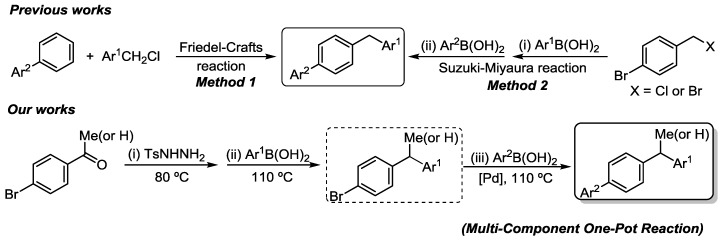
Synthetic methods for 4-benzyl-1,1′-biphenyl derivatives.

4-Benzyl-1,1′-biphenyl derivatives are important structural units in numerous thermal recording materials, electrophotographic photoreceptors and biologically active molecules, including a number of anticancer agents [1,2,3,4,5,6,7,8,9,10,11]. Typically, the Friedel-Crafts reaction (Scheme 1, Method 1) is employed for the synthesis of such compounds, which may be catalyzed by solid acid catalysts [12,13,14]. Particularly, patents have been reported on the synthesis of benzyl-1,1′-biphenyl derivatives starting from biphenyl and benzyl chloride using catalysts such as montmorillonite and zeolites [15,16]. Previously, the kinetics of the reaction between biphenyl and benzyl chloride over dealuminated HY zeolites has been reported [17]. In addition, the Suzuki–Miyaura reaction, which involves the coupling of an arylboronic acid derivative and an organohalide, proved to be an extremely useful synthetic tool for the construction of a biphenyl scaffold. Among others, some convenient features involved in the use of arylboronic acid precursors in these reactions are as follows: arylboronic acid derivatives demonstrate air and moisture stability, relatively low toxicity and high compatibility to various functional groups [18,19,20,21]. However, the synthesis of 4-benzyl-1,1′-biphenyl derivatives by the Suzuki-Miyaura reaction is more challenging (Scheme 1, Method 2). More often, this protocol suffers from poor selectivity in the first step, resulting in a mixture of mono- and bis-coupled products, thereby necessitating the purification of the intermediate. Chemoselectivity is crucial if sequential cross-coupling reactions are to be effective. It may be achieved using precursors containing activating groups of different reactivities, such as 1-bromo-4-(halomethyl)benzene [22,23]. Multicomponent reactions (MCRs) are ideal synthetic tools for generating complex molecules from readily available starting materials in a single synthetic operation; hence, they minimize waste generation, rendering green transformations [24,25,26,27]. The separation and purification of this step raises the cost and decrease the efficiency of this synthesis method. Therefore, it is desirable to develop a more efficient synthetic path.

Recently, *N*-tosylhydrazones as important intermediates have attracted extensive attention because of their various useful applications in organic synthesis [28,29,30,31,32,33,34,35]. Significant progress has been achieved in sequential transformations of *N*-tosylhydrazones through auto tandem catalysis [36,37,38]. In 2010, Wang et al. developed MCRs of *N*-tosylhydrazones with an arylhalide and a terminal alkyne [39]. In 2013, Hamze et al. reported a three-component one-pot reaction of hydrazones, dihaloarenes and amines [40,41]. On the basis of our previous work [42,43,44,45], we herein report a new multi-component reaction of aromatic carbonyl compounds, tosylhydrazide and aryl boronic acids for the synthesis of 4-benzyl-1,1′-biphenyl derivatives (Scheme 1).

## 2. Results and Discussion

**Figure 1:**
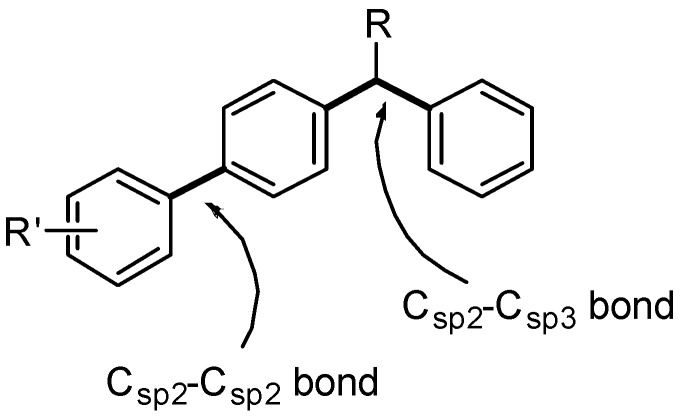
Structure of 4-benzyl-1,1′-biphenyl derivatives.

First, from the structural analysis of the 4-(1-phenylethyl)-1,1′-biphenyl skeleton (Figure 1), Csp^2^–Csp^2^ bond and Csp^2^–Csp^3^ bonds were constructed for building such a molecular structure. The Csp^2^–Csp^3^ bond is well known to be obtained by the reductive coupling reaction of aryl hydrazone with an aryl boronic acid, and the Csp^2^–Csp^2^ bond is formed by the palladium-catalyzed Suzuki coupling of aryl halide with an arylboronic acid. One common factor between these two reactions is the use of a base, but the Suzuki coupling reaction usually requires the involvement of a palladium catalyst. Hence, if we use the 4′-halo-acetophenone as starting reactants, and optimize the reaction conditions, it is possible to easily achieve access to 4-benzyl-1,1′-biphenyl derivatives by a one-pot multi-step reaction. It is an ideal route to synthesis of the target compound by a simple and efficient way.

**Scheme 2:**
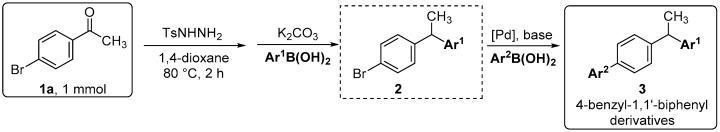
One-pot, multi-step, multi-component reactions of 4-bromoacetophenone, tosylhydrazide, with two arylboronic acids.

Based on the above considerations, we decided to use cost-effective, readily available 4-bromoacetophenone as the starting material. *N*-tosylhydrazones were generated in situ using TsNHNH_2_, reacted with an arylboronic acid to afford a 4-bromo-diarylmethane intermediate by reductive coupling, leading to the formation of a Csp^2^–Csp^3^ bond. With treatment using a transition-metal palladium catalyst, without isolation, the intermediate product was treated with another arylboronic acid by Suzuki coupling to construct the Csp^2^–Csp^2^ bond, ultimately affording 4-benzyl-1,1′-biphenyl derivatives (Scheme 2).

On the basis of their study and our previous work [17], the reaction conditions of the third step were optimized, namely, the effects of ligand, base and temperature on the Suzuki coupling reaction were examined. Initially, the coupling reaction of 4-bromoacetophenone, TsNHNH_2_ and phenylboronic acid was selected as a model reaction to optimize the reaction conditions. Table 1 summarizes the results. The use of only Pd(OAc)_2_ as the catalyst and K_2_CO_3_ as the base in 1,4-dioxane at 110 °C produced the desired product **3a** in only 15% yield (entry 1). The phosphine ligands were screened for this reaction, and PCy_3_·HBF_4_ was found to be the most effective one for forming coupling product **3a** (entry 3). Among the various bases examined, organic bases (Et_3_N), and inorganic bases (K_3_PO_4_·H_2_O and NaOH) gave the desired product in moderate yields (63–72%, entries 4–6, respectively). Cs_2_CO_3_ was not effective, and corresponding product **3a** was obtained in a low yield (entry 7). Gratifyingly, when the amounts of tosylhydrazide and phenylboronic acid were increased, the yield of the product dramatically increased to 89% (entry 8). In addition, when the amount of Pd(OAc)_2_ was increased to 2 mol %, the yield of product did not improve significantly (entry 9). When the temperature of the Suzuki coupling reaction was decreased to 80 °C, the product was isolated in only 52% yield (entry 10). Hence, the combination of 4-bromoacetophenone (0.5 mmol), tosylhydrazide (1.5 equiv.), phenylboronic acid (3.0 equiv.), Pd(OAc)_2_ (1 mol %), PCy_3_·HBF_4_ (4 mol %) and K_2_CO_3_ (3.0 equiv.) at T_1_ = 80 °C (t_1_ = 2 h), T_2_ = 110 °C (t_2_ = 5 h), and T_3_ = 110 °C (t_3_ = 12 h) in 1,4-dioxane (5 mL) were found to be the most suitable reaction conditions.

**Figure 2:**
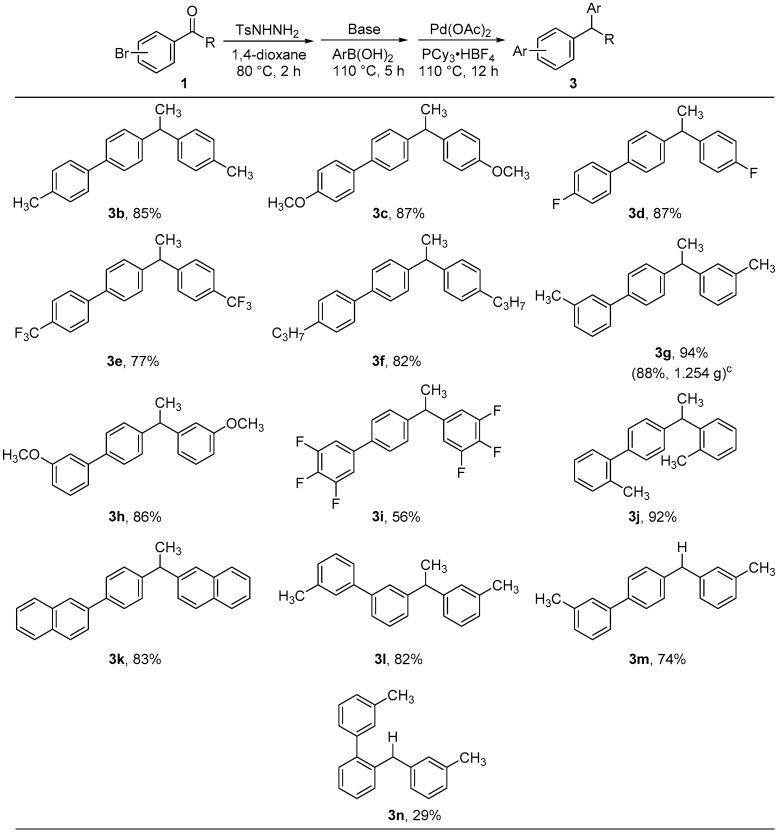
One-pot, four-component reactions of 4-bromophenyl aldehydes or ketones, TsNHNH_2_, with two same arylboronic acids ^a,b^. ^a^ Reaction conditions: (i) 0.5 mmol carbonyl compound, 0.75 mmol tosylhydrazide, 4 mL 1,4-dioxane, 80 °C, 2 h; (ii) 1.5 mmol boronic acid, 1.5 equivalent K_2_CO_3_, 110 °C, 5 h. (iii) 1 mol % Pd(OAc)_2_, 4 mol % PCy_3_∙HBF_4_, 110 °C, 12 h. ^b^ Isolated yield. ^c^ Reaction conditions: (i) 5 mmol 4-bromoacetophenone, 7.5 mmol tosylhydrazide, 20 mL dioxane, 80 °C, 16 h; (ii) 3.0 equivalent *m*-tolylboronic acid, 1.5 equivalent K_2_CO_3_, 110 °C, 10 h; (iii) 1 mol % Pd(OAc)_2_, 4 mol % PCy_3_∙HBF_4_, 110 °C, 22 h.

With the optimized conditions, we further investigated the substrate scope of this one-pot, three-step reductive coupling reaction process. As shown in Figure 2, the 4-substituted arylboronic acid substrates bearing electron-withdrawing or electron-donating groups effectively afforded desired products **3b**–**3f** in good yields (77–87%). Moreover, *m*-tolylboronic acid, (3-methoxyphenyl)boronic acid and (3,4,5-trifluorophenyl)boronic acid were found to produce desired coupling products **3g**–**3i** in 56–94% yields, respectively. Furthermore, the reductive coupling reactions involving ortho-substituted arylboronic acids were investigated. Both electronic properties and steric hindrance of the substrates affect the coupling reaction. For example, the coupling reaction of *o*-tolylboronic acid with 4-bromoacetophenone and TsNHNH_2_ afforded **3j** in 92% yield. Naphthalene-2-ylboronic acid used as the coupling partner afforded **3k** in 83% yield. Moreover, 3-bromoacetophenone and 4-bromobenzaldehyde also reacted with *m*-tolylboronic acid affording target products **3l** and **3m** in 82% and 74% yields. In contrast, 2-bromobenzaldehyde as the substrate reacted with *m*-tolylboronic acid affording the desired product **3n** in only 29% yield. Notably, the gram-scale synthesis of **3g** was performed to verify the practical application of this synthesis system. Fortunately, the reaction was performed using 5 mmol of 4-bromoacetophenone, 7.5 mmol TsNHNH_2_ and 15 mmol *m*-tolylboronic acid, affording product **3g** in 88% yield (1.254 g).

**Figure 3:**
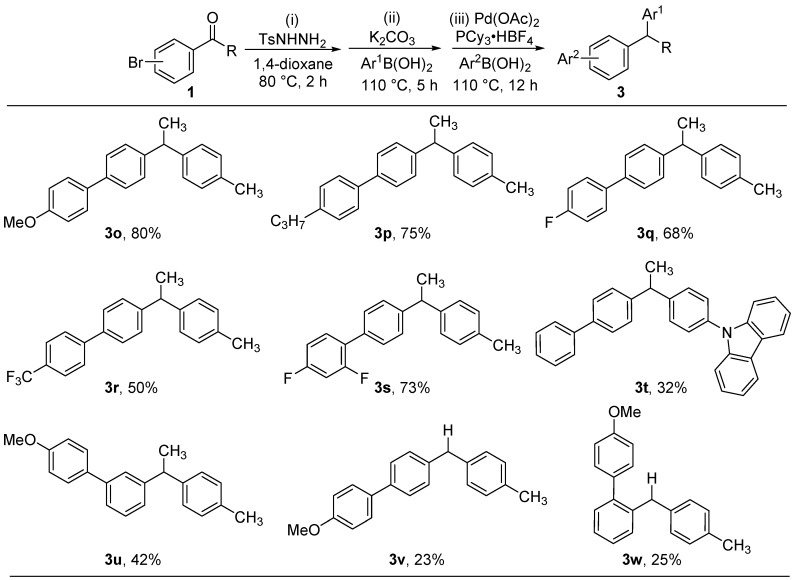
One-pot, four-component reactions of 4-bromophenyl aldehydes or ketones, TsNHNH_2_, with two different arylboronic acids ^a,b^. ^a^ Reaction conditions: (i) 0.5 mmol carbonyl compound, 0.5 mmol tosylhydrazide, 4 mL 1,4-dioxane, 80 °C, 2 h; (ii) 0.55 mmol Ar^1^B(OH)_2_, 1.5 equivalents K_2_CO_3_, 110 °C, 5 h. (iii) 1 mol % Pd(OAc)_2_, 4 mol % PCy_3_∙HBF_4_, 0.75 mmol Ar^2^B(OH)_2_, 110 °C, 12 h. ^b^ Isolated yield.

For further ascertaining the applicable scope of this methodology, various arylboronic acids and carbonyl compounds were examined. As shown in Figure 3, arylboronic acids bearing 4-methoxy, 4-*n*-propyl, 4-fluoro, 4-trifluoromethyl and 2,4-difluoro groups were transformed into corresponding products **3o**–**3s** in 50–80% yields. The substrate containing carbazolyl groups only afforded products **3t** in 32% yield. In addition to 4-bromoacetophenone, 1-(3-bromophenyl)ethan-1-one and 4-bromobenzaldehyde or 2-bromobenzaldehyde also could be applied, affording products **3u**–**3w** in 23–42% yields.

**Scheme 3:**
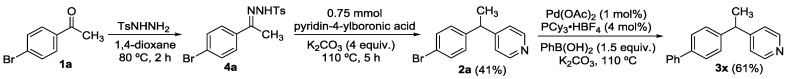
Synthesis of **3x** by three-step, four-component reactions.

Next, we intend to apply this one-pot, multi-step reactions for preparing compound **3x**, an analog of CPY17 inhibitors [10]. However, the reaction did not proceed smoothly in one-pot. Hence, we decided to adopt a step-wise approach for synthesizing **3x**. First, 4-bromoacetophenone reacted with TsNHNH_2_ to afford the acylhydrazone. Then, it reduced coupling with 4-pyridine boronic acid afforded the intermediate product 4-(1-(4-bromophenyl)ethyl)pyridine with 41% isolated yield. Finally, the 4-(1-(4-bromophenyl)ethyl)pyridine was treated with phenylboronic acid, affording coupling product **3x** in 61% yield, but the total yield was only 24.6% (Scheme 3).

**Scheme 4:**
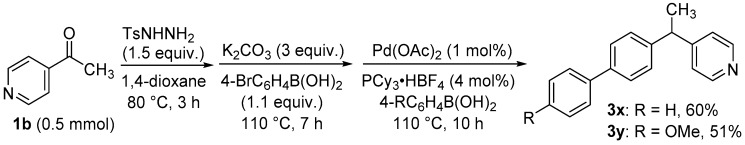
Synthesis of **3x** and **3y** by one-pot, three-step, four-component reactions.

In response to the challenges above, we also attempted to synthesize **3x** by an alternative route (Scheme 4). Using 4-acetylpyridine as the starting material, first reacted with TsNHNH_2_, generates the corresponding hydrazine, then, to reductive coupling with 4-bromophenylboronic acid. In the third step, the second phenylboronic acid with a palladium catalyst were added to the above reaction solution, after the Suzuki coupling reaction, the ultimate product **3x** formed with 60% yield directly without the need to isolate any of the reaction intermediates. Similarly, methoxy substituted product **3y** was also obtained with 51% yield by this method.

## 3. Materials and Methods

Chemicals were obtained commercially and used as received. NMR spectra were recorded on a Bruker DPX-400 spectrometer using TMS as the internal standard. EI-Mass spectra were measured on a LC/Q-TOF MS (Micromass, Wilmslow, UK) (see Supplementary Materials). All products were isolated by short chromatography on a silica gel (200–300 mesh) column using petroleumether (60–90 °C) as mobile phase, unless otherwise noted. 4-Bromoacetophenone derivatives, and arylboronic acids were of analytical grade quality, purchased from Adamas-beta Pharmaceuticals, Inc. (Shanghai, China).

### 3.1. General Procedure for the One-Pot, Four-Component Reactions of 4-Bromoacetophenone, TsNHNH_2_, and Two Same Arylboronic Acids

A solution of 4-bromoacetophenone or 4-bromobenzaldehyde derivatives (0.5 mmol) and tosylhydrazide (0.75 mmol) in 4 mL of 1,4-dioxane was stirred at 80 °C for 2 h in a reaction tube. Second, potassium carbonate (1.5 mmol) and the appropriate arylboronic acid (1.1 mmol) were added to the reaction mixture. Third, the reaction system was refluxed at 110 °C for 5 h with stirring. Next, 2 mol % Pd(OAc)_2_, and 4mol % PCy_3_·HBF_4_ were added, and the reaction was continued for 12 h at 110 °C. Then, the reaction was completed, and the crude mixture was allowed to reach room temperature. Finally, dichloromethane and a saturated solution of NaHCO_3_ were added, and the layers were separated. The aqueous phase was extracted three times with dichloromethane, and the combined organic layers were washed with two portions of a saturated NaHCO_3_ solution and one portion of brine, and then dried over anhydrous MgSO_4_ and filtered. The solvent was removed under reduced pressure. The products were purified by silica gel chromatography.

### 3.2. General Procedure for the One-Pot, Four-Step, Four-Component Reactions of 4-Acetylbiphenylderivatives, TsNHNH_2_, and Two Different Arylboronic Acids

A solution of 4-bromoacetophenone derivatives (0.5 mmol) and tosylhydrazide (0.75 mmol) in 4 mL of 1,4-dioxane was stirred at 80 °C for 2 h in a reaction tube. Then, potassium carbonate (1.5 mmol) and the appropriate arylboronic acid (0.6 mmol) were added to the reaction mixture. The system was refluxed at 110 °C for 5 h with stirring. Third, 2 mol % Pd(OAc)_2_, 4 mol % PCy_3_·HBF_4_, and another arylboronic acid (0.75 mmol) were added, and the reaction was continued for 12 h at 110 °C. After the reaction was completed, the crude mixture was allowed to reach room temperature. Finally, dichloromethane and a saturated solution of NaHCO_3_ were added, and the layers were separated. The aqueous phase was extracted three times with dichloromethane, and the combined organic layers were washed with two portions of a saturated NaHCO_3_ solution and one portion of brine, and then dried over anhydrous MgSO_4_ and filtered. The solvent was removed under reduced pressure. The products were purified by silica gel chromatography.

### 3.3. General Procedure for the Synthesis of ***3x*** and ***3y***

A solution of 4-acetylpyridine (0.5 mmol) and tosylhydrazide (0.75 mmol) in 4 mL of 1,4-dioxane was stirred at 80 °C for 3 h in a reaction tube. Then, potassium carbonate (1.5 mmol) and 4-bromophenylboronic acid (0.55 mmol) were added to the reaction mixture. The system was refluxed at 110 °C for 7 h with stirring. Third, 1 mol % Pd(OAc)_2_, 4 mol % PCy_3_·HBF_4_, and another arylboronic acid (0.75 mmol) were added, and the reaction was continued for 10 h at 110 °C. After the reaction was completed, the crude mixture was allowed to reach room temperature. Finally, dichloromethane and a saturated solution of NaHCO_3_ were added, and the layers were separated. The aqueous phase was extracted three times with dichloromethane, and the combined organic layers were washed with two portions of a saturated NaHCO_3_ solution and one portion of brine, and then dried over anhydrous MgSO_4_ and filtered. The solvent was removed under reduced pressure. The products were purified by silica gel chromatography.

*4-(1-Phenylethyl)-1,1′-biphenyl* (**3a**) [46]: White solid (121.43 mg, 94%). 60.8–62.0 °C. ^1^H NMR (400 MHz, CDCl_3_) (Figure S1) *δ* 7.55–7.48 (m, 4H), 7.39–7.35 (m, 2H), 7.29–7.24 (m, 7H), 7.19–7.14 (m, 1H), 4.16 (q, *J* = 7.2 Hz, 1H), 1.65 (d, *J* = 7.2 Hz, 3H). ^13^C NMR (101 MHz, CDCl_3_) (Figure S2) *δ* 146.4, 145.6, 141.1, 139.1, 128.9, 128.6, 128.2, 127.8, 127.3, 127.2, 127.1, 126.3, 44.6(CH), 22.0(CH_3_). IR (neat, cm^−1^): 3016, 2960, 2918, 2875, 1508, 806.

*4-Methyl-4′-(1-(p-tolyl)ethyl)-1,1′-biphenyl* (**3b**): White solid (121.72 mg, 85%), m.p. 48.0–49.0 °C. ^1^H NMR (400 MHz, CDCl_3_) (Figure S3) *δ* 7.47 (dd, *J* = 10.8, 8.4 Hz, 4H), 7.28–7.21 (m, 5H), 7.13 (dd, *J* = 18.1, 8.1 Hz, 3H), 4.15 (dd, *J* = 14.4, 7.2 Hz, 1H), 2.34 (d, *J* = 25.7 Hz, 6H), 1.65 (d, *J* = 7.2 Hz, 3H). ^13^C NMR (101 MHz, CDCl_3_) (Figure S4) *δ* 145.6, 143.5, 139.0, 138.3, 136.9, 135.7, 129.6, 129.2, 128.0, 127.6, 127.0, 44.2(CH_3_), 22.1(CH), 21.2(CHCH_3_). HRMS (EI) (Figure S5): *m*/*z* calcd. for C_22_H_22_ [M]: 286.1722, found [M]: 286.1718. IR (neat, cm^−1^): 3016, 2960, 2918, 2875, 1508, 806.

*4-Methoxy-4′-(1-(4-methoxyphenyl)ethyl)-1,1′-biphenyl* (**3c**): White solid (138.51 mg, 87%), m.p. 76.0–77.1 °C. ^1^H NMR (400 MHz, CDCl_3_) (Figure S6) *δ* 7.47 (d, *J* = 8.8 Hz, 4H), 7.16 (d, *J* = 8.0 Hz, 4H), 6.89 (d, *J* = 8.8 Hz, 4H), 4.13 (q, *J* = 7.2 Hz, 1H), 3.80 (s, 6H), 1.63 (d, *J* = 7.2 Hz, 3H). ^13^C NMR (101 MHz, CDCl_3_) (Figure S7) *δ* 159.1, 158.0, 145.4, 138.6, 133.7, 128.6, 128.1, 128.0, 126.8, 114.3, 113.9, 55.4(OCH_3_), 43.7(CH), 22.2(CH_3_). HRMS (EI) (Figure S8): *m*/*z* calcd. for C_22_H_22_O_2_ [M] 318.1620, found [M]: 318.1620. IR (neat, cm^−1^): 3003, 2968, 2928, 2831, 1603, 1508, 1248, 816.

*4-Fluoro-4′-(1-(4-fluorophenyl)ethyl)-1,1′-biphenyl* (**3d**): White solid (128.04 mg, 87%), m.p. 72.1–73.6 °C. ^1^H NMR (400 MHz, CDCl_3_) (Figure S9) *δ* 7.52–7.44 (m, 4H), 7.26–7.18 (m, 4H), 7.10 (t, *J* = 8.4 Hz, 2H), 6.98 (t, *J* = 8.4Hz, 2H), 4.17 (q, *J* = 7.2 Hz, 1H), 1.65 (d, *J* = 7.2 Hz, 3H). ^13^C NMR (101 MHz, CDCl_3_) (Figure S10) *δ* 163.7, 162.7, 161.3, 160.2, 145.5, 142.0, 138.3, 137.1, 129.1, 128.7, 128.1, 127.2, 115.5, 43.9(CH), 22.2(CH_3_). HRMS (EI) (Figure S11): *m*/*z* calcd. for C_20_H_16_F_2_ [M]: 294.1220, found [M]: 294.1222. IR (neat, cm^−1^): 3043, 2968, 2876, 1888, 1607, 1508, 1217, 1169, 822.

*4-(Trifluoromethyl)-4′-(1-(trifluoromethyl)phenyl)ethyl)-1,1′-biphenyl* (**3e**): White solid (151.73 mg, 77%), m.p. 60.0–61.2 °C. ^1^H NMR (400 MHz, CDCl_3_) (Figure S12) *δ* 7.67 (s, 4H), 7.57–7.52 (m, 4H), 7.3–7.30 (m, 4H) 4.27 (q, *J* = 7.2 Hz, 1H), 1.70 (d, *J* = 7.2 Hz,3H). ^13^C NMR (101 MHz, CDCl_3_) (Figure S13) *δ* 150.2 (d, *J* = 1.4 Hz), 145.6, 144.4, 138.0, 129.6, 129.3, 128.9, 128.4, 128.1, 127.5, 125.7, 123.1, 44.5(CH), 21.7(CH_3_). HRMS (EI) (Figure S14): *m*/*z* calcd. for C_22_H_16_F_6_ [M]: 394.1156, found [M]: 394.1160. IR (neat, cm^−1^): 3026, 2976, 2930, 1618, 1325, 1106, 828.

*4-Propyl-4′-(1-(4-propylphenyl)ethyl)-1,1′-biphenyl* (**3f**): Colorless oil (140.43 mg, 82%). ^1^H NMR (400 MHz, CDCl_3_) (Figure S15) *δ* 7.48–7.45 (m, 4H), 7.25 (d, *J* = 8.0 Hz, 2H), 7.19 (d, *J* = 8.0 Hz, 2H), 7.14 (d, *J* = 8.0 Hz, 2H), 7.08 (d, *J* = 8.0 Hz, 2H), 4.12 (q, *J* = 7.2 Hz, 1H), 2.60–2.51 (m, 4H), 1.68–1.58 (m, 7H), 0.96–0.90 (m, 6H). ^13^C NMR (101 MHz, CDCl_3_) (Figure S16) *δ* 145.5, 143.7, 141.6, 140.4, 139.0, 138.5, 129.5, 128.6, 128.1, 127.6, 127.0, 44.3(CHCH_3_), 37.8(CH_2_CH_2_CH_3_), 24.7 (CH_2_CH_2_CH_3_), 22.1(CHCH_3_), 14.1(CH_2_CH_2_CH_3_). HRMS (EI) (Figure S17): *m*/*z* calcd. for C_26_H_30_ [M]: 342.2348, found [M]: 342.2345. IR (film): 3018, 2965, 2926, 2868, 1500, 1452, 810.

*3-Methyl-4′-(1-(m-tolyl)ethyl)-1,1′-biphenyl* (**3g**): Colorless oil (134.61 mg, 94%). ^1^H NMR (400 MHz, CDCl_3_) (Figure S18) *δ* 7.49–7.47 (m, 2H), 7.35 (d, *J* = 8.4 Hz, 2H), 7.27 (t, *J* = 8.4 Hz, 3H), 7.19–6.98 (m, 5H), 4.13 (q, *J* = 7.2 Hz, 1H), 2.38 (s, 3H), 2.30 (s, 3H), 1.64 (d, *J* = 7.2Hz, 3H). ^13^C NMR (101 MHz, CDCl_3_) (Figure S19) *δ* 146.4, 145.6, 141.2, 139.1, 138.3, 138.0, 128.7, 128.6, 128.4, 128.1, 128.0, 127.9, 127.2, 127.0, 124.8, 124.3, 44.6(CHCH_3_), 22.0(CHCH_3_), 21.7(CH_3_). HRMS (EI) (Figure S20): *m*/*z* calcd. for C_22_H_22_ [M]: 286.1722, found [M]: 286.1721. IR (film): 3024, 2972, 2922, 2870, 1604, 1479, 835, 783, 700.

*3-Methoxy-4′-(1-(3-methoxyphenyl)-1,1′-biphenyl* (**3h**): Colorless oil (136.92 mg, 86%). ^1^H NMR (400 MHz, CDCl_3_) (Figure S21) *δ* 7.49 (dt, *J* = 4 Hz, 2Hz, 2H), 7.33–7.27 (m, 3H), 7.22–7.05 (m, 4H), 6.85 (dd, *J* = 7.4, 2.8 Hz, 2H), 6.81–6.80 (m, 1H), 4.14 (q, *J* = 7.2 Hz, 1H), 3.81 (s, 3H), 3.75 (s, 3H), 1.65 (d, *J* = 7.2 Hz, 3H). ^13^C NMR (101 MHz, CDCl_3_) (Figure S22) *δ* 160.0, 159.8, 148.0, 145.6, 142.6, 139.0, 131.5, 129.80, 129.5, 128.0, 127.3, 120.2, 119.7, 113.9, 112.9, 112.6, 111.1, 55.3(OCH_3_), 44.6(CH), 21.9(CH_3_). HRMS (EI) (Figure S23): *m*/*z* calcd. for C_22_H_22_O_2_ [M]: 318.1620, found [M]: 318.1616. IR (film): 2999, 2962, 2935, 2835, 1601, 1485, 1261, 1157, 835.

*3,4,5-Trifluoro-4′-(1-(3,4,5-trifluorophenyl)ethyl)-1,1′biphenyl* (**3i**): Colorless oil (102.56 mg, 56%). ^1^H NMR (400 MHz, CDCl_3_) (Figure S24) *δ* 7.45–7.42 (m, 2H), 7.25–7.14 (m, 4H), 6.87–6.79 (m, 2H), 4.12 (q, *J* = 7.2 Hz, 1H), 1.63 (d, *J* = 7.2 Hz, 3H). ^13^C NMR (101 MHz, CDCl_3_) (Figure S25) *δ* 152.6, 150.2, 145.2, 142.4, 140.6, 139.6, 138.1, 137.0, 128.3, 127.3, 111.6, 111.0, 44.0(CH), 21.6(CH_3_). HRMS (EI) (Figure S26): *m*/*z* calcd. for C_20_H_12_F_6_ [M]: 366.0843, found [M]: 366.0844. IR (film): 3042, 2968, 2926, 1618, 1537, 1445, 1348, 1244, 1041, 831.

*2-Methyl-4′-(1-(o-tolyl)ethyl)-1,1′-biphenyl* (**3j**): Colorless oil (131.75 mg, 92%). ^1^H NMR (400 MHz, CDCl_3_) (Figure S27) *δ* 7.31 (d, *J* = 7.6 Hz, 1H), 7.23–7.19 (m, 9H), 7.15–7.13 (m, 2H), 4.36 (q, *J* = 7.2 Hz, 1H), 2.26 (d, *J* = 10.8 Hz, 6H), 1.65 (d, *J* = 7.2 Hz, 3H). ^13^C NMR (101 MHz, CDCl_3_) (Figure S28) *δ* 144.8, 144.1, 141.9, 139.5, 136.2, 135.5, 130.5, 130.0, 129.2, 127.4, 127.2, 126.9, 126.2, 125.8, 40.8(CHCH_3_), 22.2(CHCH_3_), 20.7(CH_3_), 20.0(CH_3_). HRMS (EI) (Figure S29): *m*/*z* calcd. for C_22_H_22_ [M]: 286.1722, found [M]: 286.1724. IR (film): 3017, 2968, 2926, 2866, 1601, 1481, 835, 756.

*2-(4-(1-(Naphthalene-2-yl)ethyl)phenyl)naphthalene* (**3k**): White solid (148.77 mg, 83%), m.p. 110.9–111.5 °C. ^1^H NMR (400 MHz, CDCl_3_) (Figure S30) *δ* 8.03 (d, *J* = 16.8 Hz, 1H), 7.90–7.73 (m, 8H), 7.71–7.64 (m, 2H), 7.50–7.44 (m, 4H), 7.41–7.36 (m, 3H), 4.38 (dd, *J* = 13.9, 6.8 Hz, 1H), 1.80 (dd, *J* = 12.5, 7.0 Hz, 3H). ^13^C NMR (101 MHz, d_6_-DMSO) (Figure S31) *δ* 145.6, 143.7, 137.7, 137.3, 133.3, 133.1, 132.1, 131.7, 128.4, 128.1, 127.9, 127.6, 127.5, 127.4, 127.0, 126.6, 126.3, 126.1, 126.0, 125.5, 125.0, 124.9, 43.8(CH), 21.3(CH_3_). HRMS (EI) (Figure S32): *m*/*z* calcd. for C_28_H_22_ [M]: 358.1722, found [M]: 358.1718. IR (neat, cm^−1^): 3053, 2960, 2870, 1601, 1501, 1448, 860,817.

*3-Methyl-3-(1-(m-tolyl)ethyl-1,1-biphenyl* (**3l**): Colorless oil (117.42 mg, 82%). ^1^H NMR (400 MHz, CDCl_3_) (Figure S33) *δ* 7.45 (t, *J* = 1.7 Hz, 1H), 7.40–7.27 (m, 5H), 7.19–7.12 (m, 3H), 7.06–6.97 (m, 3H), 4.16 (q, *J* = 7.2 Hz, 1H), 2.34 (d, *J* = 37.8 Hz, 6H), 1.66 (d, *J* = 7.2 Hz, 3H). ^13^C NMR (101 MHz, CDCl_3_) (Figure S34) *δ* 147.0, 146.4, 141.6, 141.5, 138.4, 138.0, 128.8, 128.7, 128.6, 128.4, 128.1, 127.0, 126.7, 126.6, 125.1, 124.7, 124.5, 45.0(CH), 22.1(CHCH_3_), 21.7(CH_3_), 21.6(CH_3_). HRMS (EI) (Figure S35): *m*/*z* calcd. for C_22_H_22_ [M]: 286.1722, found [M]: 286.1724. IR (film): 3036, 2961, 2913, 2879, 1591, 1475, 791, 700.

*3-Methyl-4′-(3-methylbenzyl)-1,1′-biphenyl* (**3m**) [47]: Colorless oil (100.78 mg,74%). ^1^H NMR (400 MHz, CDCl_3_) (Figure S36) *δ* 7.50 (dt, *J* = 4.0, 2.0 Hz, 2H), 7.37–7.25 (m, 4H), 7.23–7.12 (m, 3H), 7.02 (d, *J* = 7.7 Hz, 3H), 3.97 (s, 2H), 2.40 (s, 3H), 2.32 (s, 3H). ^13^C NMR (101 MHz, CDCl_3_) (Figure S37) *δ* 141.1, 140.4, 139.2, 138.4, 138.2, 129.8, 129.4, 128.8, 128.5, 127.95, 127.3, 127.0, 126.1, 124.2, 41.7(CH), 21.7(CHCH_3_), 21.6(CH_3_). HRMS (EI) (Figure S38): *m*/*z* calcd. for C_21_H_20_ [M]: 272.1565, found [M]: 272.1560. IR (film): 3030, 2916, 2858, 1606, 1479, 783.

*3′-Methyl-3-(2-methylbenzyl)-1,1′-biphenyl* (**3n**) [47]: Colorless oil (39.50 mg, 29%). ^1^H NMR (400 MHz, CDCl_3_) (Figure S39) *δ* 7.28–7.19 (m, 5H), 7.14–7.04 (m, 4H), 6.95 (d, *J* = 7.0 Hz, 1H), 6.79 (d, *J* = 5.6 Hz, 2H), 3.90 (s, 2H), 2.34 (s, 3H), 2.25 (s, 3H). ^13^C NMR (101 MHz, CDCl_3_) (Figure S40) *δ* 142.5, 141.7, 141.6, 138.5, 137.8, 137.6, 130.3, 130.1, 129.8, 128.2, 128.0, 127.7, 127.5, 126.6, 126.5, 126.2, 126.1, 39.2(CH), 21.5(CH_3_). HRMS (EI) (Figure S41): *m*/*z* calcd. for C_21_H_20_ [M]: 272.1565, found [M]: 272.1566. IR (film): 3022, 2914, 2858, 1597, 1485, 762, 698.

*4-Methoxy-4′-(1-(p-tolyl)ethyl)-1,1′-biphenyl* (**3o**): White solid (120.96 mg, 80%), m.p. 99.2–100.7 °C. ^1^H NMR (400 MHz, CDCl_3_) (Figure S42) *δ* 7.47 (dd, *J* = 13.7, 8.5 Hz, 4H), 7.27–7.24 (m, 2H), 7.13 (q, *J* = 8.2 Hz, 4H), 6.97–6.92 (m, 2H), 4.14 (q, *J* = 7.2 Hz, 1H), 3.83 (s, 3H), 2.31 (s, 3H), 1.65 (d, *J* = 7.2 Hz, 3H). ^13^C NMR (101 MHz, CDCl_3_) (Figure S43) *δ* 159.1, 145.2, 143.5, 138.6, 135.7, 133.8, 129.2, 128.1, 127.6, 126.8, 114.3, 55.5(OCH_3_), 44.2(CH), 22.1(CHCH_3_), 21.1(CH_3_). HRMS (EI) (Figure S44): *m*/*z* calcd. for C_22_H_22_O [M]: 302.1671, found [M]: 302.1666. IR (neat, cm^−1^): 3034, 2968, 2905, 1606, 1501, 1454, 1248, 1035, 820.

*4-Propyl-4′-(1-(p-tolyl)ethyl)-1,1′-biphenyl* (**3p**): Colorless oil (136.92 mg, 86%). ^1^H NMR (400 MHz, CDCl_3_) (Figure S45) *δ* 7.48–7.44 (m, 4H), 7.25–7.23 (m, 2H), 7.20–7.18 (m, 2H), 7.14–7.11 (m, 2H), 7.07 (dd, *J* = 8.0, 2.3 Hz, 2H), 4.15–4.09 (m, 1H), 2.65–2.51 (m, 2H), 2.28 (d, *J* = 2.4 Hz, 3H), 1.64–1.61 (m, 5H), 0.96–0.92(m, 3H). ^13^C NMR (101 MHz, CDCl_3_) (Figure S46) *δ* 145.5, 143.5, 141.7, 139.0, 138.5, 135.6, 129.2, 128.9, 128.0, 127.6, 127.0, 126.9, 44.2(CH), 37.8(CH_3_), 24.7(CH_2_CH_2_CH_3_), 22.1(CHCH_3_), 21.1(CH_2_CH_2_CH_3_), 14.0(CH_2_CH_2_CH_3_). HRMS (EI) (Figure S47): *m*/*z* calcd. for C_24_H_26_ [M]: 314.2035, found [M]: 314.2029. IR (film): 3024, 2960, 2928, 2866, 1502, 808.

*4-Flouro-4′-(1-(p-toly)etyl)-1,1′-biphenyl* (**3q**): White solid (98.73 mg, 68%), m.p. 85.2–86.0 °C. ^1^H NMR (400 MHz, CDCl_3_) (Figure S48) *δ* 7.52–7.48 (m, 2H), 7.44 (d, *J* = 8.4 Hz, 2H), 7.28–7.24 (m, 2H), 7.15–7.07 (m, 6H), 4.15 (q, *J* = 7.2 Hz, 1H), 2.31 (s, 3H), 1.65 (d, *J* = 7.2 Hz, 3H). ^13^C NMR (101 MHz, CDCl_3_) (Figure S49) *δ* 162.5, 160.1, 144.7, 142.2, 136.8, 136.1, 134.6, 128.1, 127.5, 127.0, 126.4, 125.9, 114.6, 114.4, 43.0(CH), 20.9(CHCH_3_), 20.0(CH_3_). HRMS (EI) (Figure S50): *m*/*z* calcd. for C_21_H_19_F [M]: 290.1471, found [M]: 290.1472. IR (neat, cm^−1^): 3032, 2966, 2924, 2870, 1597, 1500, 1240, 820.

*4-(1-(p-Tolyl)ethyl)-4′-(trifluoromethyl)-1,1′-biphenyl* (**3r**): Colorless oil (85.10 mg, 50%). ^1^H NMR (400 MHz, CDCl_3_) (Figure S51) *δ* 7.65 (s, 4H), 7.50 (dt, *J* = 4.0, 2.0 Hz, 2H), 7.31 (d, *J* = 8.0 Hz, 2H), 7.16–7.07 (m, 4H), 4.16 (q, *J* = 7.2 Hz, 1H), 2.31 (s, 3H), 1.66 (d, *J* = 7.2 Hz, 3H). ^13^C NMR (101 MHz, CDCl_3_) (Figure S52) *δ* 146.9, 144.5 (d, *J* = 1.6 Hz), 143.0, 137.4, 135.7, 131.4, 129.2, 128.2, 127.4, 127.2, 125.6, 123.0(CF_3_), 44.1(CH), 21.9(CH_3_), 21.0(CHCH_3_). HRMS (EI) (Figure S53): *m*/*z* calcd. for C_22_H_19_F_3_ [M]: 340.1439, found [M]: 340.1441. IR (film): 3021, 2966, 2926, 2882, 1618, 1504, 1325, 1169, 1121, 829.

*2,4-Difluoro-4′-(1-(p-tolyl)ethyl)-1,1′-biphenyl* (**3s**): Colorless oil (112.47 mg, 73%). ^1^H NMR (400 MHz, CDCl_3_) (Figure S54) *δ* 7.40 (dd, *J* = 8.2, 1.7 Hz, 2H), 7.35–7.27 (m, 3H), 7.12 (q, *J* = 8.3 Hz, 4H), 6.93–6.84 (m, 2H), 4.15 (q, *J* = 7.2 Hz, 1H), 2.31 (s, 3H), 1.64 (d, *J* = 7.2 Hz, 3H). ^13^C NMR (101 MHz, CDCl_3_) (Figure S55) *δ* 163.4, 161.0, 158.6, 146.3, 143.1, 135.7, 132.6, 131.3, 129.2, 127.7, 127.5, 125.2, 111.5, 104.3, 44.2(CH), 21.9(CHCH_3_), 21.0(CH_3_). HRMS (EI) (Figure S56): *m*/*z* calcd. for C_21_H_18_F_2_ [M]: 308.1377, found [M]: 308.1371. IR (film): 3024, 2965, 2924, 2868, 1607, 1495, 1139, 848, 812.

*9-(4-(1-([1,1'-Biphenyl]-4-yl)ethyl)phenyl)-9H-carbazole* (**3t**): White solid (67.77 mg, 32%), m.p. 141.2–143.0 °C. ^1^H NMR (400 MHz, CDCl_3_) (Figure S57) *δ* 8.17 (dt, *J* = 7.8, 1.1 Hz, 2H), 7.67–7.60 (m, 4H), 7.57–7.34 (m, 13H), 7.34–7.28 (m, 2H), 4.36 (q, *J* = 7.2 Hz, 1H), 1.81 (d, *J* = 7.2 Hz, 3H). ^13^C NMR (101 MHz, CDCl_3_) (Figure S58) δ 145.6, 145.0, 140.9, 139.3, 135.6, 129.0, 128.8, 128.1, 127.1, 125.9, 123.3, 120.3, 119.8, 109.9, 44.3(CH), 22.0(CH_3_). HRMS (EI) (Figure S59): *m*/*z* calcd. for C_32_H_25_N [M]^+^: 423.1987, found [M]^+^:423.1982. IR (neat, cm^-1^): 3051, 2963, 2876, 1595, 1510, 1232, 835, 812.

*4′-Methoxy-3-(1-(p-tolyl)ethyl)-1,1′-biphenyl* (**3u**): Colorless oil (63.51 mg, 42%). ^1^H NMR (400 MHz, CDCl_3_) (Figure S60) *δ* 7.51–7.47 (m, 2H), 7.40 (t, *J* = 1.8 Hz, 1H), 7.36 (dt, *J* = 7.7, 1.5 Hz, 1H), 7.31 (t, *J* = 7.6 Hz, 1H), 7.12 (dd, *J* = 23.2, 8.4 Hz, 5H), 6.97–6.93 (m, 2H), 4.16 (q, *J* = 7.2 Hz, 1H), 3.83 (s, 3H), 2.30 (s, 3H), 1.66 (d, *J* = 7.2 Hz, 3H). ^13^C NMR (101 MHz, CDCl_3_) (Figure S61) *δ* 159.2, 147.2, 143.4, 141.0, 135.6, 134.1, 129.2, 128.8, 128.3, 127.6, 126.3, 126.1, 124.6, 114.3, 55.5(OCH_3_), 44.6(CH), 22.1(CHCH_3_), 21.1(CH_3_). HRMS (EI) (Figure S62): *m*/*z* calcd. for C_22_H_22_O [M]: 302.1671, found [M]: 302.1665. IR (film): 3003, 2930, 2682, 1612, 1520, 1244, 818.

*4-Methoxy-4′-(4-methylbenzyl)-1,1′-biphenyl* (**3v**): White solid (33.16 mg, 23%), m.p. 94.7–96.6 °C. ^1^H NMR (400 MHz, CDCl_3_) (Figure S63) *δ* 7.47 (dd, *J* = 14.7, 8.5 Hz, 4H), 7.22 (d, *J* = 8.3 Hz, 2H), 7.11 (s, 4H), 6.95 (d, *J* = 8.8 Hz, 2H), 3.97 (s, 2H), 3.84 (s, 3H), 2.32 (s, 3H). ^13^C NMR (101 MHz, CDCl_3_) (Figure S64) *δ* 159.0, 139.9, 138.6, 138.0, 135.6, 133.6, 129.19, 128.8, 128.0, 126.8, 114.1, 55.3(OCH_3_), 41.1(CH_2_), 21.0(CH_3_). HRMS (EI) (Figure S65): *m*/*z* calcd. for C_21_H_20_O [M]: 288.1514, found [M]: 288.1513. IR (neat, cm^−1^): 2957, 2912, 2837, 1607, 1504, 1248, 800.

*4′-Methoxy-2-(4-methylbenzyl)-1,1′-biphenyl* (**3w**) [48]: Colorless oil (36.05 mg, 25%). ^1^H NMR (400 MHz, CDCl_3_) (Figure S66) *δ* 7.25–7.23 (m, 3H), 7.20–7.17 (m, 3H), 7.02 (d, *J* = 7.9 Hz, 2H), 6.92–6.88 (m, 4H), 3.91 (s, 2H), 3.83 (s, 3H), 2.29 (s, 3H). ^13^C NMR (101 MHz, CDCl_3_) (Figure S67) *δ* 158.7, 141.9, 138.7, 138.6, 135.3, 134.2,130.5, 130.4, 129.1, 128.9, 127.3, 126.2, 113.6, 55.4(OCH_3_), 38.7(CH_2_), 21.1(CH_3_). HRMS (EI) (Figure S68): *m*/*z* calcd. for C_21_H_20_O [M]: 288.1514, found [M]: 288.1519. IR (film): 3018, 2924, 2849, 1609, 1246, 764.

*4-(1-([1,1′-Biphenyl]-4-yl)ethyl)pyridine* (**3x**): White solid (77.80 mg, 60%), m.p. 55.8–56.5 °C. ^1^H NMR (400 MHz, CDCl_3_) (Figure S69) *δ* 8.53 (d, *J* = 5.6 Hz, 2H), 7.58–7.53 (m, 4H), 7.42 (t, *J* = 7.5 Hz, 2H), 7.33 (d, *J* = 7.3, 1H), 7.27–7.24 (m, 2H), 7.19 (dd, *J* = 4.8, 1.2 Hz, 2H), 4.17 (q, *J* = 7.2 Hz, 1H), 1.68 (d, *J* = 7.2 Hz, 3H). ^13^C NMR (101 MHz, CDCl_3_) (Figure S70) *δ* 155.7, 149.5, 143.4, 140.8, 139.8, 128.9, 128.1, 127.5, 127.4, 127.1, 123.3, 44.1(CH), 21.2(CH_3_). HRMS (ESI) (Figure S71): *m*/*z* calcd. for C_19_H_18_N [M + H]^+^: 260.1439, found [M + H]^+^: 260.1432. IR (neat, cm^−1^): 3352, 2885, 1591, 1404, 839.

*4-(1-(4′-Methoxy-[1,1′-biphenyl]-4-yl)ethyl)pyridine* (**3y**): Light yellow solid (73.80 mg, 51%), m.p. 55.8–56.5 °C. ^1^H NMR (400 MHz, CDCl_3_) (Figure S72) *δ* 8.51 (d, *J* = 6.0 Hz, 2H), 7.49 (m, 4H), 7.19 (m, 4H), 6.95 (d, *J* = 8.8 Hz, 2H), 4.13 (q, *J* = 7.2 Hz, 1H), 3.81 (s, 3H), 1.65 (d, *J* = 7.2 Hz, 3H). ^13^C NMR (101 MHz, CDCl_3_) (Figure S73) *δ* 159.2, 155.6, 149.5, 142.7, 139.3, 133.3, 128.1, 127.0, 123.2, 114.3, 55.4(OCH_3_), 44.0(CH), 21.1(CH_3_). HRMS (ESI) (Figure S74): *m*/*z* calcd. for C_20_H_20_NO [M + H]^+^: 290.1545, found [M + H]^+^: 290.1534. IR (neat, cm^−1^): 3035, 2970, 2843, 1604, 1497, 1248, 1028, 814.

## 4. Conclusions

In summary, we developed an operationally simple, efficient general procedures for synthesizing 4-benzyl-1,1′-biphenyl derivatives, via a four-component one-pot reaction of 4-bromoacetophenone, tosylhydrazide and two arylboronic acids as the starting materials, involving Barluenga and Suzuki couplings. This method can be processed in one-pot with multiple steps; indeed it is a simpler and more efficient way to synthesis 4-benzyl-1,1′-biphenyl compounds. Also, we demonstrated the utility of this method from commercially available starting materials to afford the corresponding products in moderate to excellent yields with good functional group tolerance. Notably, this protocol also proves to be suitable for synthesizing 4-pyridyl biphenylmethane-type compounds, which are analogs of CPY17 inhibitors.

## Figures and Tables

**Table 1:**
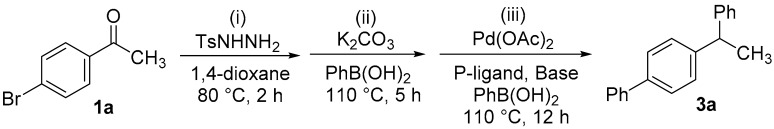
Optimization of one-pot, multi-step reaction conditions ^a^.

Entry	Pd(OAc)_2_	Ligand	PhB(OH)_2_	Base	Yield (%) ^b^
1	1 mol %	-	2.5	K_2_CO_3_	15
2	1 mol %	PPh_3_	2.5	K_2_CO_3_	61
3	1 mol %	PCy_3_∙HBF_4_	2.5	K_2_CO_3_	78
4	1 mol %	PCy_3_∙HBF_4_	2.5	Et_3_N	63
5	1 mol %	PCy_3_∙HBF_4_	2.5	K_3_PO_4_∙H_2_O	71
6	1 mol %	PCy_3_∙HBF_4_	2.5	NaOH	72
7	1 mol %	PCy_3_∙HBF_4_	2.5	Cs_2_CO_3_	28
8 ^c^	1 mol %	PCy_3_∙HBF_4_	3.0	K_2_CO_3_	89
9	2 mol %	PCy_3_∙HBF_4_	3.0	K_2_CO_3_	90
10 ^d^	1 mol %	PCy_3_∙HBF_4_	3.0	K_2_CO_3_	52

^a^ Reaction condition: (i) 0.5 mmol 4-bromoacetophenone **1a**, 0.75 mmol tosylhydrazide, 5 mL 1,4-dioxane, 80 °C, 2 h; (ii) 0.75 mmol K_2_CO_3_, phenylboronic acid (2.5 or 3.0 equivalent), 110 °C, 5 h; (iii) 1–2 mol % Pd(OAc)_2_, 4 mol % ligand, 0.75 mmol base, 110 °C, 12 h. ^b^ Isolated yield. ^c^ 0.75 mmol tosylhydrazide was used. ^d^ The Suzuki coupling reaction was carried out at 80 °C.

## Supplementary Materials

Supplementary materials are available online. the charts of ^1^H-, ^13^C-NMR and HRMS of products.

## References

[1] Tanabe K., Hölderich W.F. (1999). Industrial application of solid acid-base catalysts. Appl. Catal. A.

[2] Beltrame P., Zuretti G., Demartin F. (2000). Benzylation of biphenyl with benzyl chloride over crystalline, amorphous, and MCM-41 solid acid catalysts. Ind. Eng. Chem. Res..

[3] Hino M., Arata K. (1981). The synthesis of thermally stable oils by the benzylation of biphenyl with benzyl chloride catalyzed by Iron(III) oxide. Bull. Chem. Soc. Jpn..

[4] Huhtaniemi I., Nikula H., Parvinen M., Rannikko S. (1988). Histological and functional changes of the testis tissue during GnRH agonist treatment of prostatic cancer. Am. J. Clin. Oncol..

[5] Titus M.A., Schell M.J., Lih F.B., Tomer K.B., Mohler J.L. (2005). Testosterone and dihydrotestosterone tissue levels in recurrent prostate cancer. Clin. Cancer Res..

[6] Stanbrough M., Bubley G.J., Ross K., Golub T.R., Rubin M.A., Penning T.M., Febbo P.G., Balk S.P. (2006). Increased expression of genes converting adrenal androgens to testosterone in androgen-independent prostate cancer. Cancer Res..

[7] Montgomery R.B., Mostaghel E.A., Vessella R., Hess D.L., Kalhorn T.F., Higano C.S., True L.D., Nelson P.S. (2008). Maintenance of intratumoral androgens in metastatic prostate cancer: A mechanism for castration-resistant tumor growth. Cancer Res..

[8] Holzbeierlein J., Lal P., La Tulippe E., Smith A., Satagopan J., Zhang L., Ryan C., Smith S., Scher H., Scardino P. (2004). Gene expression analysis of human prostate carcinoma during hormonal therapy identifies androgen-responsive genes and mechanisms of therapy resistance. Am. J. Pathol..

[9] Labrie F., Dupont A., Belanger A., Cusan L., Lacourciere Y., Monfette G., Laberge J.G., Emond J.P., Fazekas A.T., Raynaud J.P. (1982). New hormonal therapy in prostatic carcinoma: Combined treatment with an LHRH agonist and an antiandrogen. Clin. Investig. Med..

[10] Hu Q.Z., Yin L., Jagusch C., Hille U.E., Hartmann R.W. (2010). Isopropylidene substitution increases activity and selectivity of biphenylmethylene 4-pyridine type CYP17 inhibitors. J. Med. Chem..

[11] Hu Q.Z., Jagusch C., Hille U.E., Haupenthal J., Hartmann R.W. (2010). Replacement of imidazolyl by pyridyl in biphenylmethylenes results in selective CYP17 and dual CYP17/CYP11B1 inhibitors for the treatment of prostate cancer. J. Med. Chem..

[12] Yadav G.D., George G. (2009). Monoalkylation of biphenyl over modified heteropoly acids: Novelty of cesium substituted dodecatungstophosphoric acid supported on hexagonal mesoporous silica. Catal. Today.

[13] Beltrame P., Demartin F., Zuretti G. (2001). An improved kinetic model for the reaction of biphenyl with benzyl chloride over an MCM-41 solid acid catalyst. Appl. Catal. A Gen..

[14] Jana S.K. (2005). Recent developments of heterogeneous solid catalysts for liquid-phase Friedel-Crafts type benzylation reaction. Catal. Surv. Asia.

[15] Haase J., Hillner K., Brueggemann W., Momm G. (1991). p-Benzylbiphenyl. Eur. Pat. Appl. Eur. Patent.

[16] Sakura K., Takeuchi H., Furumoto M. (1991). Manufacture of Benzylbiphenyls. Jpn. Kokai Tokkyo Koho JP.

[17] Beltrame P., Zuretti G. (1997). Benzylation of biphenyl with benzyl chloride over HY zeolites: A kinetic model for reaction and catalyst deactivation. Ind. Eng. Chem. Res..

[18] Miyaura N., Yanagi T., Suzuki A. (1981). The palladium-catalyzed cross-coupling reaction of phenylboronic acid with haloarenes in the presence of bases. Synth. Commun..

[19] Miyaura N., Suzuki A. (1995). Palladium-catalyzed cross-coupling reactions of organoboron compounds. Chem. Rev..

[20] Suzuki A. (1999). Recent advances in the cross-coupling reactions of organoboron derivatives with organic electrophiles, 1995–1998. J. Organomet. Chem..

[21] Suzuki A. (2002). Cross-coupling reactions via organoboranes. J. Organomet. Chem..

[22] Langle S., Abarbri M., Duchêne A. (2003). Selective double Suzuki cross-coupling reactions. Synthesis of unsymmetrical diaryl (or heteroaryl) methanes. Tetrahedron Lett..

[23] Henry N., Enguehard-Gueiffier C., Thery I., Gueiffier A. (2008). One-pot dual substitutions of bromobenzyl chloride, 2-chloromethyl-6-halogenoimidazo [1,2-*a*] pyridine and-[1,2-*b*]pyridazine by Suzuki-Miyaura cross-coupling reactions. Eur. J. Org. Chem..

[24] Zhu J., Bienaym H. (2005). Multicomponent Reactions.

[25] Dömling A. (2006). Recent developments in isocyanide based multicomponent reactions in applied chemistry. Chem. Rev..

[26] Gu Y.L. (2012). Multicomponent reactions in unconventional solvents: State of the art. Green Chem..

[27] Yang T., Cui H., Zhang C.H., Zhang L., Su C.Y. (2013). From homogeneous to heterogeneous catalysis of the three-component coupling of oxysulfonyl azides, alkynes, and amines. ChemCatChem.

[28] Fulton J.R., Aggarwal V.K., de Vicente J. (2005). The use of tosylhydrazone salts as a safe alternative for handling diazo compounds and their applications in organic synthesis. Eur. J. Org. Chem..

[29] Barluenga J., Valdés C. (2011). Tosylhydrazones: New uses for classic reagents in palladium-catalyzed cross-coupling and metal-free reactions. Angew. Chem. Int. Ed..

[30] Shao Z., Zhang H. (2012). *N*-tosylhydrazones: Versatile reagents for metal-catalyzed and metal-free cross-coupling reactions. Chem. Soc. Rev..

[31] Kai X., Chong S., Shang S. (2015). Progress of cross-coupling reaction with *N*-tosylhydrazones. Chin. J. Org. Chem..

[32] Liu Z., Zhang Y., Wang J. (2013). Transition-metal-catalyzed cross-coupling reaction with *N*-tosylhydrazones. Chin. J. Org. Chem..

[33] Xiao Q., Zhang Y., Wang J. (2013). Diazo compounds and *N*-tosylhydrazones: Novel cross-coupling partners in transition-metal-catalyzed reactions. Acc. Chem. Res..

[34] Jadhav A.P., Ray D., BhaskaraRao V.U., Singh R.P. (2016). Copper-catalyzed direct cross-coupling of compounds containing activated C–H/heteroatom–H bonds with *N*-tosylhydrazones. Eur. J. Org. Chem..

[35] Barluenga J., Tomás-Gamasa M., Aznar F., Valdés C. (2009). Metal-free carbon–carbon bond-forming reductive coupling between boronic acids and tosylhydrazones. Nat. Chem..

[36] Zhou L., Shi Y., Xiao Q., Liu Y., Ye F., Zhang Y., Wang J. (2011). CuBr-Catalyzed coupling of *N*-tosylhydrazones and terminal alkynes: Synthesis of benzofurans and indoles. Org. Lett..

[37] Ye F., Shi Y., Zhou L., Xiao Q., Zhang Y., Wang J. (2011). Expeditious synthesis of phenanthrenes via CuBr_2_-catalyzed coupling of terminal alkynes and *N*-tosylhydrazones derived from *O*-formyl biphenyls. Org. Lett..

[38] Zhou L., Xiao T., Dong X. (2013). Benzofuran and indole synthesis via Cu(I)-catalyzed coupling of *N*-tosylhydrazone and *o*-hydroxy or *o*-amino phenylacetylene. Org. Biomol. Chem..

[39] Zhou L., Ye F., Zhang Y., Wang J. (2010). Pd-catalyzed three-component coupling of *N*-tosylhydrazone, terminal alkyne, and aryl halide. J. Am. Chem. Soc..

[40] Roche M., Hamze A., Brion J.-D., Alami M. (2013). Catalytic three-component one-pot reaction of hydrazones, dihaloarenes, and amines. Org. Lett..

[41] Naret T., Retailleau P., Bignon J., Brion J.-D., Alami M., Hamze A. (2016). Palladium-catalyzed one-pot synthesis of 5-(1-arylvinyl)-1*H*-benzimidazoles: Overcoming the limitation of acetamide partners. Adv. Synth. Catal..

[42] Shen X., Gu N., Liu P., Ma X., Xie J., Liu Y., He L., Dai B. (2015). A simple and efficient synthesis of 9-arylfluorenes via metal-free reductive coupling of arylboronic acids and *N*-tosylhydrazones in situ. RSC Adv..

[43] Shen X., Gu N., Liu P., Ma X., Xie J., Liu Y., Dai B. (2016). One-pot synthesis of triarylmethanes via metal-free reductive coupling of diaryl ketones, tosylhydrazide, and arylboronic acids. Chin. J. Chem..

[44] Shen X., Liu P., Liu Y., Liu Y., Dai B. (2017). One-pot reductive coupling reactions of acetyl naphthalene derivatives, tosylhydrazide, with arylboronic acids. Tetrahedron.

[45] Liu Y., Liu P., Liu Y., Wei Y. (2017). Pd(0)-catalyzed tandem one-pot reaction of biphenyl ketones/aldehydes to the corresponding di-substituted aryl olefins. Chin. J. Chem..

[46] López-Pérez A., Adrio J., Carretero J.C. (2009). Palladium-catalyzed cross-coupling reaction of secondary benzylic bromides with Grignard reagents. Org. Lett..

[47] Zhang Y., Feng M.T., Lu J.M. (2013). *N*-Heterocyclic carbine-palladium (ii)-1-methylimidazole complex catalyzed Suzuki-Miyaura coupling of benzylic chlorides with arylboronic acids or potassium phenyltrifluoroborate in neat water. Org. Biomol. Chem..

[48] Anselmi S.E., Abarbri M., Duchêne A., Langle-Lamande S., Thibonnet J. (2012). Efficient synthesis of substituted styrenes and biaryls (or heteroaryls) with regioselective reactions of ortho-, meta-, and para-bromobenzyl bromide. Synthesis.

