# Supplementary material for: Multi-Component One-Pot Reaction of Aromatic Carbonyl Compounds, Tosylhydrazide, and Arylboronic Acids
Source: Molecules. 2017 Dec 7;22(12):2168. doi: 10.3390/molecules22122168 (PMC6149723; doi:10.3390/molecules22122168)
Supplement: Supplementary file 1 [file molecules-22-02168-s001.pdf]

Supporting Information for

Multi-Component One-Pot Reaction of Aromatic Carbonyl Compounds, Tosylhydrazide, and Arylboronic Acids

Ningning Gu, Yu Wei, Ping Liu *, Yan Liu * and Bin Dai

Key Laboratory for Green Processing of Chemical Engineering of Xinjiang Bingtuan, School of Chemistry and Chemical Engineering, Shihezi University, Shihezi 832003, China; tenggeli116@126.com (N.G.); yuweichem@126.com (Y.W.); db_tea@shzu.edu.cn (B.D.)

* Correspondence: liuping1979112@aliyun.com (P.L.); liuyan1979810@aliyun.com (Y.L.); Tel.: +86-0993-2057213 (P.L.)

Copies of ^{1}H NMR, ^{13}C NMR and HRMS spectra…………S2–S38

4-(1-phenylethyl)-1,1'-biphenyl (3a)

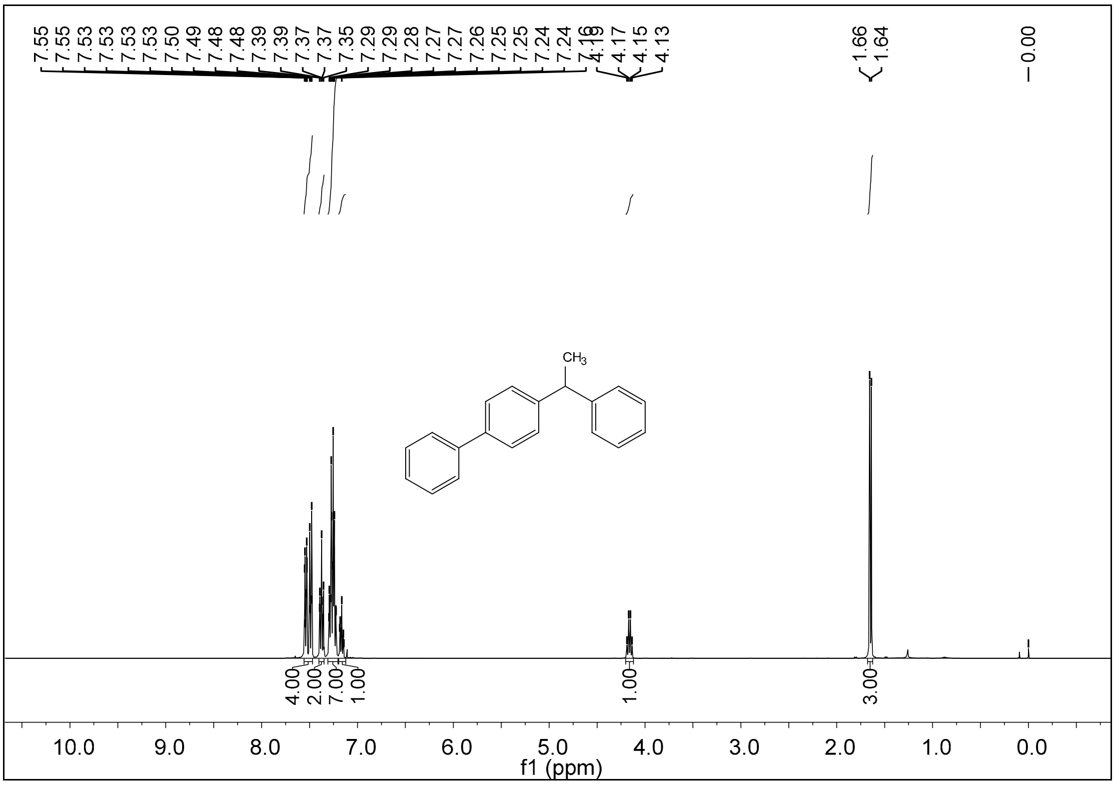

Figure S1

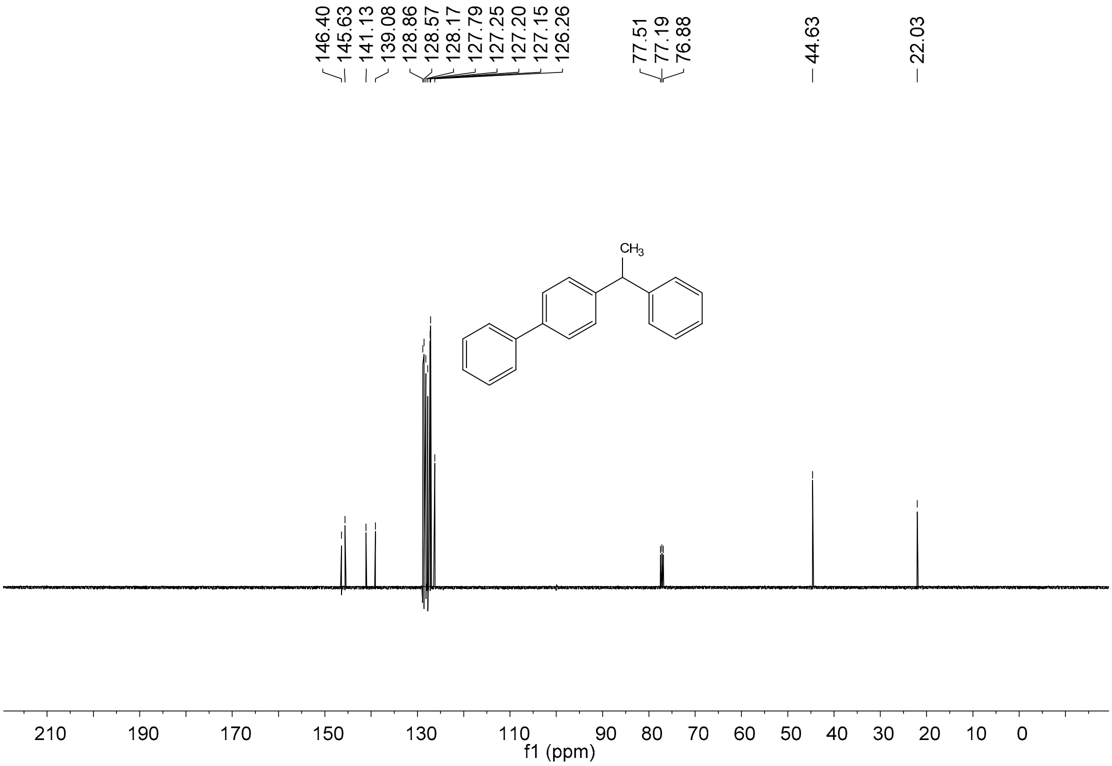

Figure S2

4-methyl-4'-(1-(p-tolyl)ethyl)-1,1'-biphenyl (3b)

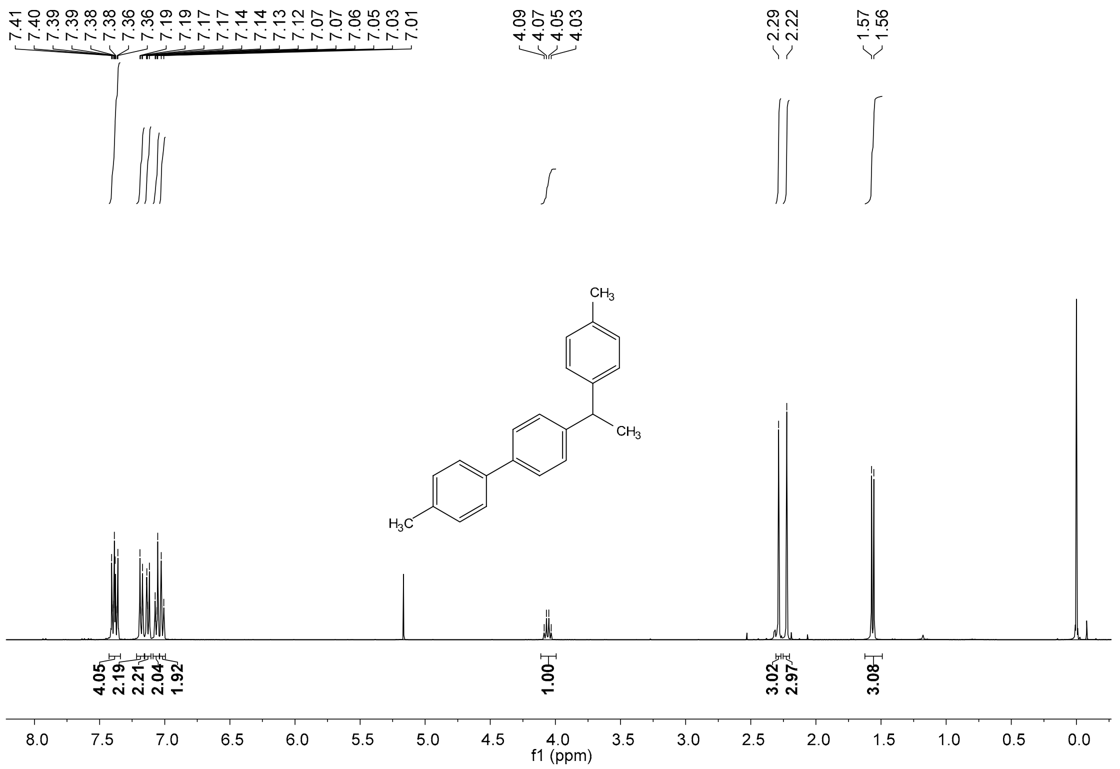

Figure S3

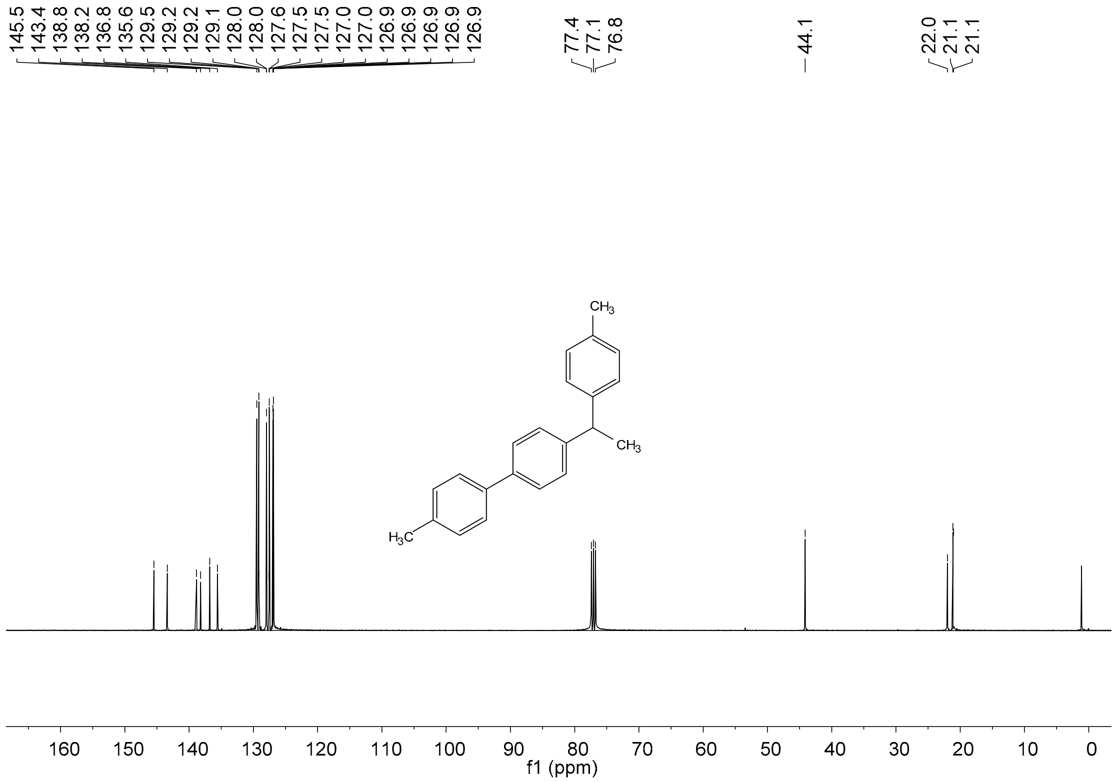

Figure S4

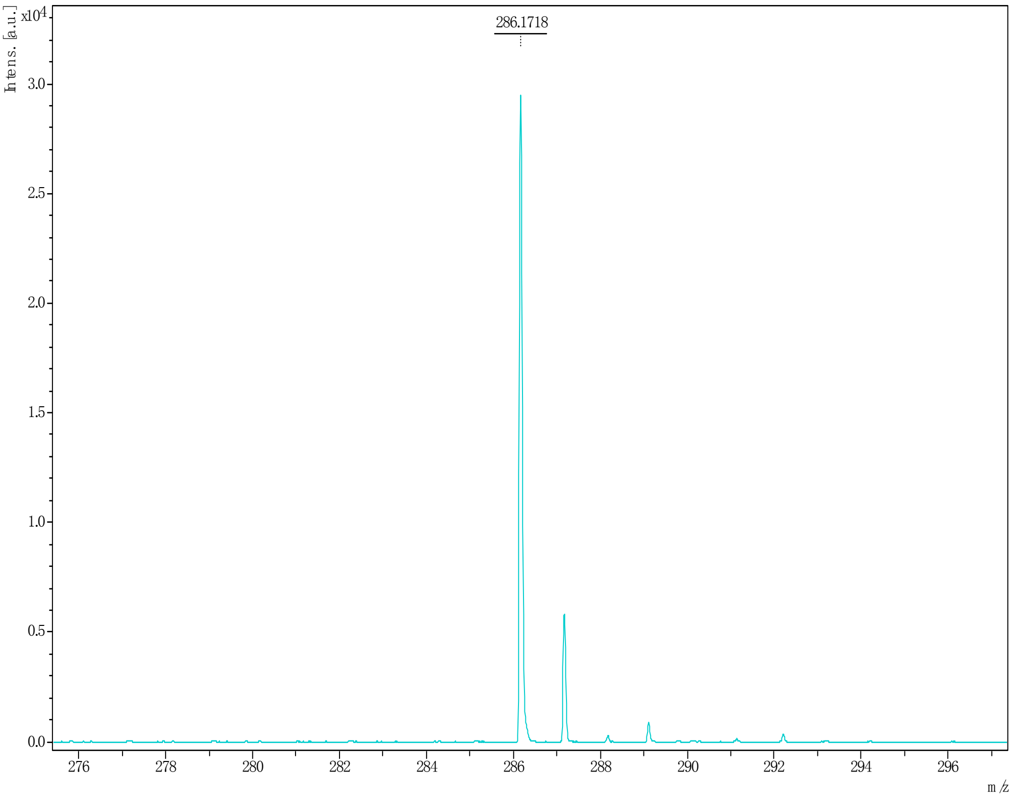

Figure S5

4-methoxy-4'-(1-(4-methoxyphenyl)ethyl)-1,1'-biphenyl (3c)

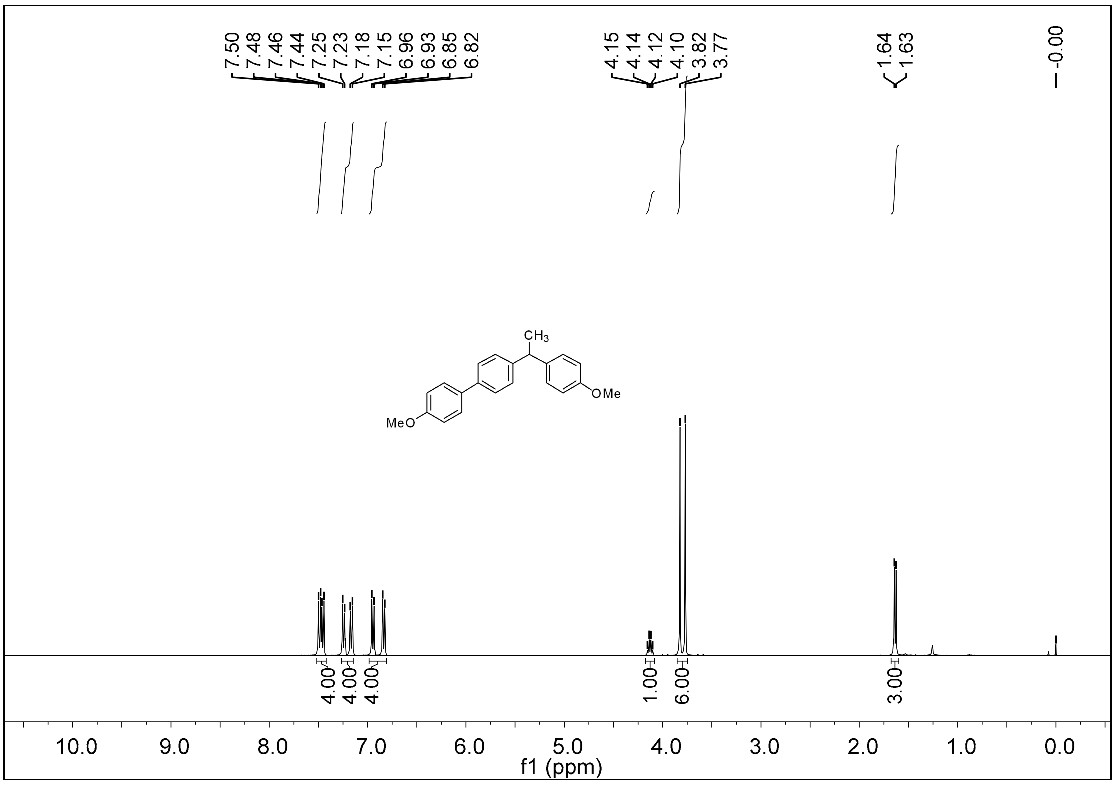

Figure S6

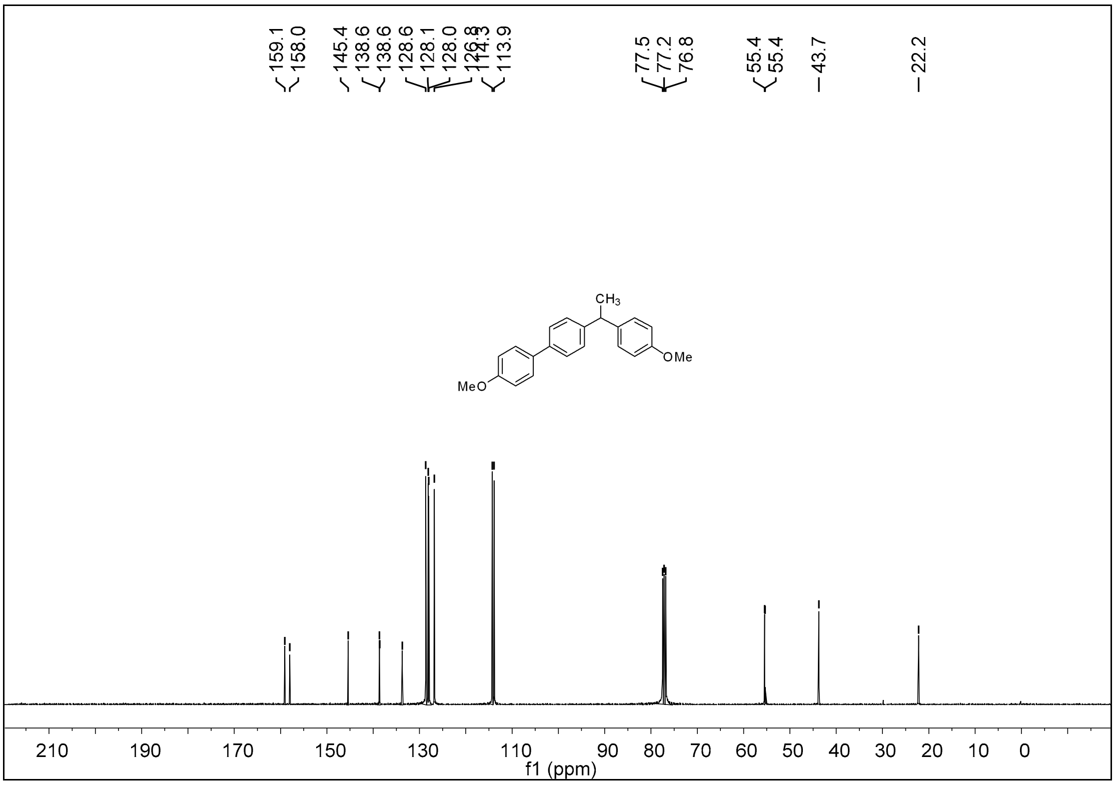

Figure S7

Elemental Composition Report **Page 1**

Tolerance = 1.0 mDa / DBE: min = -1.5, max = 50.0
Element prediction: Off

Monoisotopic Mass, Odd and Even Electron Ions
25 formula(e) evaluated with 1 results within limits (up to 70 best isotopic matches for each mass)
Elements Used:
C: 0-50 H: 0-100 O: 0-5

GCT Premier ZJU 26-Jun-2015 gn-h21 874 (4.158)
TOF MS EI+ 1.67e+003

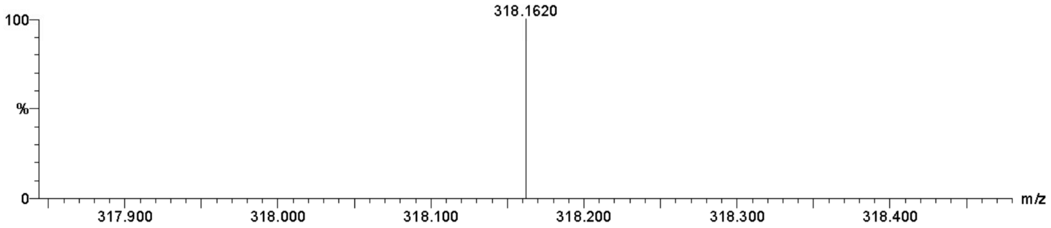

Minimum:				-1.5		
Maximum:		1.0	10.0	50.0		
Mass	Calc. Mass	mDa	PPM	DBE	i-FIT	Formula
318.1620	318.1620	0.0	0.0	12.0	5546847.5	C22 H22 O2

Figure S8

4-fluoro-4'-(1-(4-fluorophenyl)ethyl)-1,1'-biphenyl (3d)

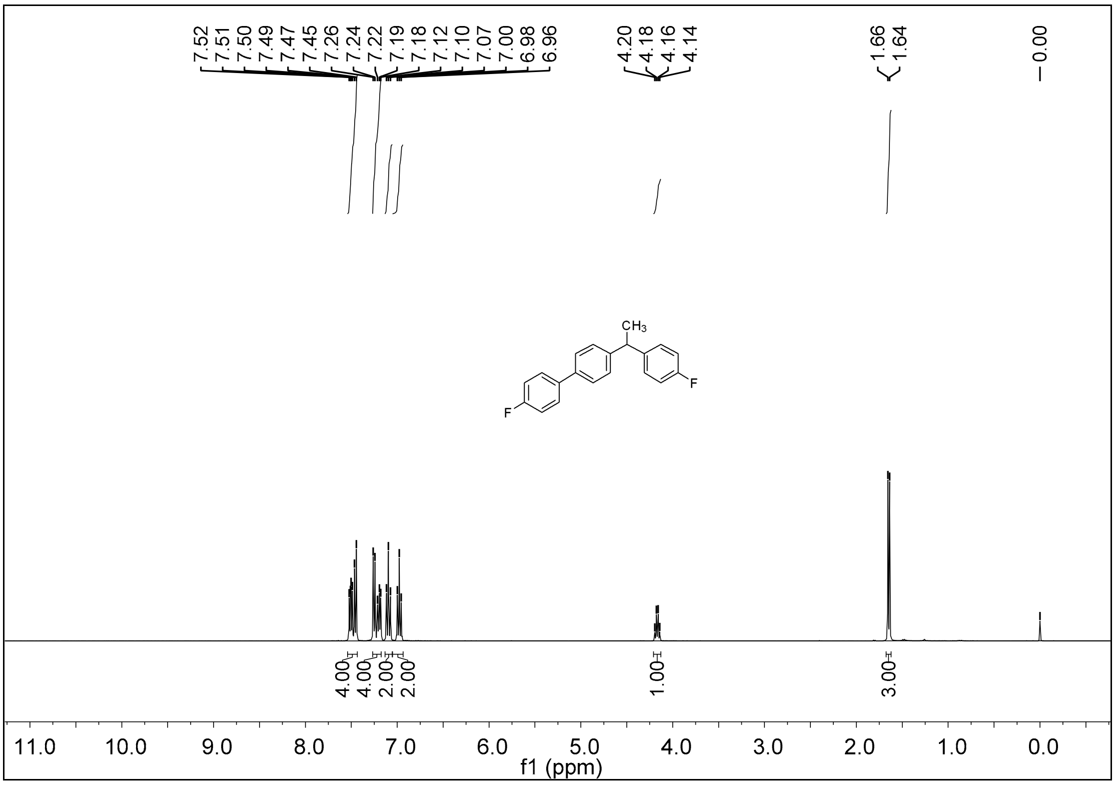

Figure S9

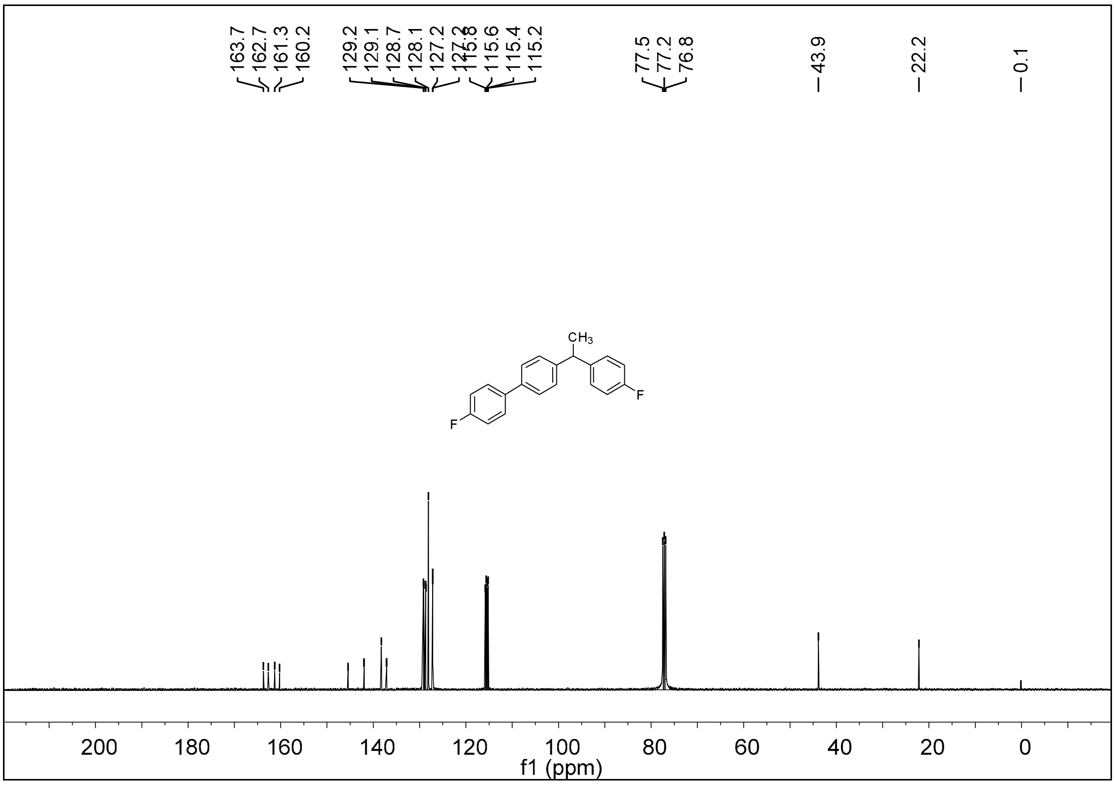

Figure S10

Tolerance = 1.0 mDa / DBE: min = -1.5, max = 50.0
Element prediction: Off

Monoisotopic Mass, Odd and Even Electron Ions
44 formula(e) evaluated with 1 results within limits (up to 70 best isotopic matches for each mass)
Elements Used:
C: 0-50 H: 0-100 O: 0-5 F: 1-2

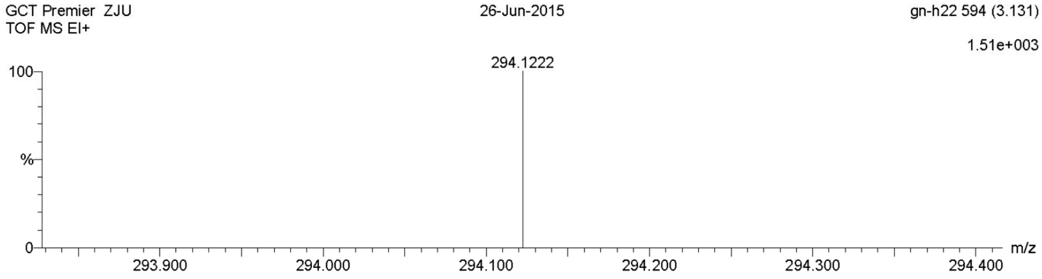

Minimum:				-1.5		
Maximum:		1.0	10.0	50.0		
Mass	Calc. Mass	mDa	PPM	DBE	i-FIT	Formula
294.1222	294.1220	0.2	0.7	12.0	5546765.0	C20 H16 F2

Figure S11

4-(trifluoromethyl)-4'-(1-(trifluoromethyl)phenyl)ethyl)-1,1'-biphenyl (3e)

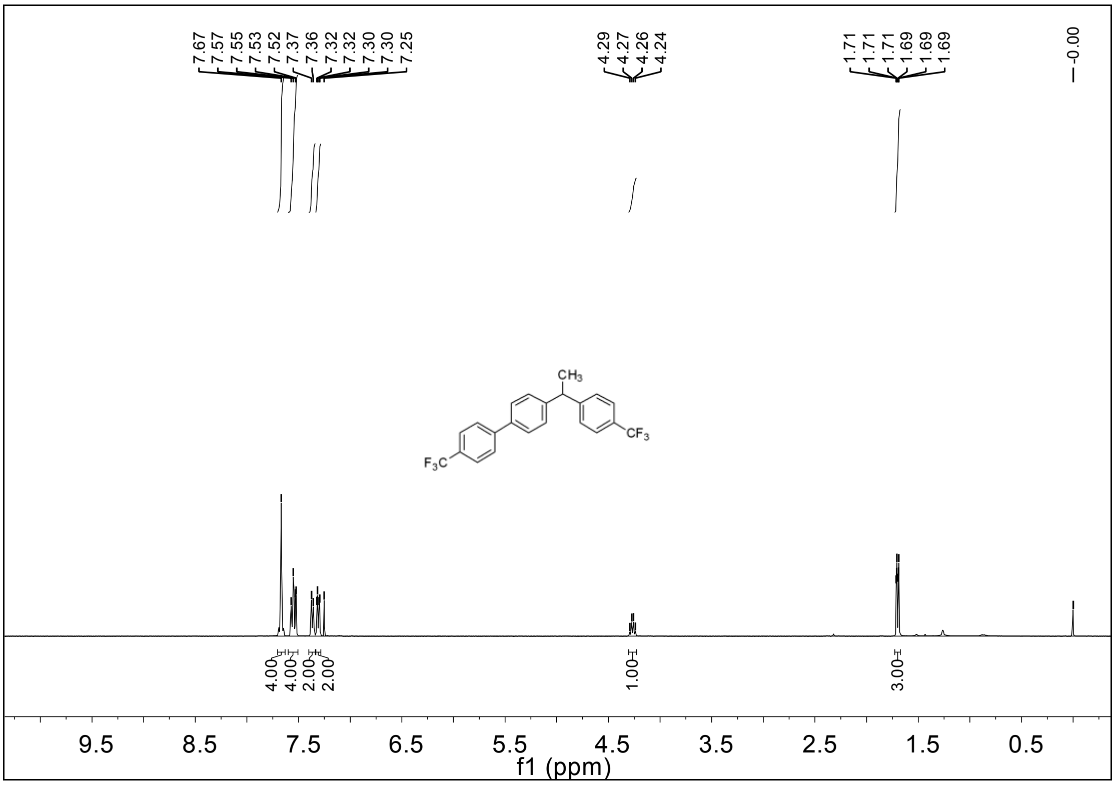

Figure S12

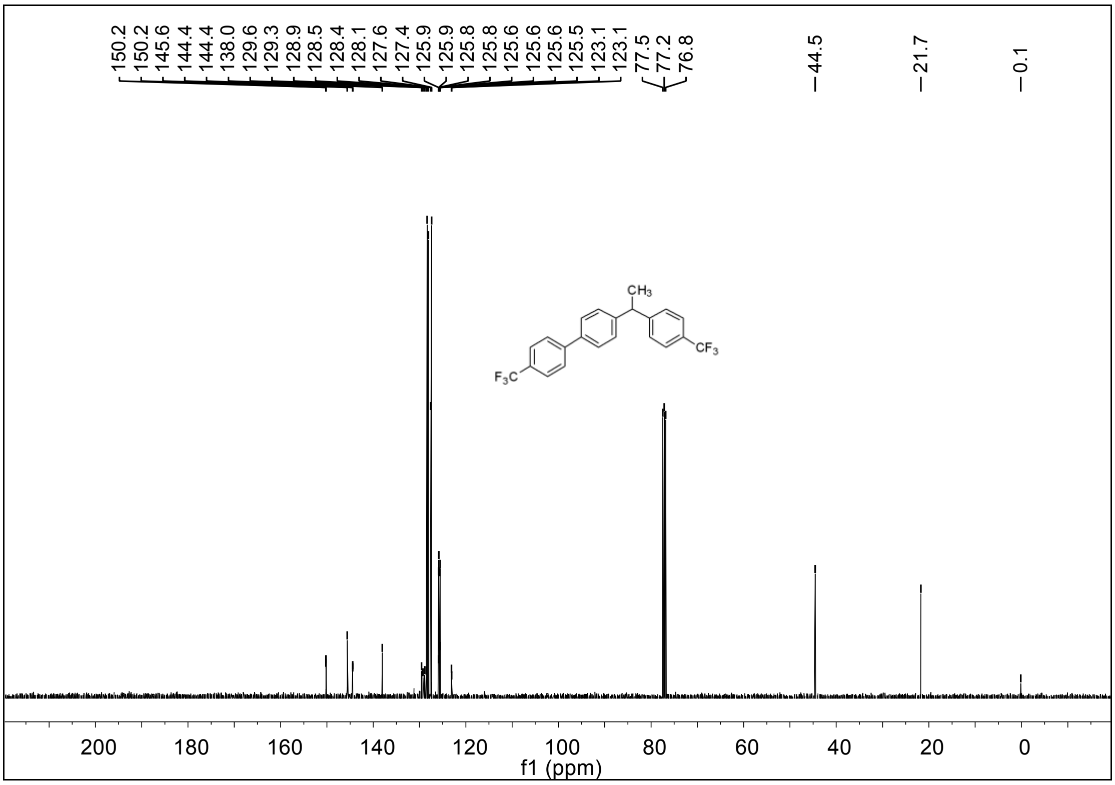

Figure S13

Elemental Composition Report **Page 1**

Tolerance = 1.0 mDa / DBE: min = -1.5, max = 50.0
Element prediction: Off

Monoisotopic Mass, Odd and Even Electron Ions
44 formula(e) evaluated with 1 results within limits (up to 70 best isotopic matches for each mass)
Elements Used:
C: 0-50 H: 0-100 O: 0-5 F: 5-6

GCT Premier ZJU 26-Jun-2015 gn-h23 554 (2.984)
TOF MS EI+ 8.04e+002

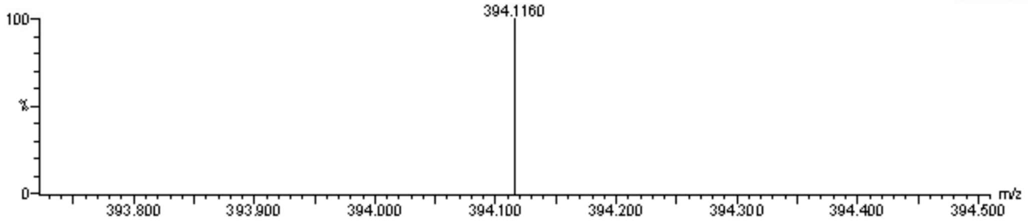

Minimum:				-1.5		
Maximum:		1.0	10.0	50.0		
Mass	Calc. Mass	mDa	PPM	DBE	i-FIT	Formula
394.1160	394.1156	0.4	1.0	12.0	5546415.5	C22 H16 F6

Figure S14

4-propyl-4'-(1-(4-propylphenyl)ethyl)-1,1'-biphenyl (3f)

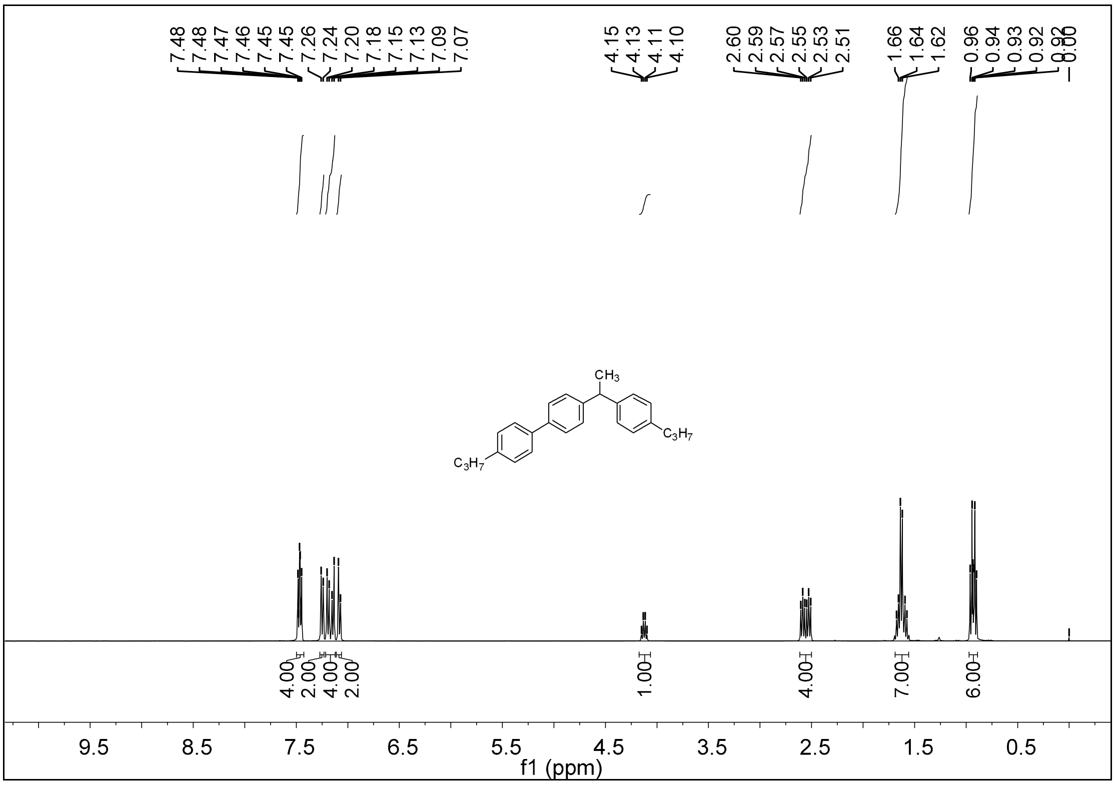

Figure S15

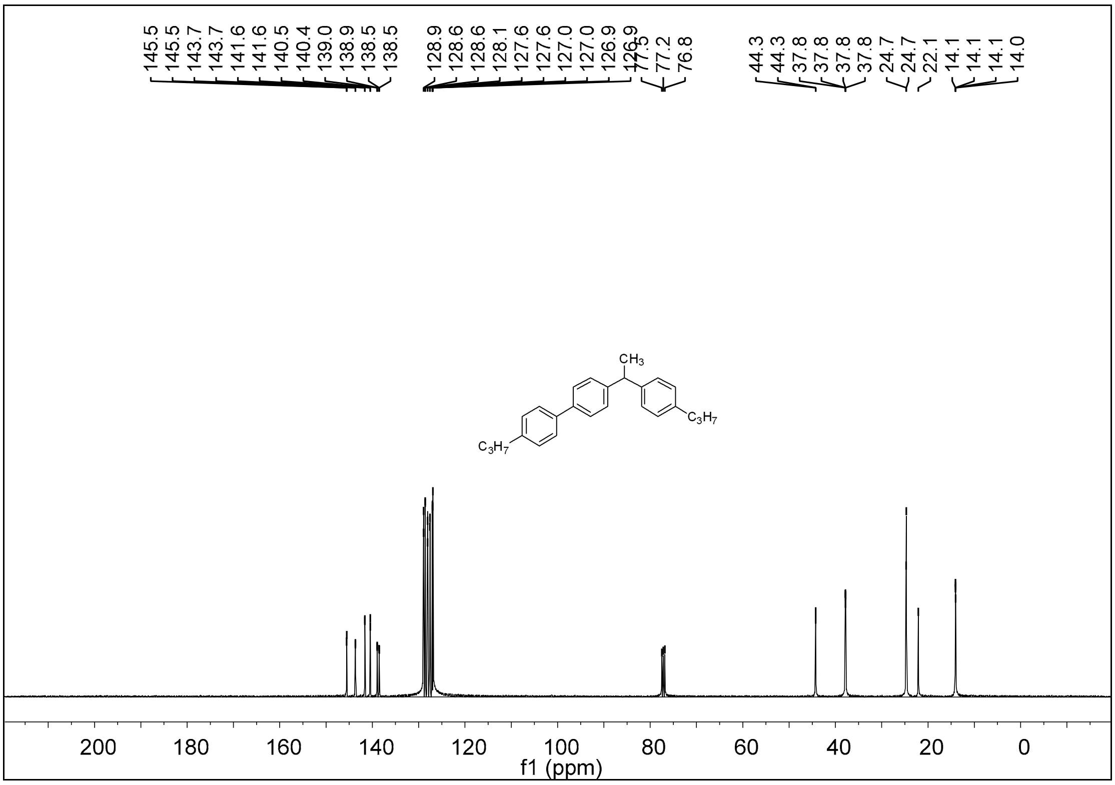

Figure S16

Elemental Composition Report **Page 1**

Tolerance = 1.0 mDa / DBE: min = -1.5, max = 50.0
Element prediction: Off

Monoisotopic Mass, Odd and Even Electron Ions
27 formula(e) evaluated with 1 results within limits (up to 70 best isotopic matches for each mass)
Elements Used:
C: 0-50 H: 0-100 O: 0-5
GCT Premier ZJU 26-Jun-2015 gn-h24785 (3.831)
TOF MS EI+

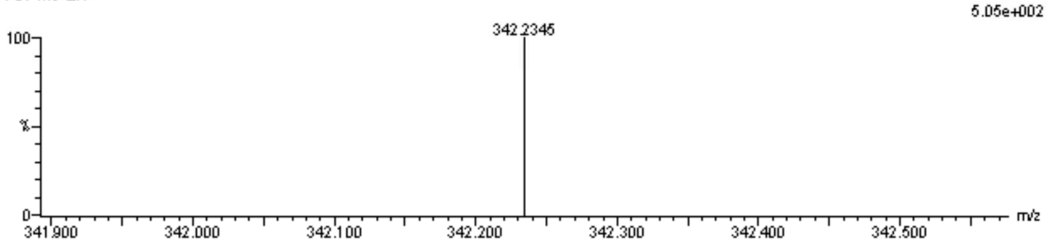

Minimum:				-1.5		
Maximum:		1.0	10.0	50.0		
Mass	Calc. Mass	mDa	PPM	DBE	i-FIT	Formula
342.2345	342.2348	-0.3	-0.9	12.0	5546268.5	C26 H30

Figure S17

3-methyl-4'-(1-(m-tolyl)ethyl)-1,1'-biphenyl (3g)

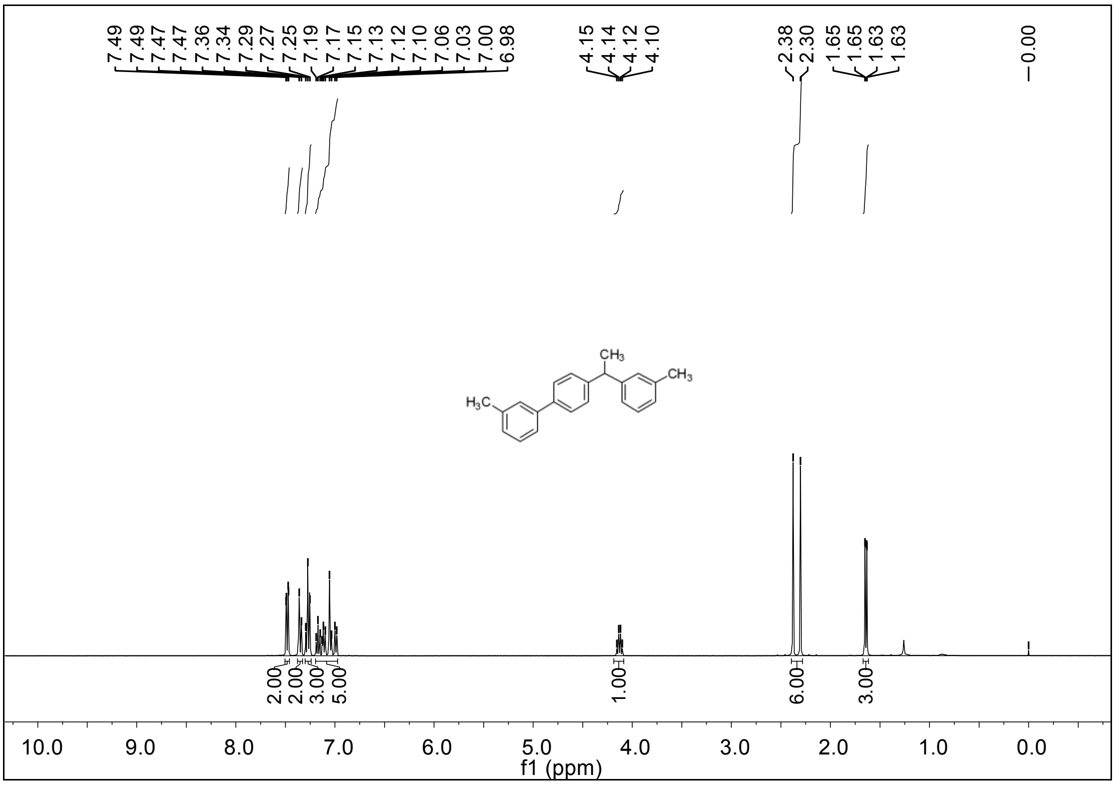

Figure S18

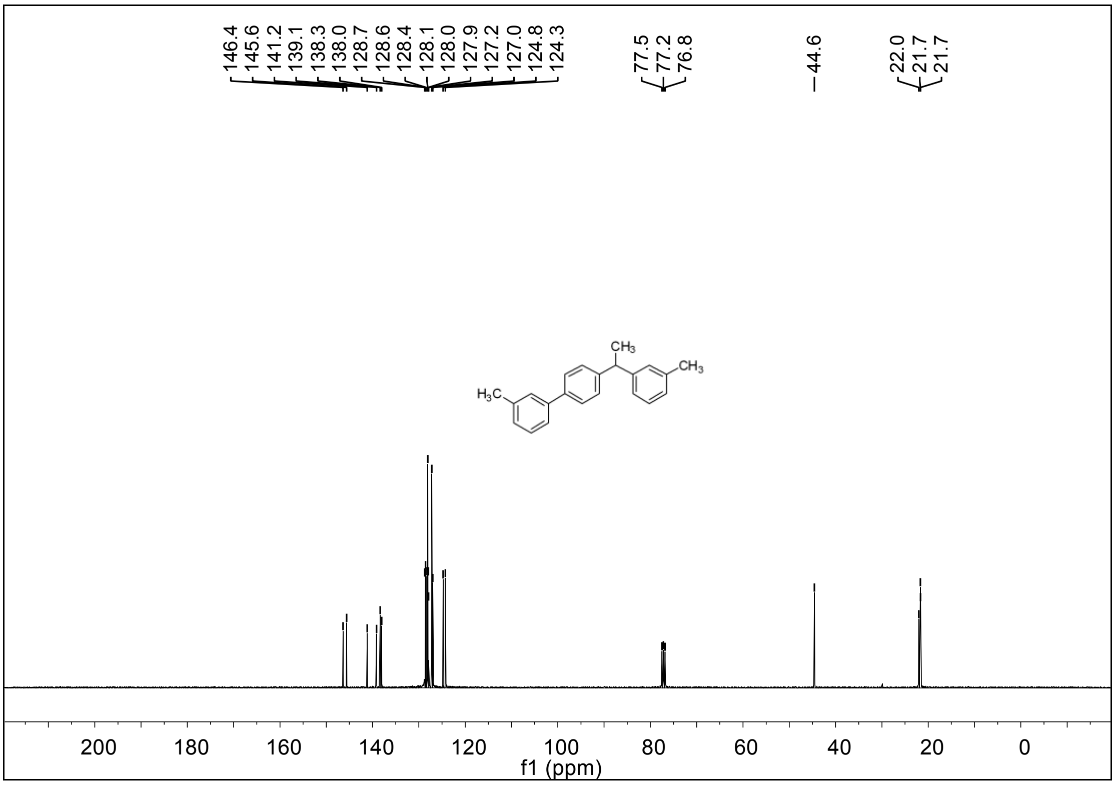

Figure S19

Elemental Composition Report **Page 1**

Tolerance = 1.0 mDa / DBE: min = -1.5, max = 50.0
Element prediction: Off

Monoisotopic Mass, Odd and Even Electron Ions
23 formula(e) evaluated with 1 results within limits (up to 70 best isotopic matches for each mass)
Elements Used:
C: 0-50 H: 0-100 O: 0-5

GCT Premier ZJU 26-Jun-2015 gn-h25 625 (3.245)
TOF MS EI+ 1.00e+003

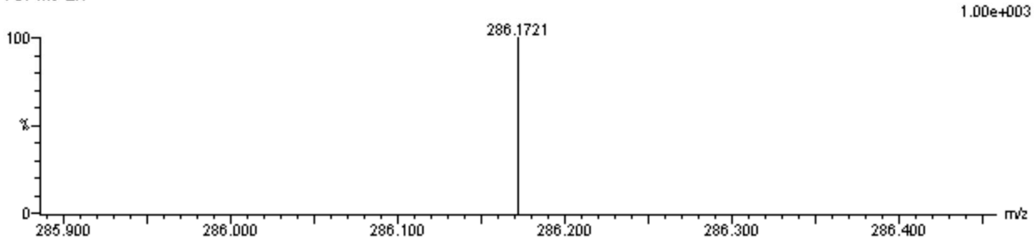

Minimum:				-1.5		
Maximum:		1.0	10.0	50.0		
Mass	Calc. Mass	mDa	PPM	DBE	i-FIT	Formula
286.1721	286.1722	-0.1	-0.3	12.0	5546513.5	C22 H22

Figure S20

3-methoxy-4'-(1-(3-methoxyphenyl)-1,1'-biphenyl (3h)

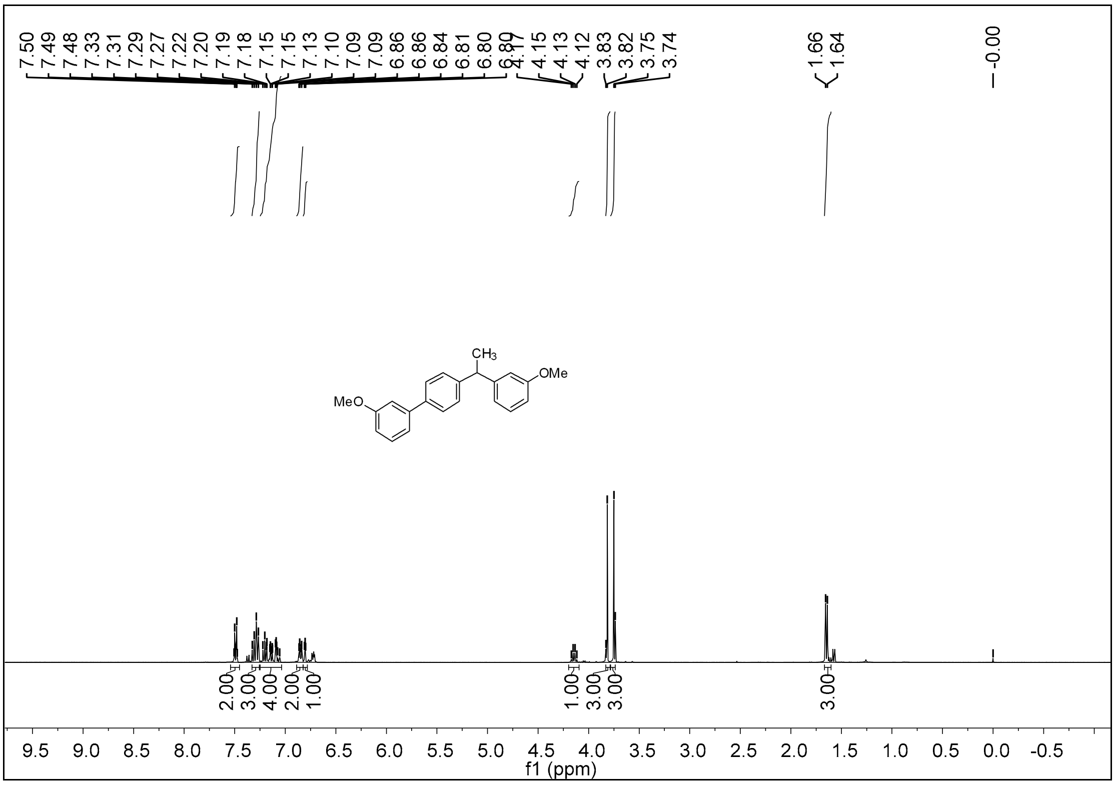

Figure S21

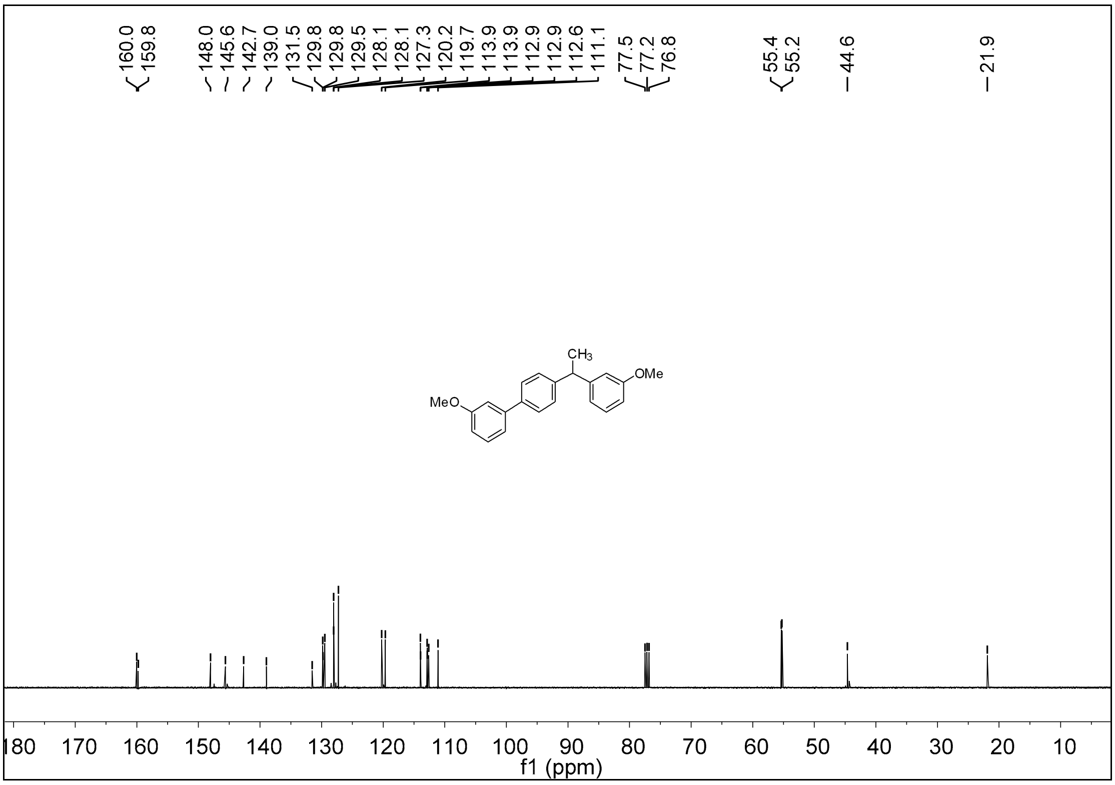

Figure S22

Elemental Composition Report **Page 1**

Tolerance = 1.0 mDa / DBE: min = -1.5, max = 50.0
Element prediction: Off

Monoisotopic Mass, Odd and Even Electron Ions
25 formula(e) evaluated with 1 results within limits (up to 70 best isotopic matches for each mass)
Elements Used:
C: 0-50 H: 0-100 O: 0-5

GCT Premier ZJU 26-Jun-2015 gn-h29 777 (3.802)
TOF MS EI+ 1.55e+003

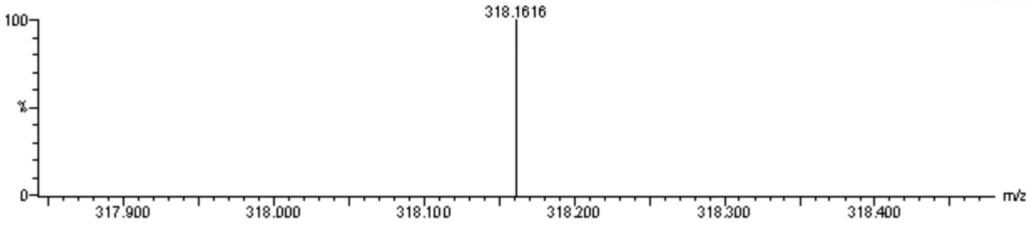

Minimum:				-1.5		
Maximum:		1.0	10.0	50.0		
Mass	Calc. Mass	mDa	PPM	DBE	i-FIT	Formula
318.1616	318.1620	-0.4	-1.3	12.0	5546786.5	C22 H22 O2

Figure S23

3,4,5-trifluoro-4'-(1-(3,4,5-trifluorophenyl)ethyl)-1,1'biphenyl (3i)

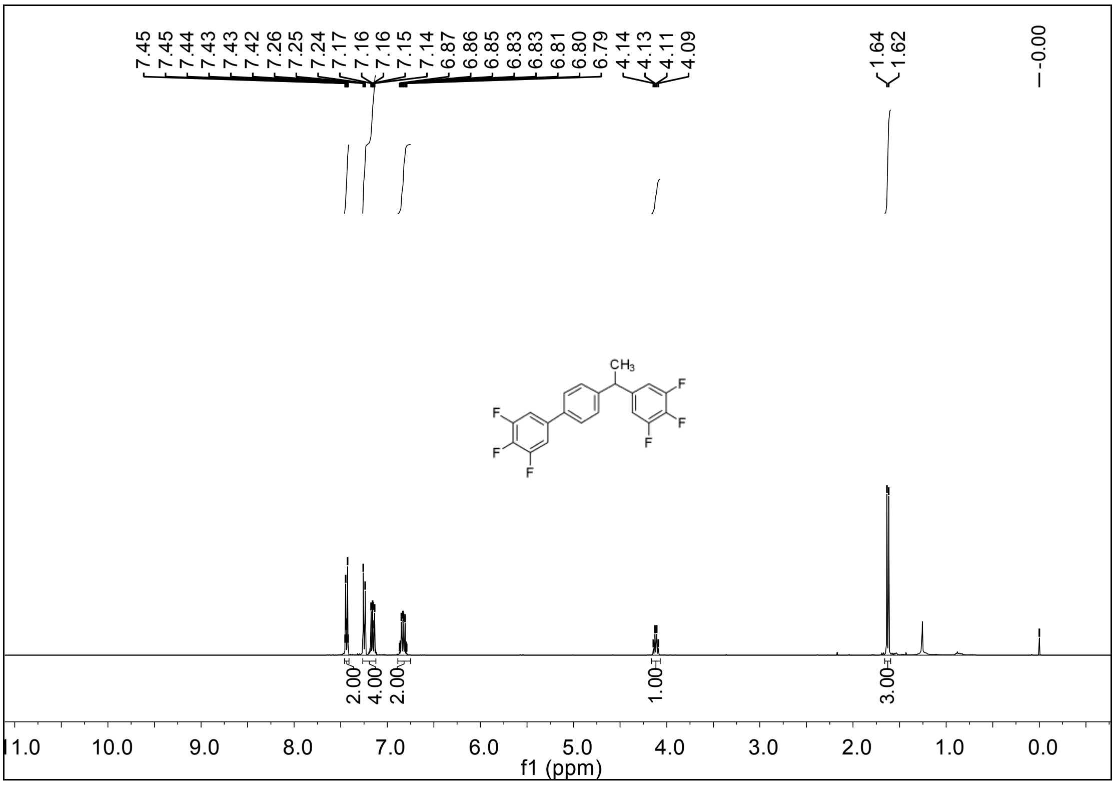

Figure S24

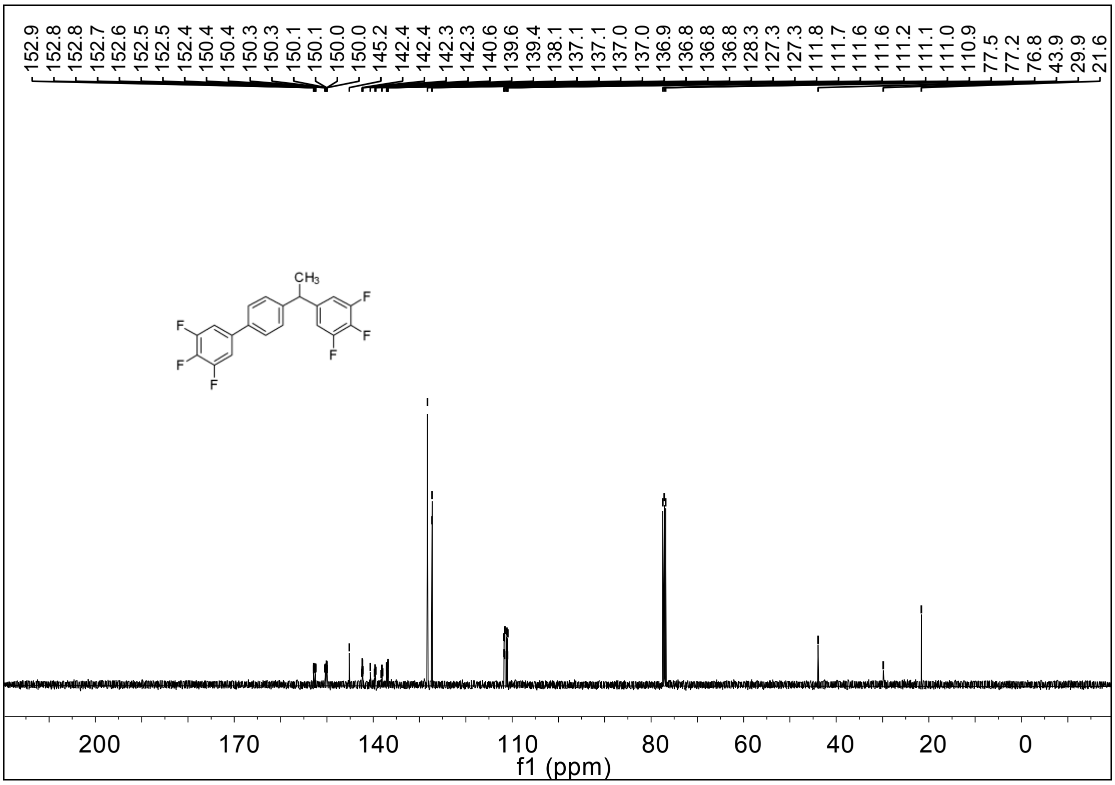

Figure S25

Elemental Composition Report **Page 1**

Tolerance = 1.0 mDa / DBE: min = -1.5, max = 50.0
Element prediction: Off

Monoisotopic Mass, Odd and Even Electron Ions
40 formula(e) evaluated with 1 results within limits (up to 70 best isotopic matches for each mass)
Elements Used:
C: 0-50 H: 0-100 O: 0-5 F: 5-6

GCT Premier ZJU 26-Jun-2015 gn-h26 567 (3.032)
TOF MS EI+ 7.61e+002

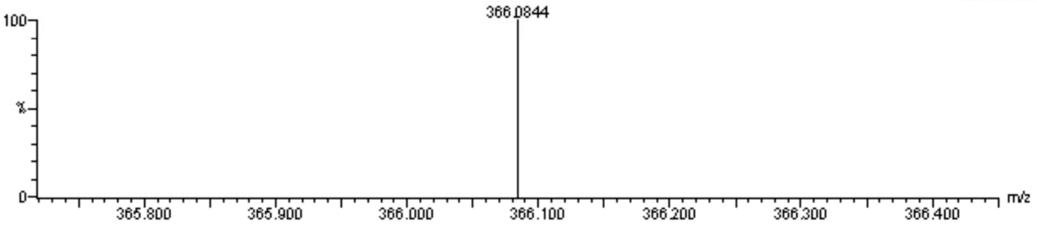

Minimum:				-1.5		
Maximum:		1.0	10.0	50.0		
Mass	Calc. Mass	mDa	PPM	DBE	i-FIT	Formula
366.0844	366.0843	0.1	0.3	12.0	5546391.5	C20 H12 F6

Figure S26

2-methyl-4'-(1-(o-tolyl)ethyl)-1,1'-biphenyl (3j)

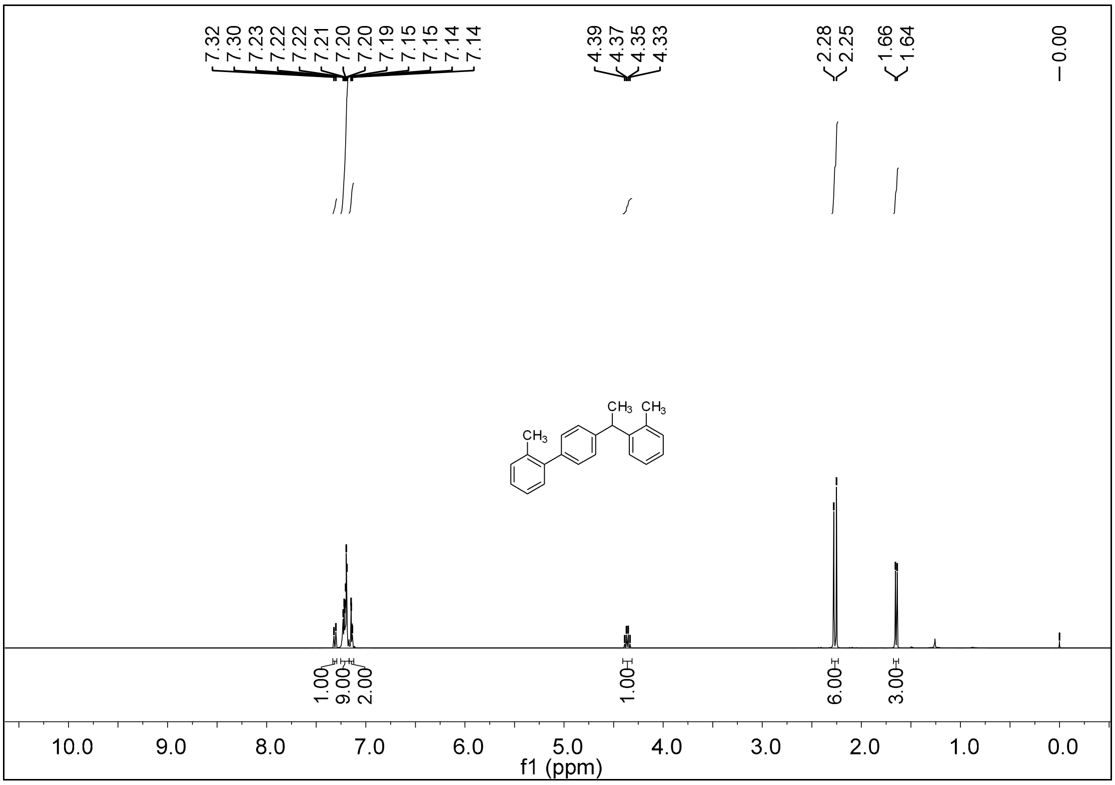

Figure S27

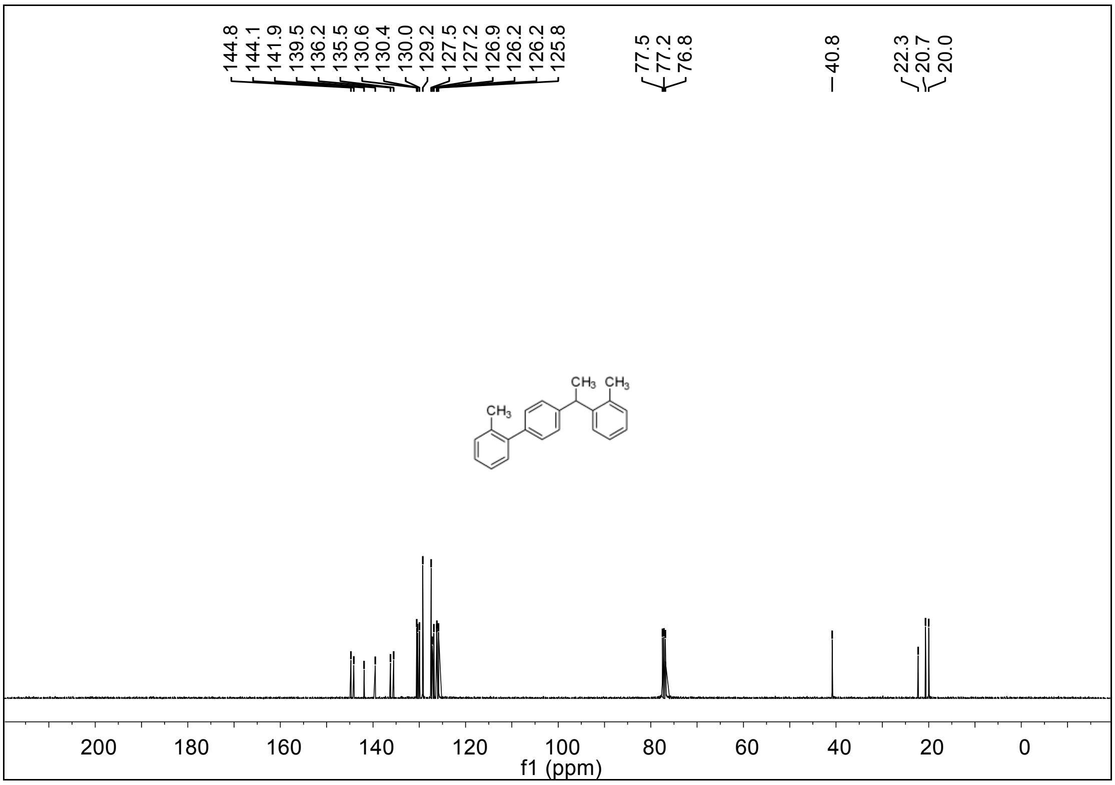

Figure S28

Elemental Composition Report **Page 1**

Tolerance = 1.0 mDa / DBE: min = -1.5, max = 50.0
Element prediction: Off

Monoisotopic Mass, Odd and Even Electron Ions
23 formula(e) evaluated with 1 results within limits (up to 70 best isotopic matches for each mass)
Elements Used:
C: 0-50 H: 0-100 O: 0-5
GCT Premier ZJU 26-Jun-2015 gn-h27 464 (2.654)
TOF MS EI+ 2.67e+003

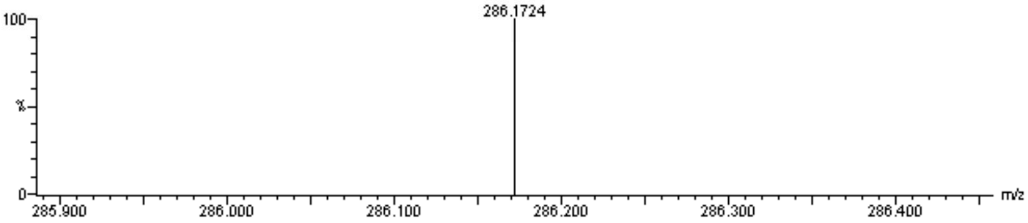

Minimum:				-1.5		
Maximum:		1.0	10.0	50.0		
Mass	Calc. Mass	mDa	PPM	DBE	i-FIT	Formula
286.1724	286.1722	0.2	0.7	12.0	5547347.5	C22 H22

Figure S29

2-(4-(1-(naphthalene-2-yl)ethyl)phenyl)naphthalene (3k)

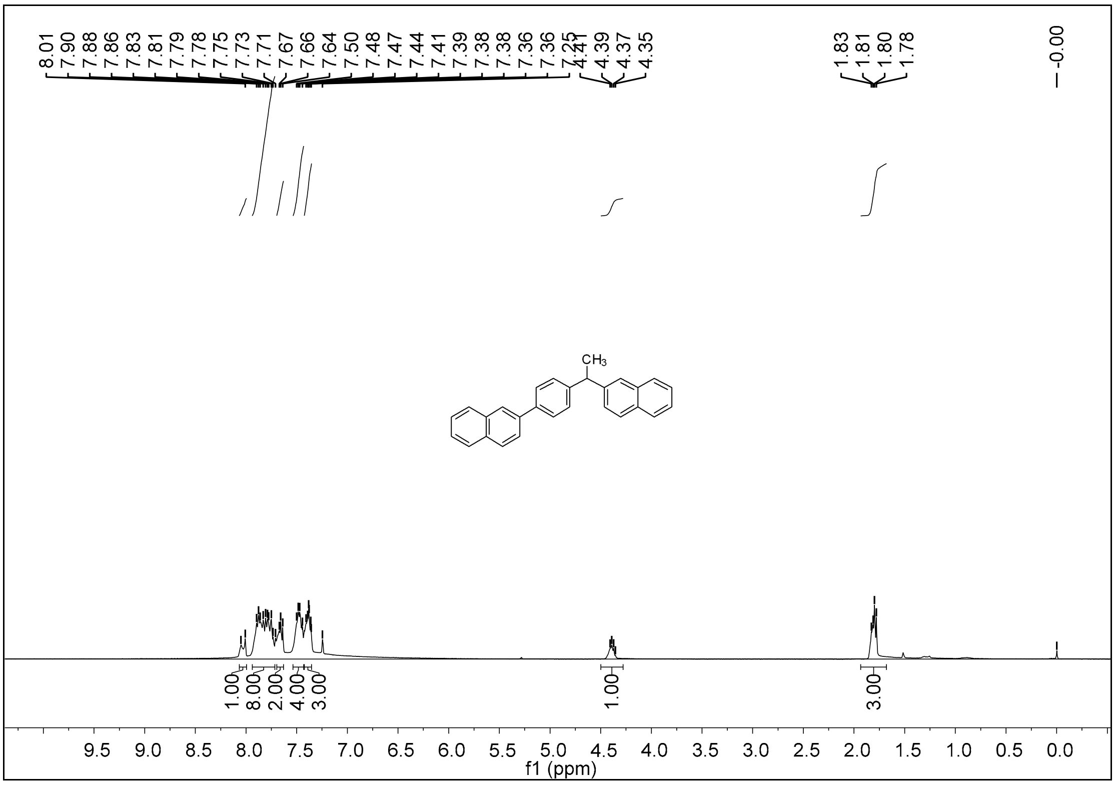

Figure S30

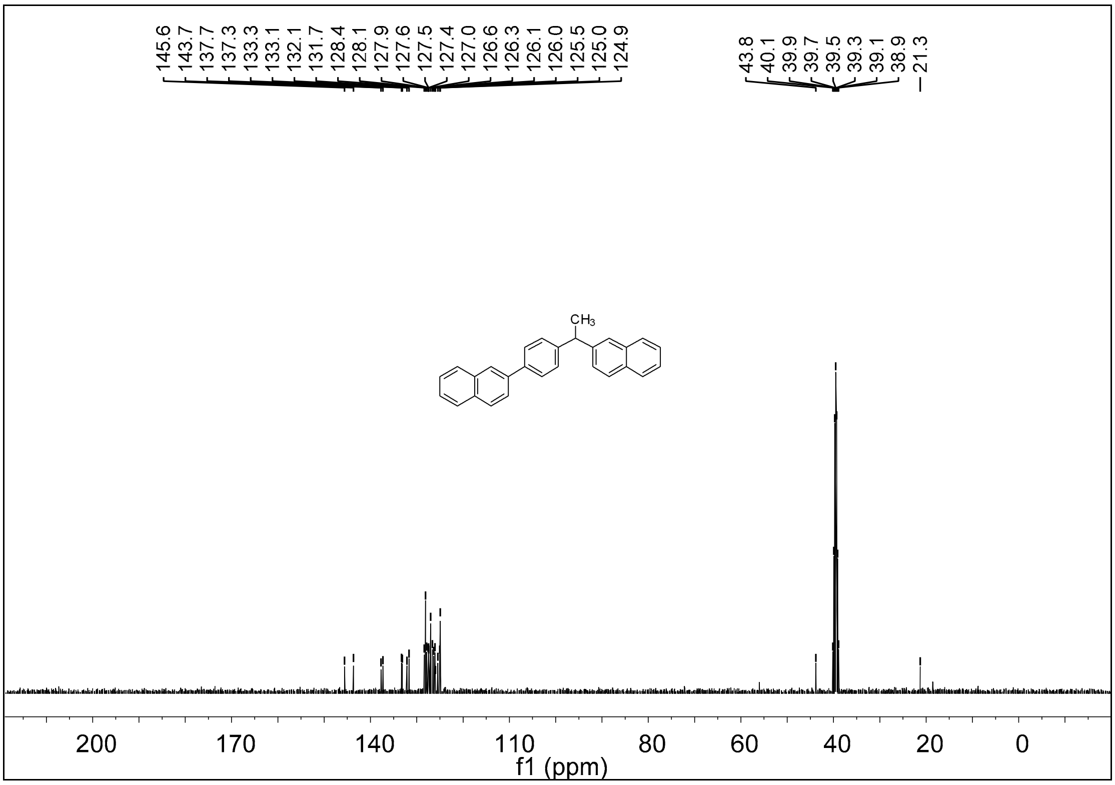

Figure S31

Elemental Composition Report Page 1

Tolerance = 1.0 mDa / DBE: min = -1.5, max = 50.0
Element prediction: Off

Monoisotopic Mass, Odd and Even Electron Ions
28 formula(e) evaluated with 1 results within limits (up to 70 best isotopic matches for each mass)
Elements Used:
C: 0-50 H: 0-100 O: 0-5
GCT Premier ZJU 26-Jun-2015 gn-h30 1027 (4.719)
TOF MS EI+ 1.13e+003

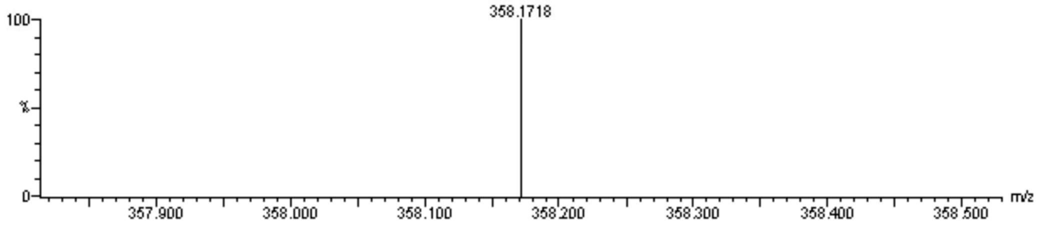

Minimum:				-1.5		
Maximum:		1.0	10.0	50.0		
Mass	Calc. Mass	mDa	PPM	DBE	i-FIT	Formula
358.1718	358.1722	-0.4	-1.1	18.0	5546580.0	C28 H22

Figure S32

3-methyl-3-(1-(m-tolyl)ethyl-1,1-biphenyl (3l)

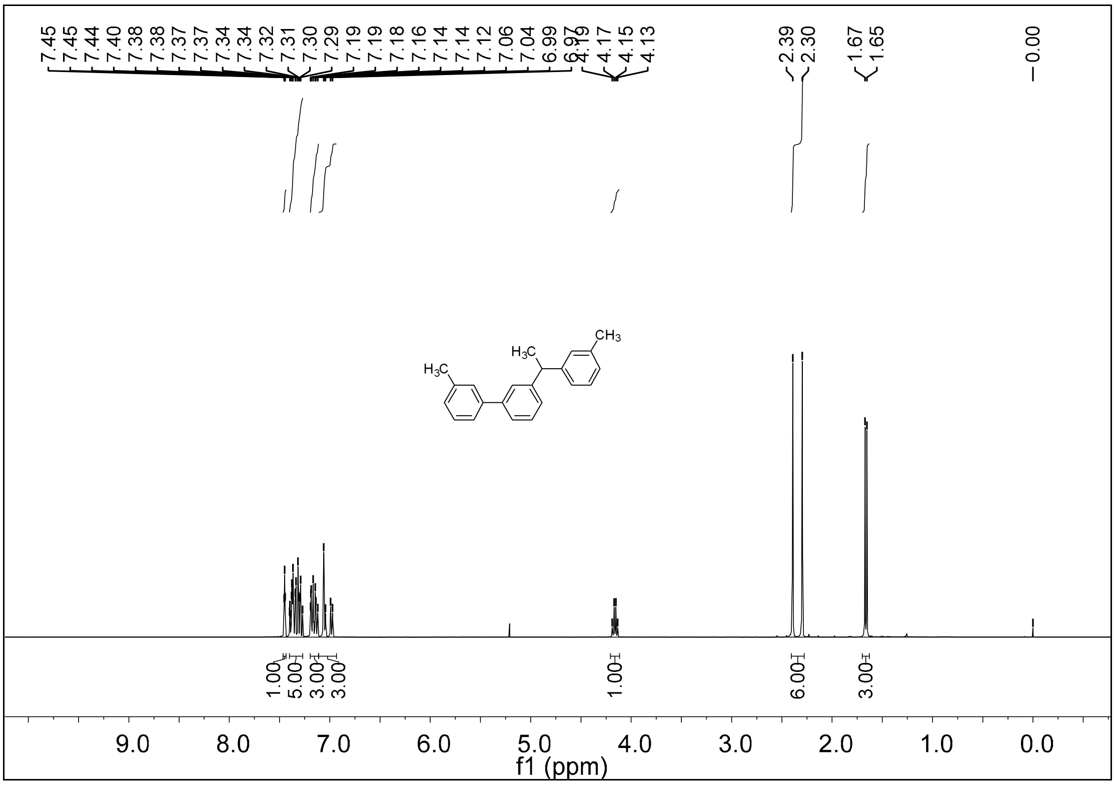

Figure S33

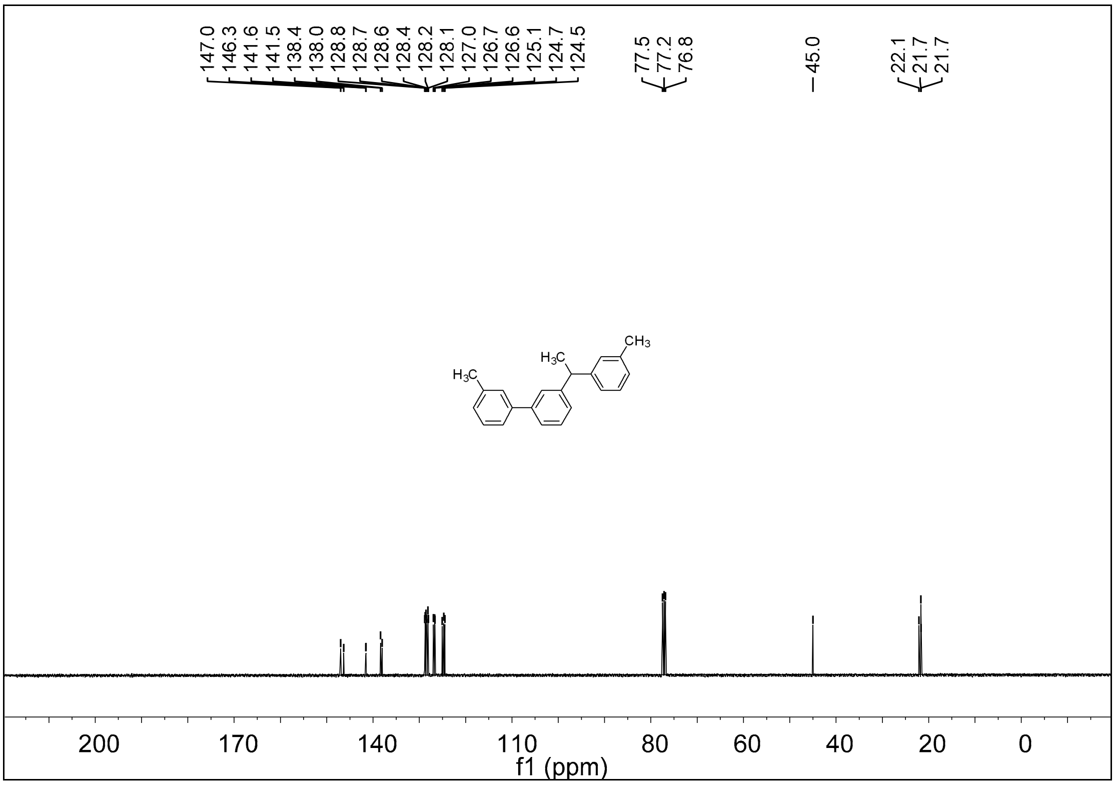

Figure S34

Elemental Composition Report **Page 1**

Tolerance = 0.7 mDa / DBE: min = -1.5, max = 50.0
Element prediction: Off

Monoisotopic Mass, Odd and Even Electron Ions
23 formula(e) evaluated with 1 results within limits (up to 70 best isotopic matches for each mass)
Elements Used:
C: 0-50 H: 0-100 O: 0-5

GCT Premier ZJU 26-Jun-2015 gn-h54471 (2.680)
TOF MS EI+ 1.01e+003

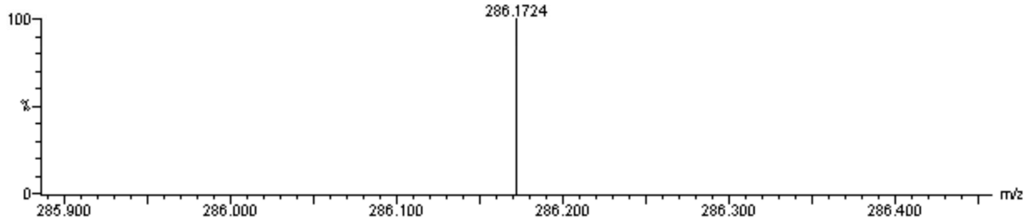

Minimum:				-1.5		
Maximum:		0.7	10.0	50.0		
Mass	Calc. Mass	mDa	PPM	DBE	i-FIT	Formula
286.1724	286.1722	0.2	0.7	12.0	5546520.0	C22 H22

Figure S35

3-methyl-4′-(3-methylbenzyl)-1,1′-biphenyl (3m)

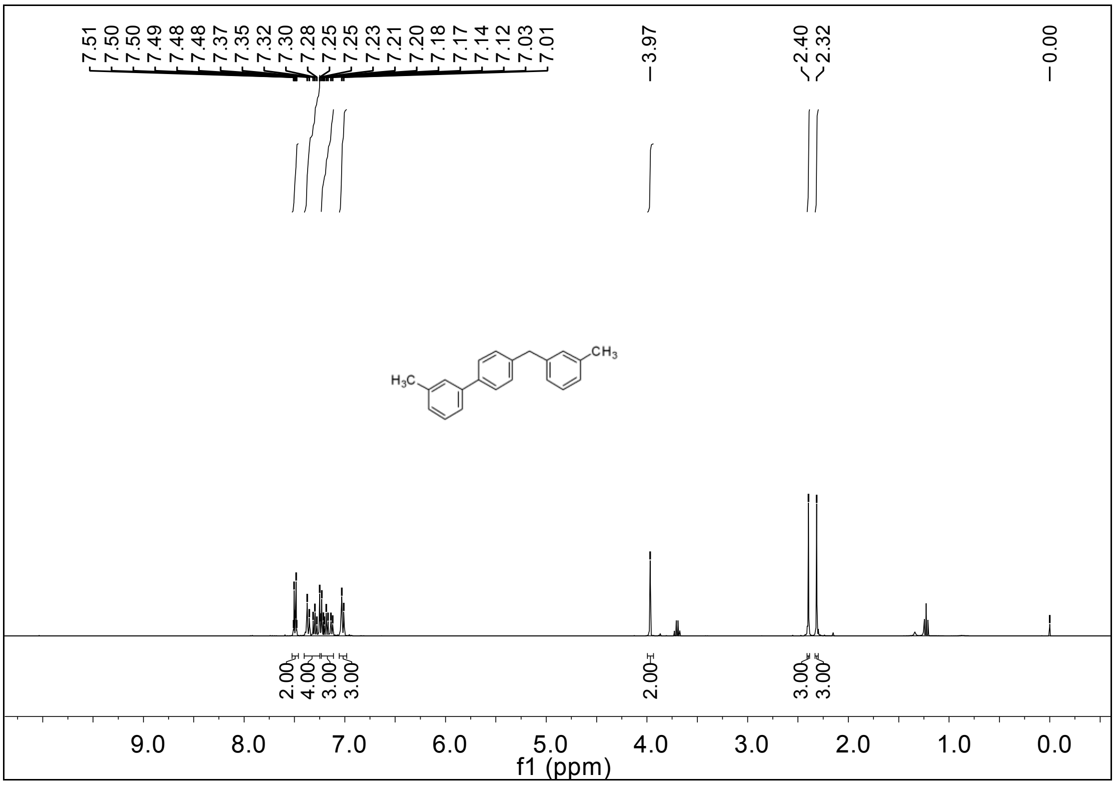

Figure S36

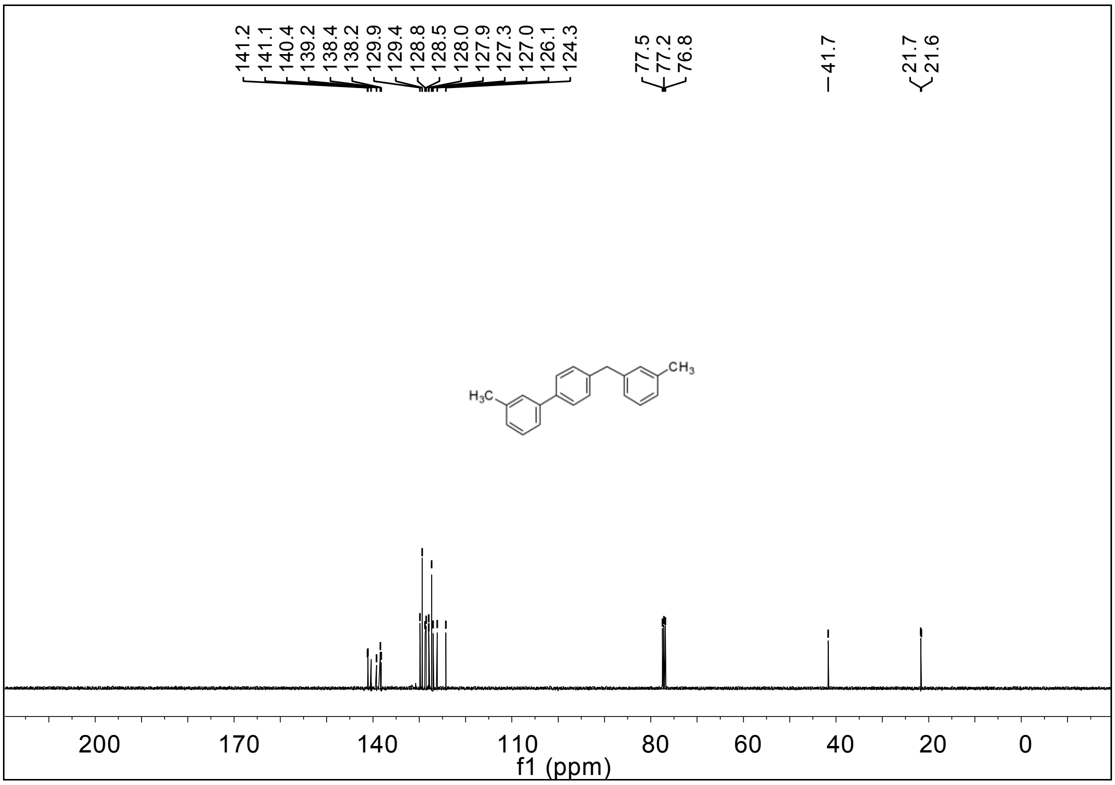

Figure S37

Elemental Composition Report **Page 1**

Tolerance = 1.0 mDa / DBE: min = -1.5, max = 50.0
Element prediction: Off

Monoisotopic Mass, Odd and Even Electron Ions
23 formula(e) evaluated with 1 results within limits (up to 70 best isotopic matches for each mass)
Elements Used:
C: 0-50 H: 0-100 O: 0-5

GCT Premier ZJU 26-Jun-2015 gn-h44620 (3.220)
TOF MS EI+ 9.87e+002

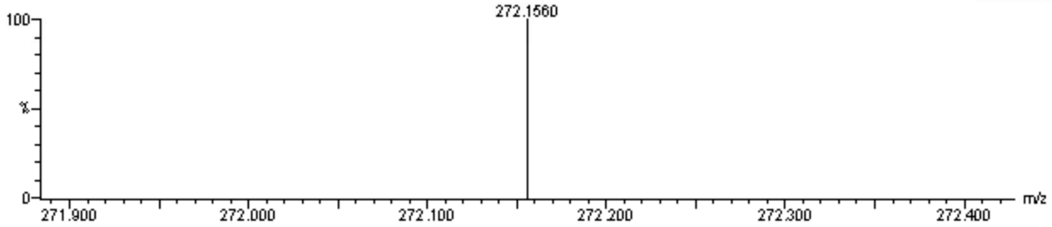

Minimum:				-1.5		
Maximum:		1.0	10.0	50.0		
Mass	Calc. Mass	mDa	PPM	DBE	i-FIT	Formula
272.1560	272.1565	-0.5	-1.8	12.0	5546506.0	C21 H20

Figure S38

3'-methyl-2-(2-methylbenzyl)-1,1'-biphenyl (3n)

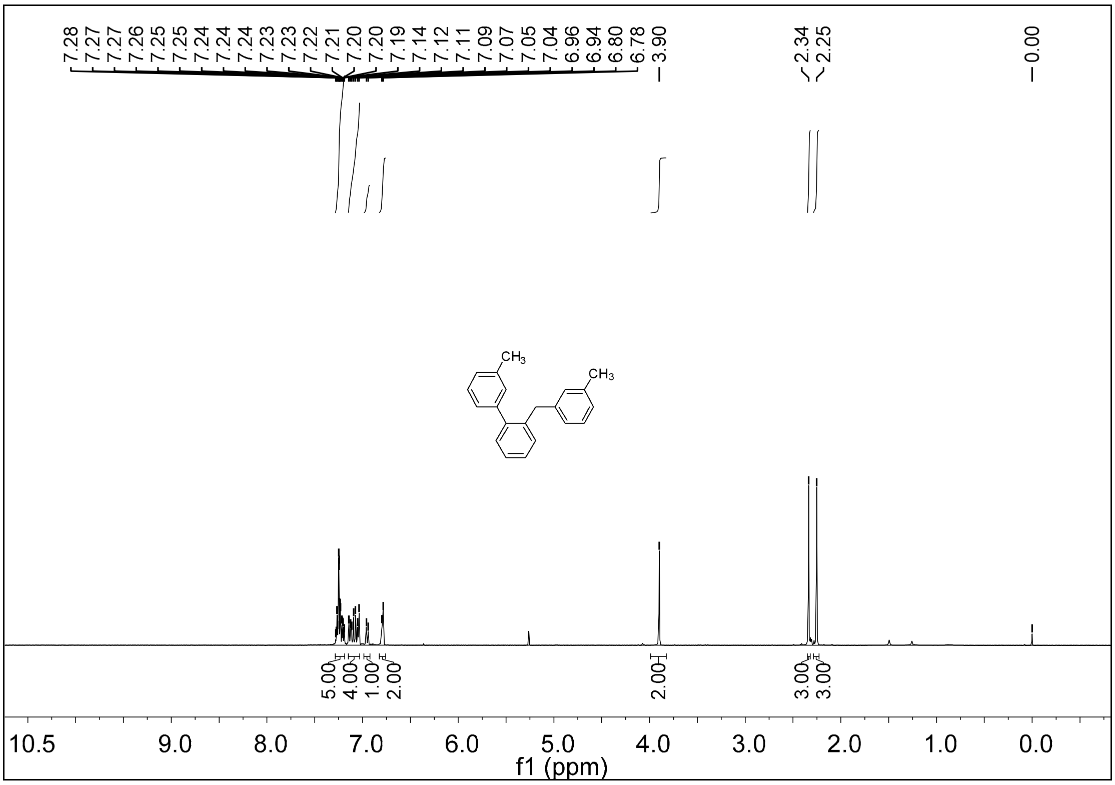

Figure S39

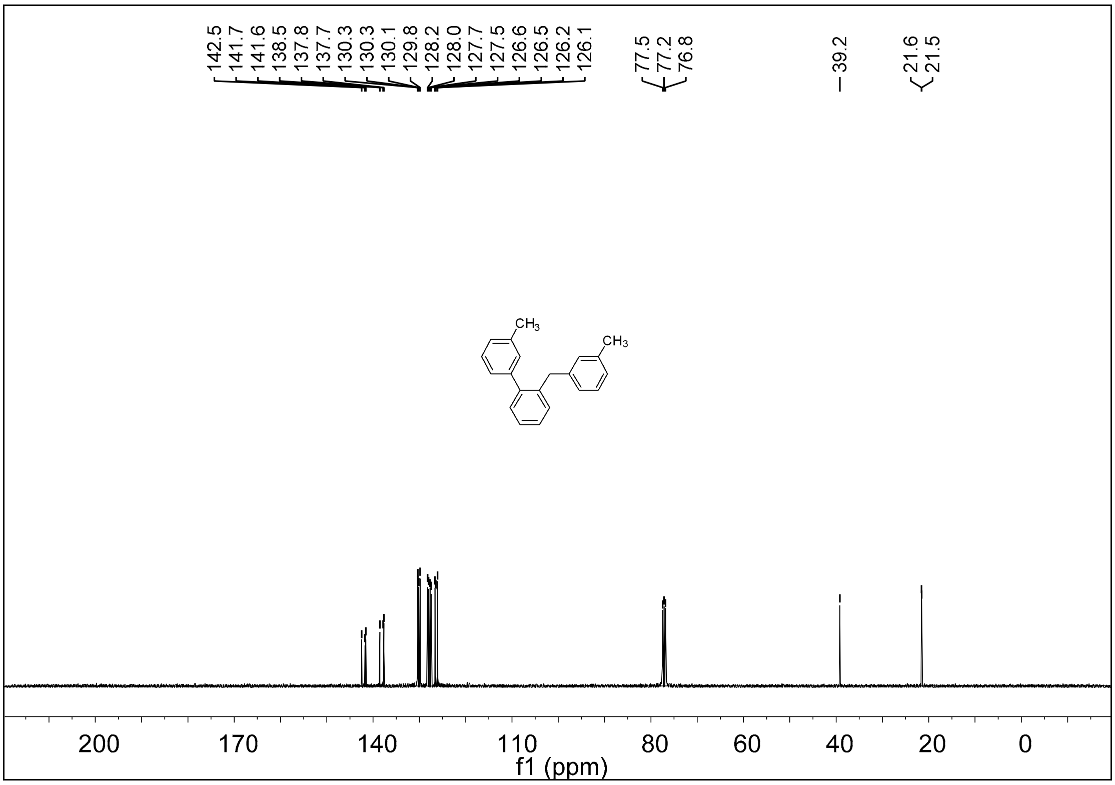

Figure S40

Elemental Composition Report **Page 1**

Tolerance = 0.7 mDa / DBE: min = -1.5, max = 50.0
Element prediction: Off

Monoisotopic Mass, Odd and Even Electron Ions
23 formula(e) evaluated with 1 results within limits (up to 70 best isotopic matches for each mass)
Elements Used:
C: 0-50 H: 0-100 O: 0-5

GCT Premier ZJU 26-Jun-2015 gn-h51 342 (2.207)
TOF MS EI+

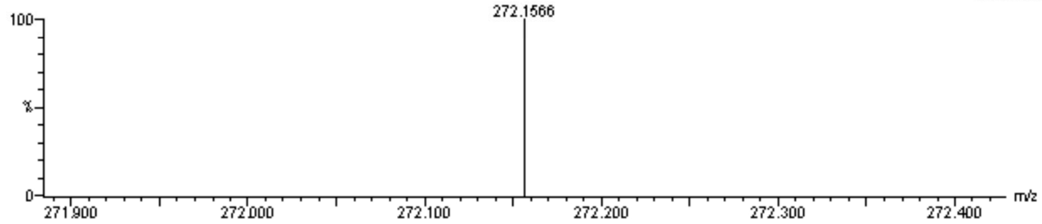

Minimum:				-1.5		
Maximum:		0.7	10.0	50.0		
Mass	Calc. Mass	mDa	PPM	DBE	i-FIT	Formula
272.1566	272.1565	0.1	0.4	12.0	5546994.0	C21 H20

Figure S41

4-methoxy-4'-(1-(p-tolyl)ethyl)-1,1'-biphenyl (3o)

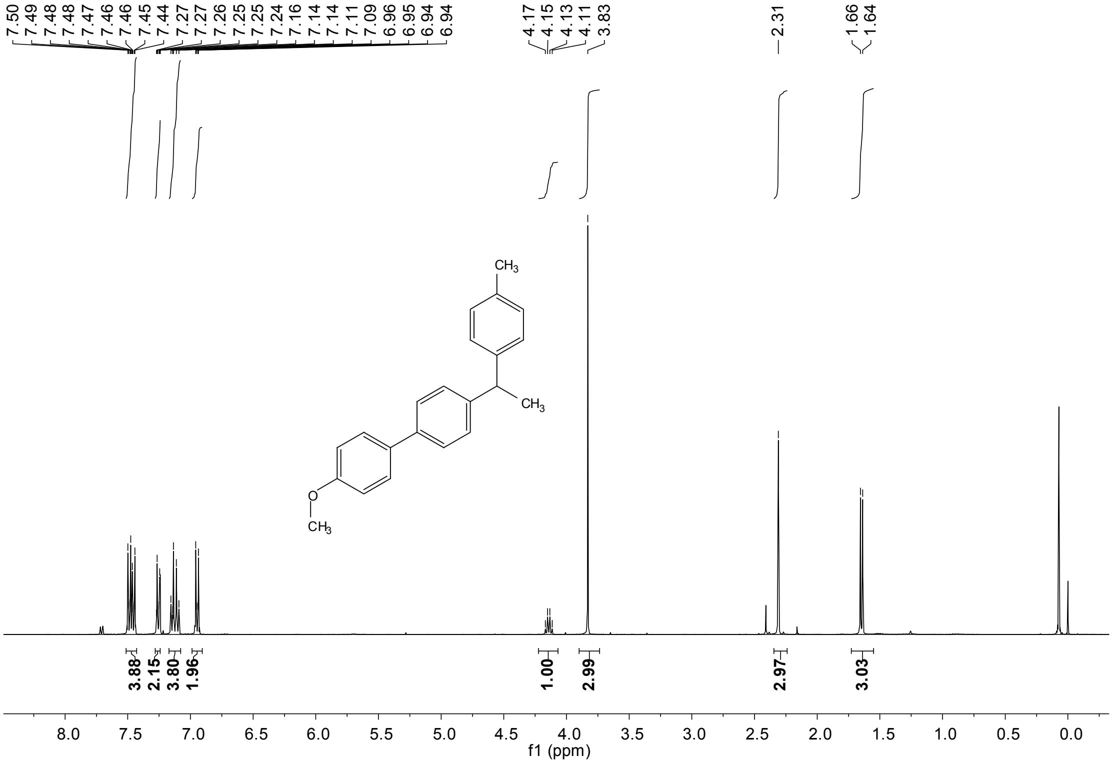

Figure S42

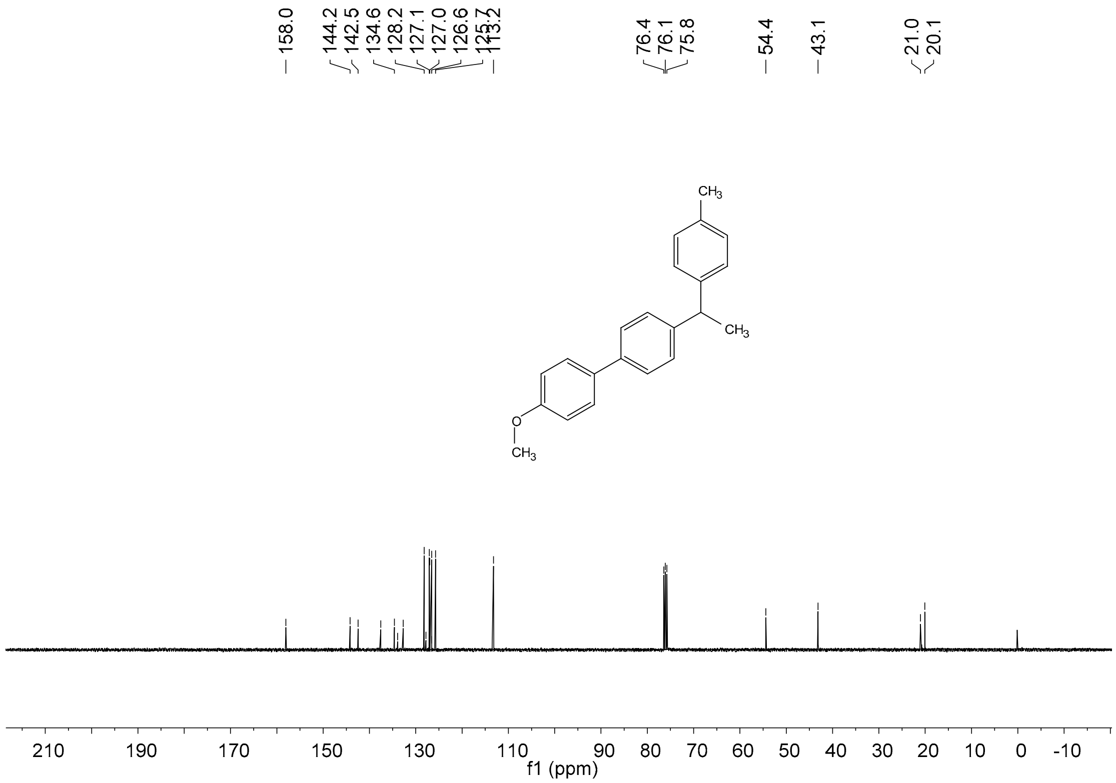

Figure S43

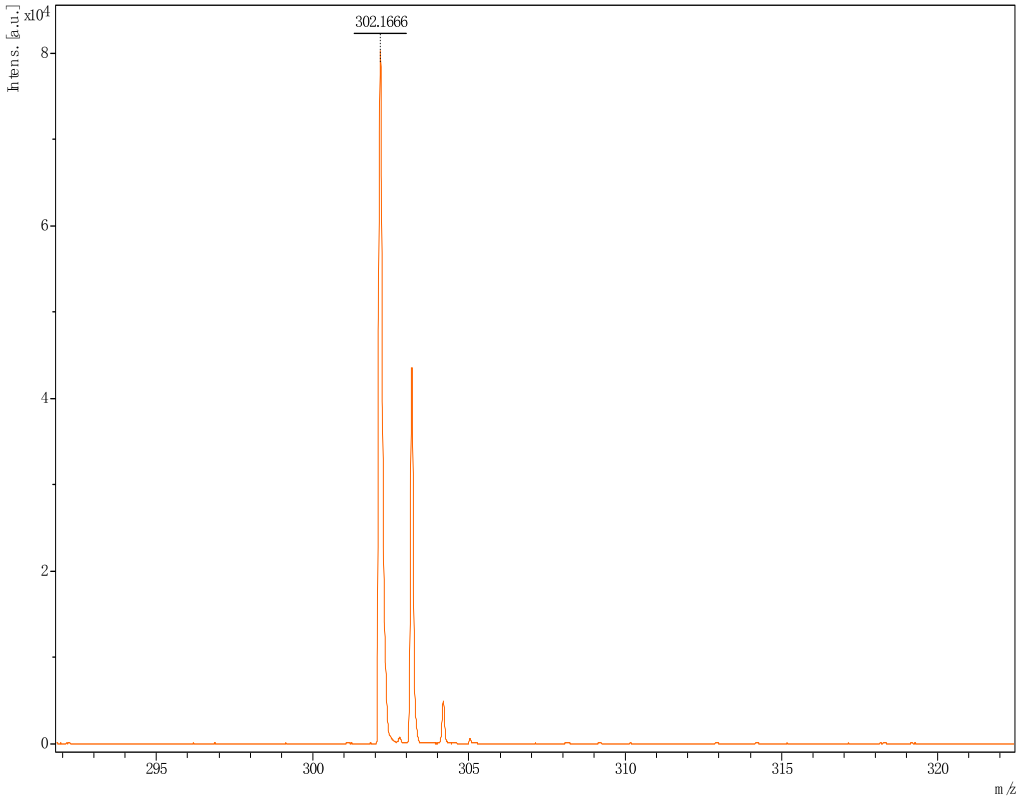

Figure S44

4-propyl-4'-(1-(p-tolyl)ethyl)-1,1'-biphenyl (3p)

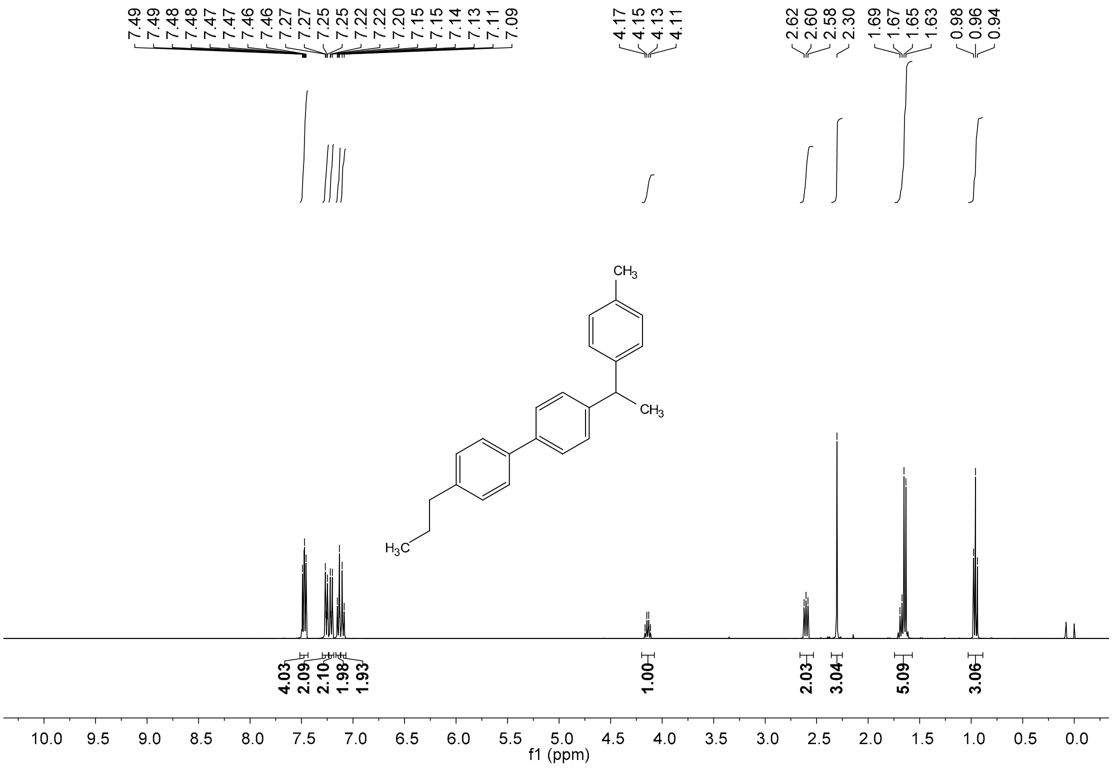

Figure S45

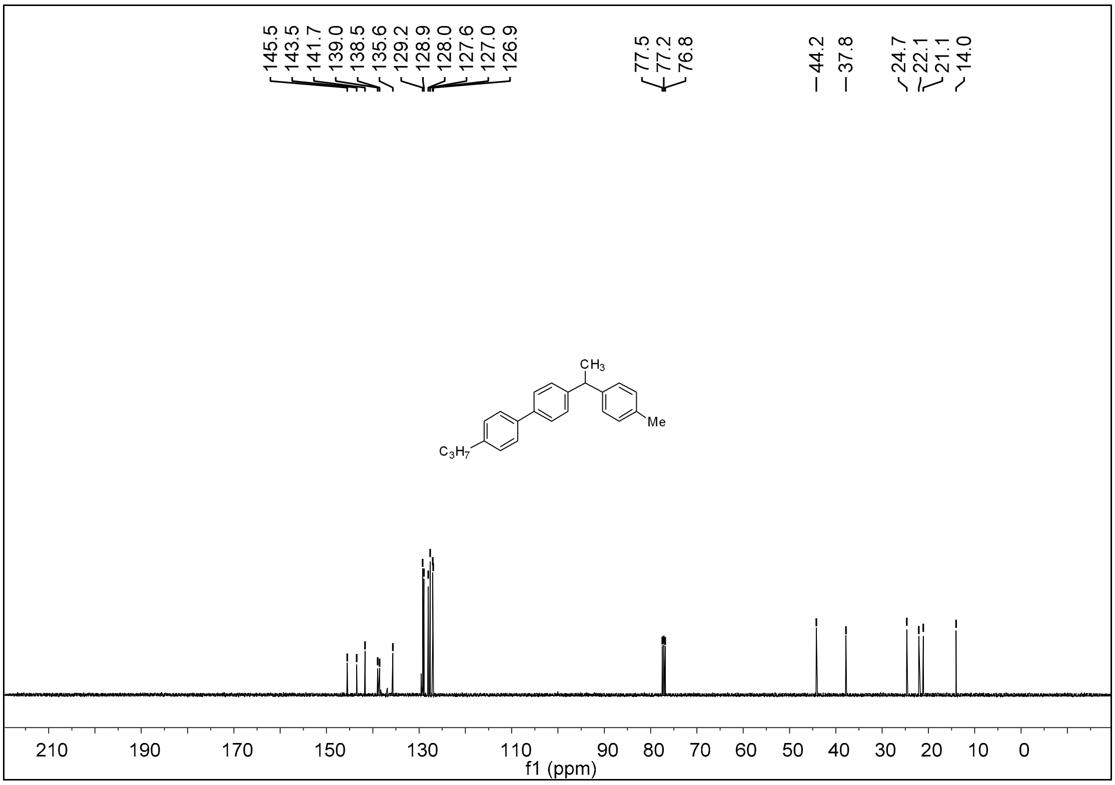

Figure S46

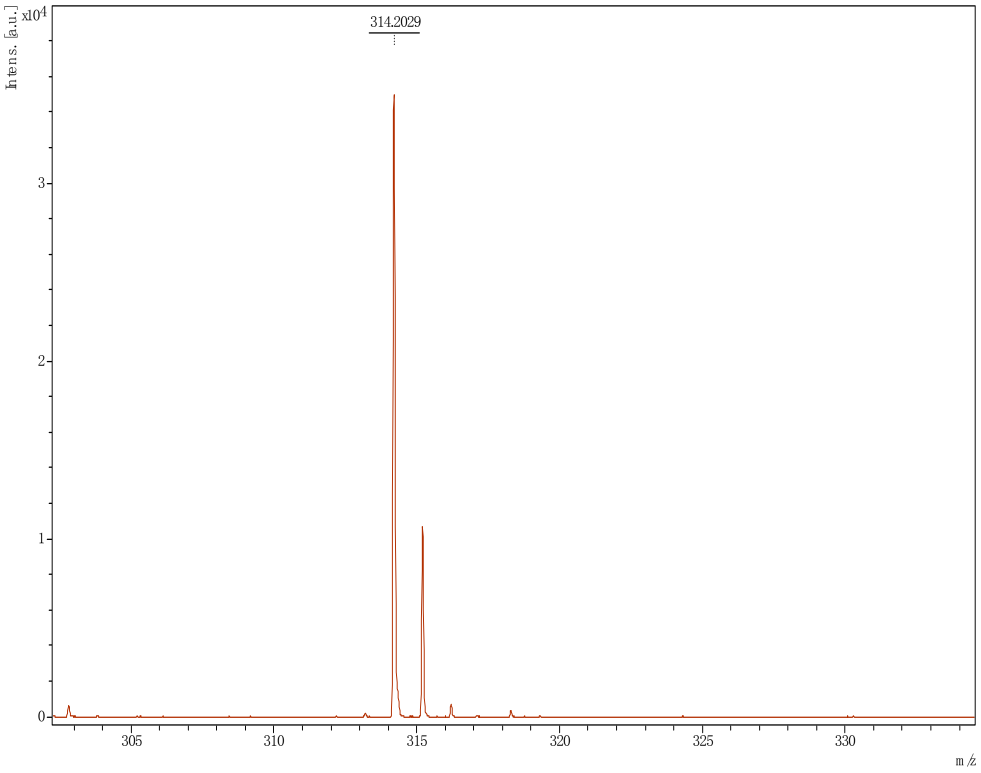

Figure S47

4-flouro-4'-(1-(p-toly)etyl)-1,1'-biphenyl (3q)

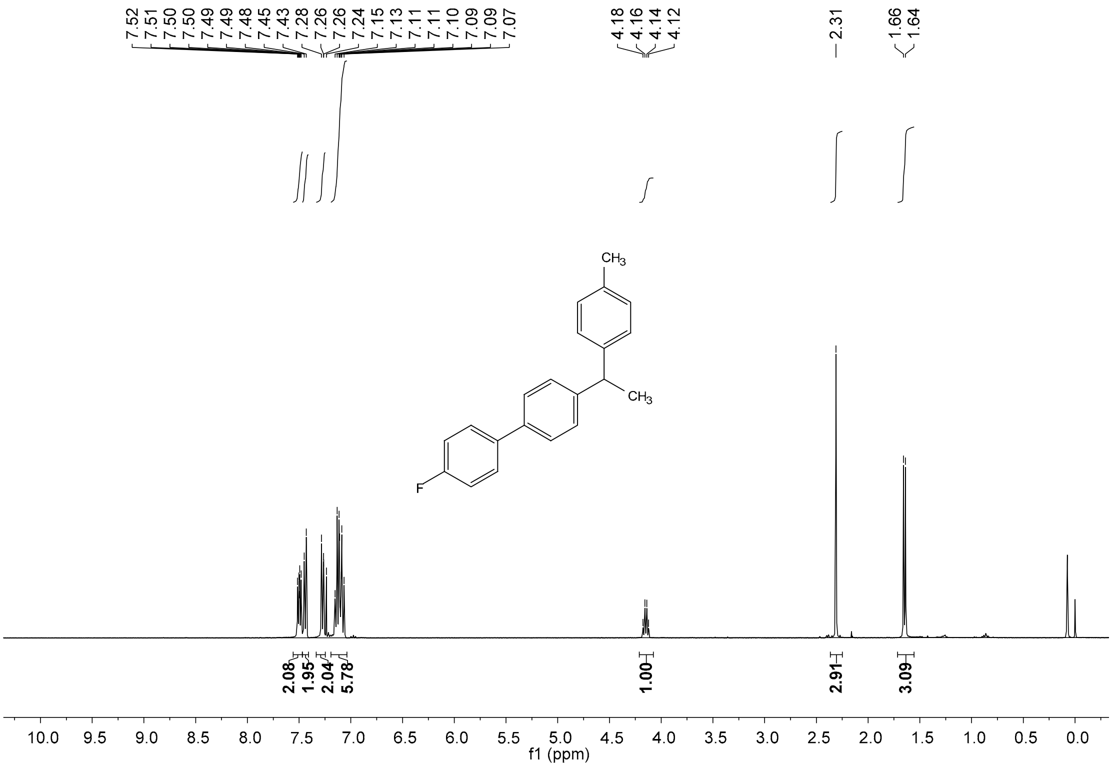

Figure S48

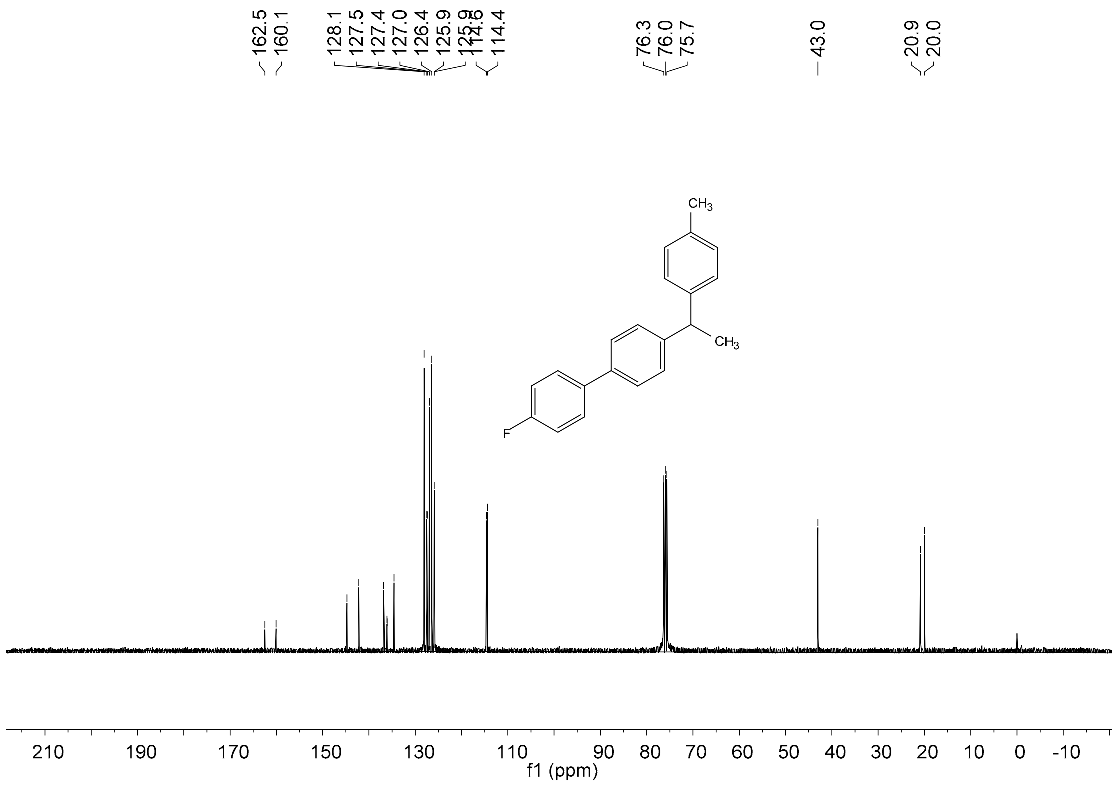

Figure S49

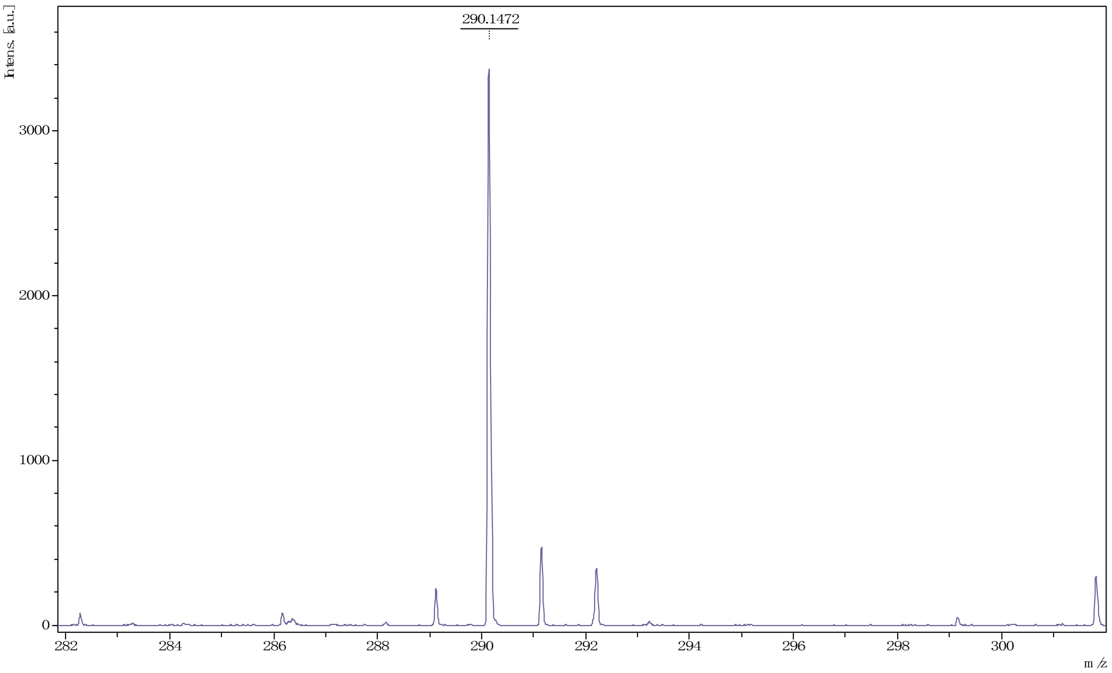

Figure S50

4-(1-(p-tolyl)ethyl)-4'-(trifluoromethyl)-1,1'-biphenyl (3r)

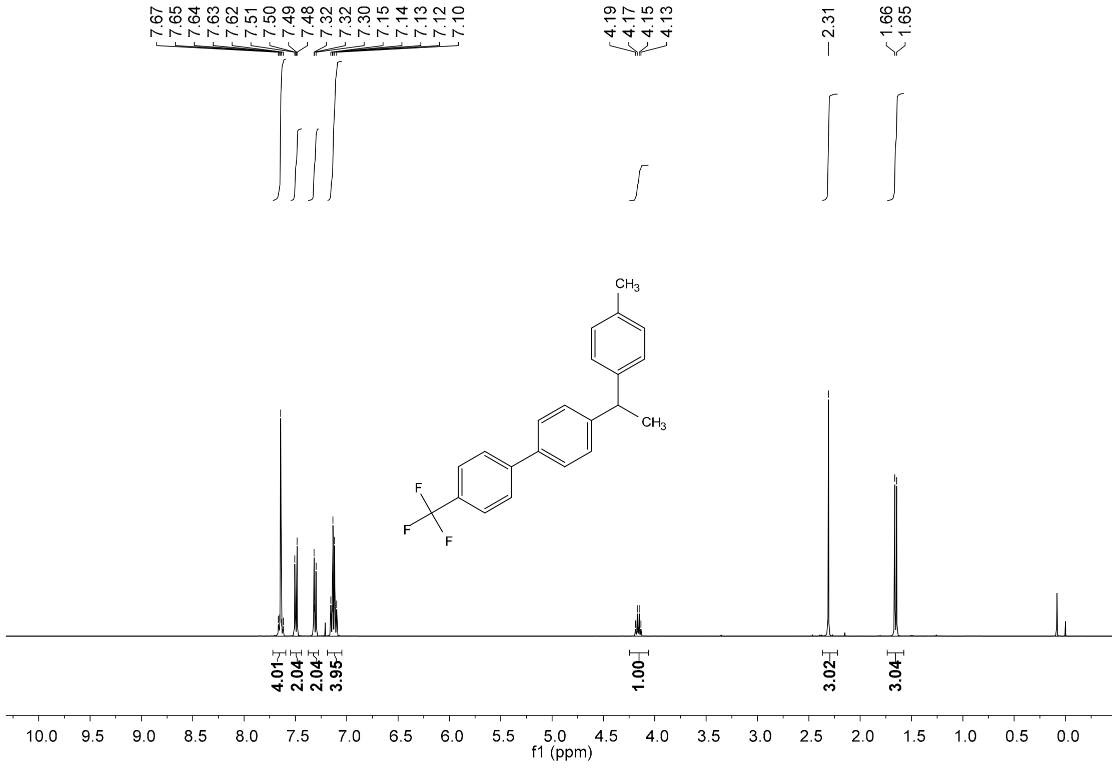

Figure S51

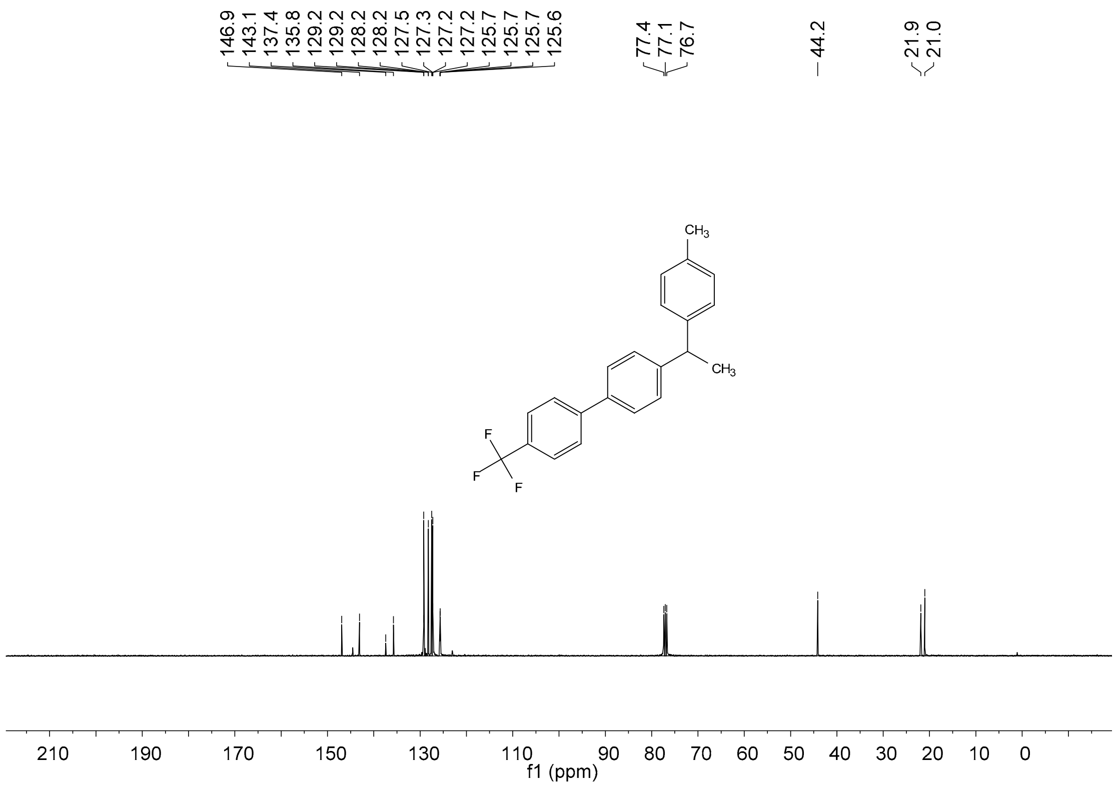

Figure S52

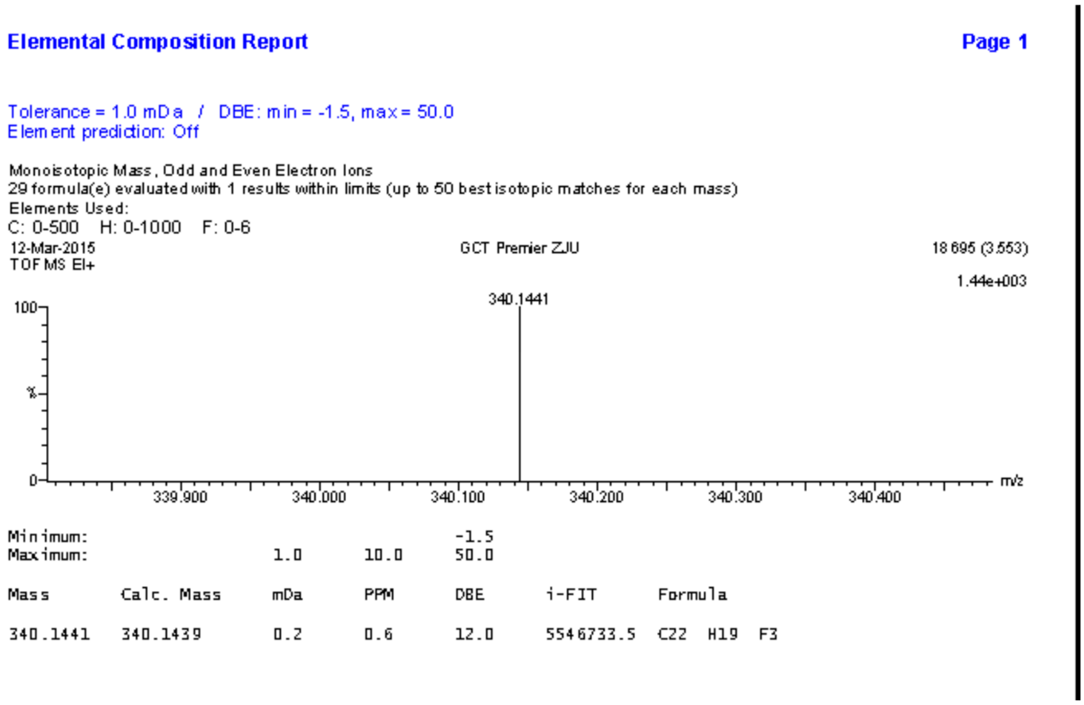

Figure S53

2,4-difluoro-4'-(1-(p-tolyl)ethyl)-1,1'-biphenyl (3s)

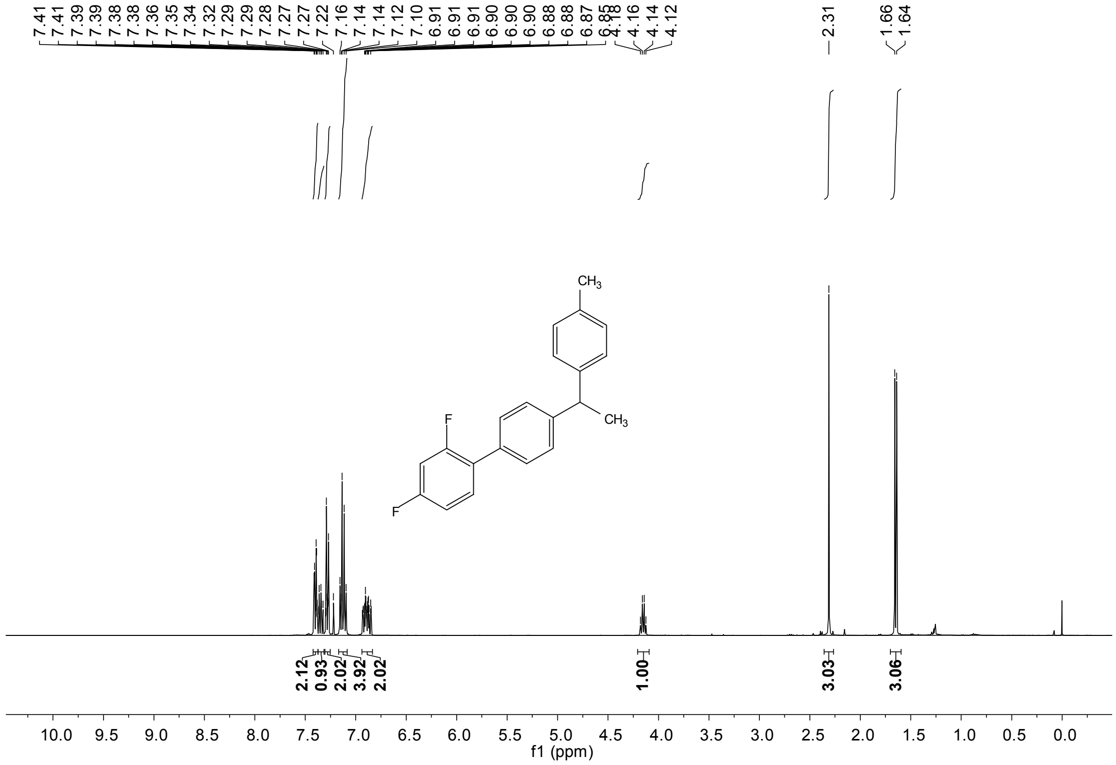

Figure S54

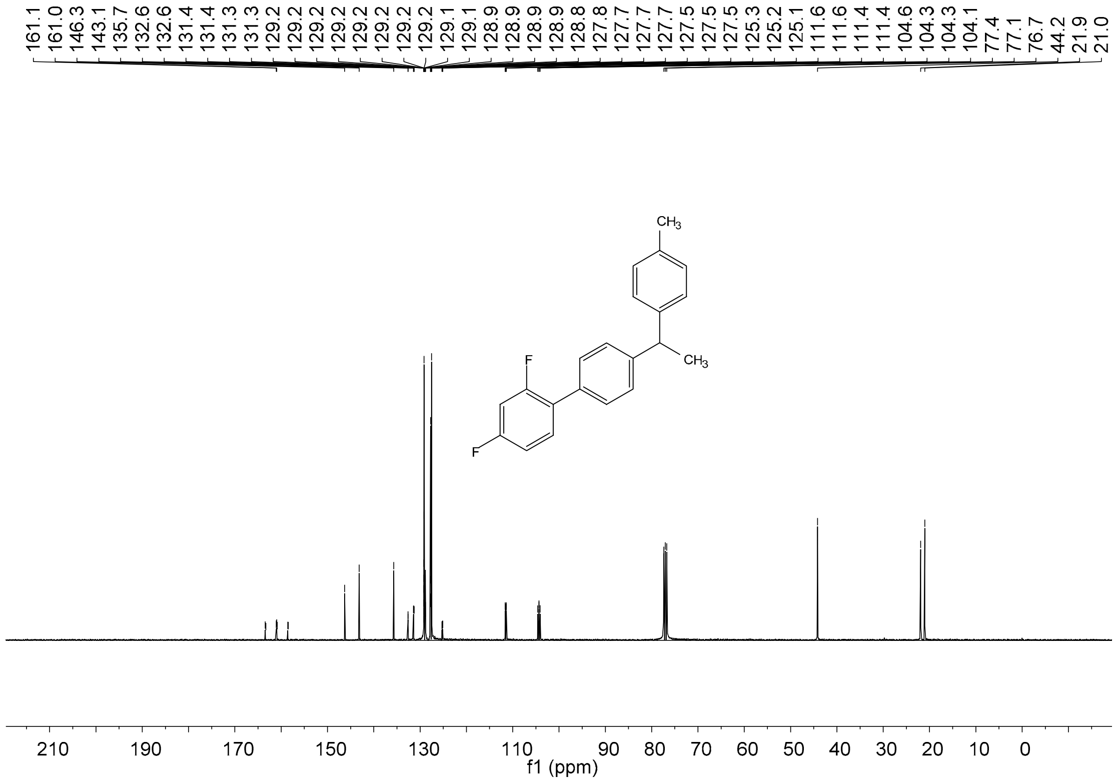
161.1
161.0
146.3
143.1
135.7
132.6
132.6
131.4
131.4
131.3
131.3
129.2
129.2
129.2
129.2
129.2
129.2
129.2
129.2
129.1
129.1
128.9
128.9
128.9
128.8
128.8
127.8
127.7
127.7
127.7
127.5
127.5
127.5
125.3
125.2
125.1
111.6
111.6
111.4
111.4
104.6
104.3
104.3
104.1
77.4
77.1
76.7
44.2
21.9
21.0
CH3
F
CH3
F
210
190
170
150
130
110
90
80
70
60
50
40
30
20
10
0
f1 (ppm)

Figure S55

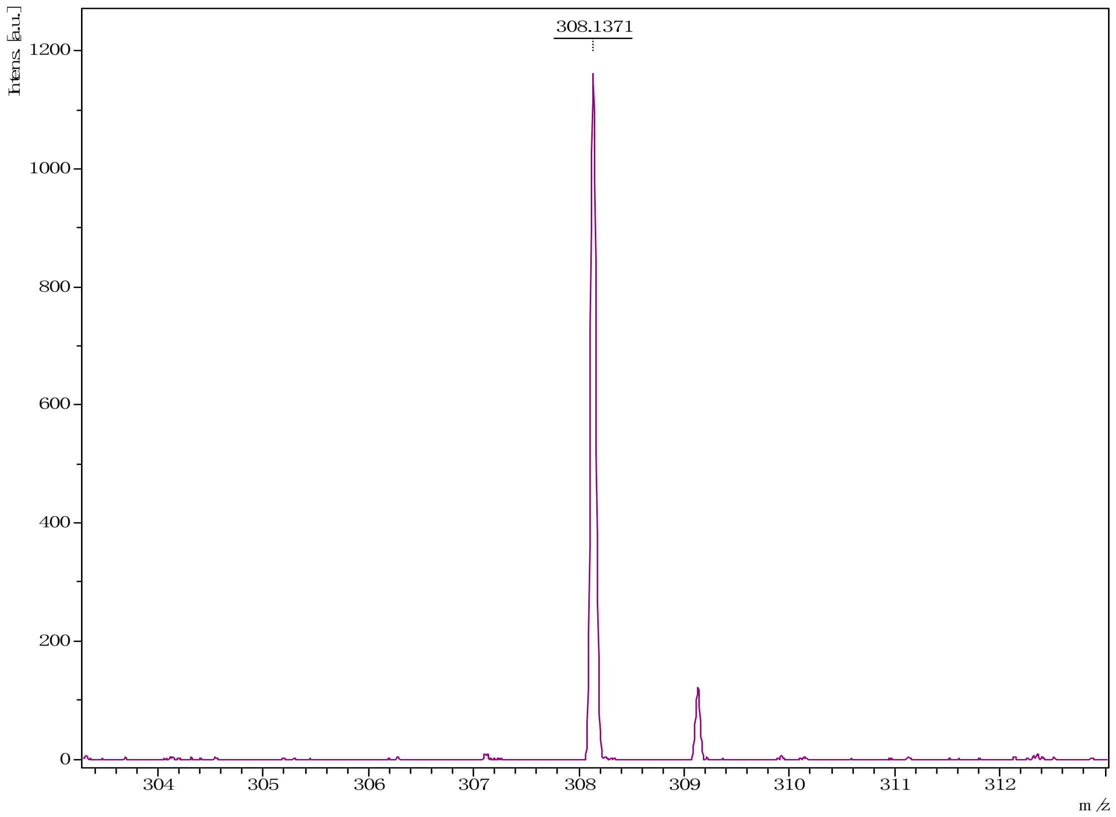
308.1371
Intens. [a.u.]
1200
1000
800
600
400
200
0
304
305
306
307
308
309
310
311
312
m/z

Figure S56

9-(4-(1-([1,1'-biphenyl]-4-yl)ethyl)phenyl)-9H-carbazole (3t)

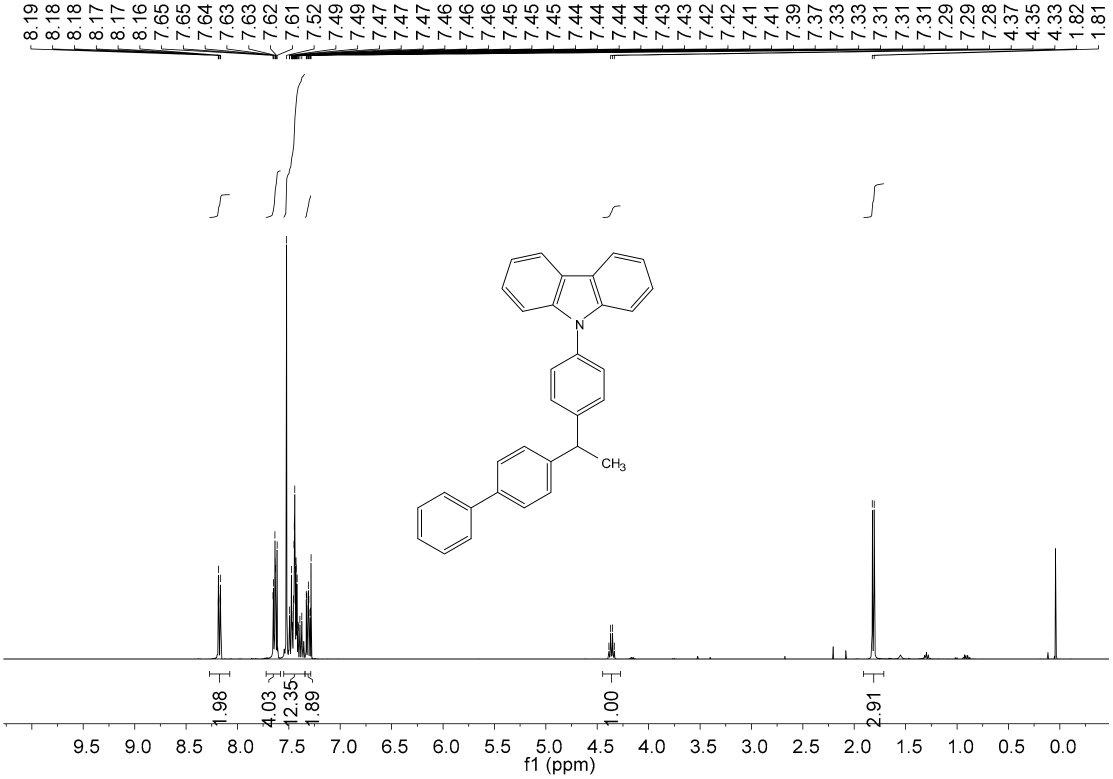

Figure S57

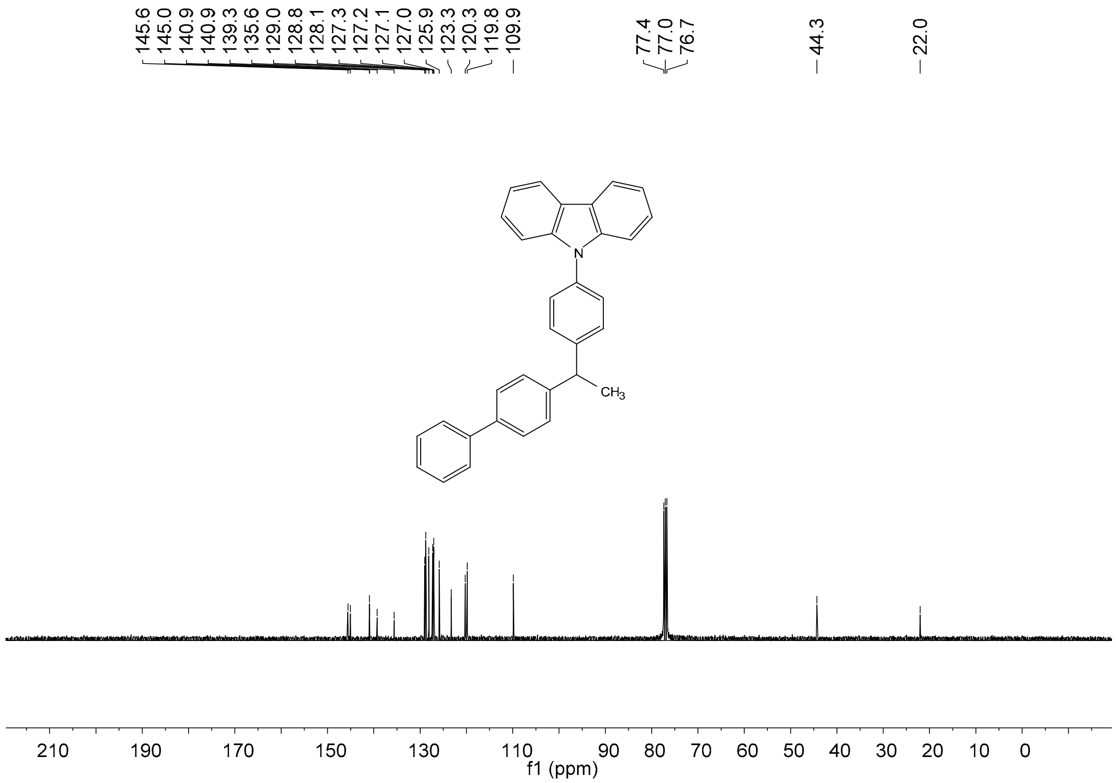

Figure S58

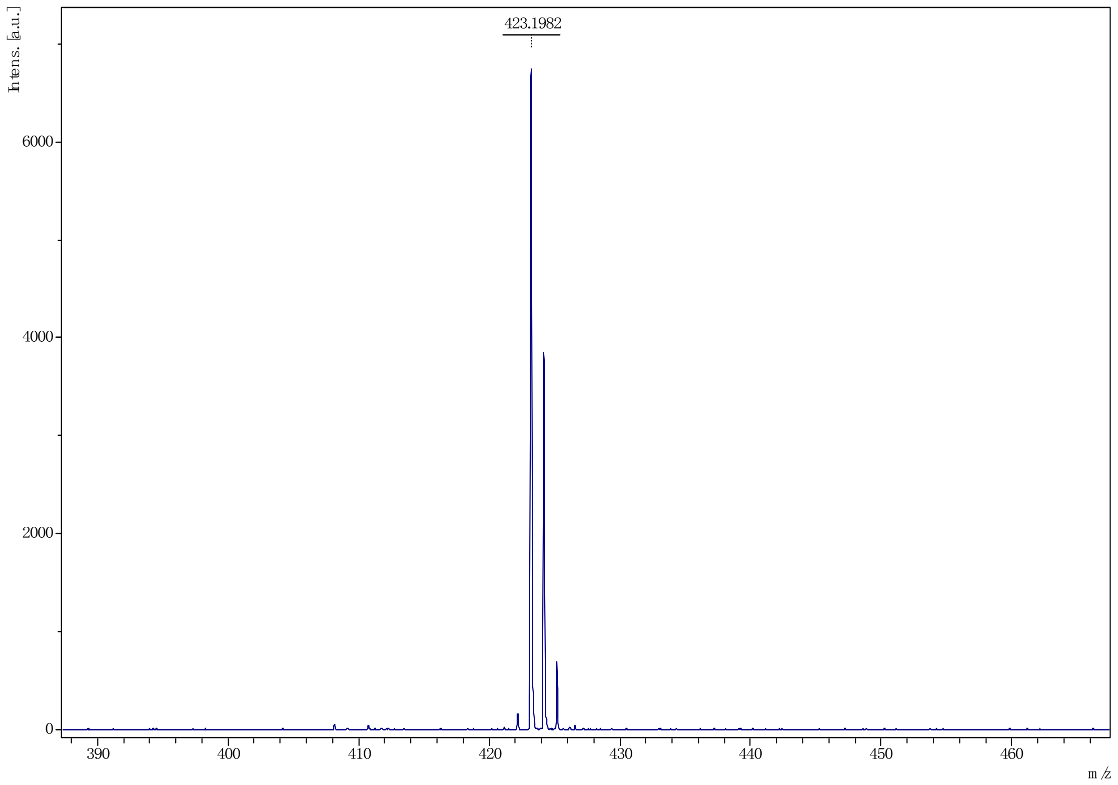

Figure S59

4'-methoxy-3-(1-(p-tolyl)ethyl)-1,1'-biphenyl (3u)

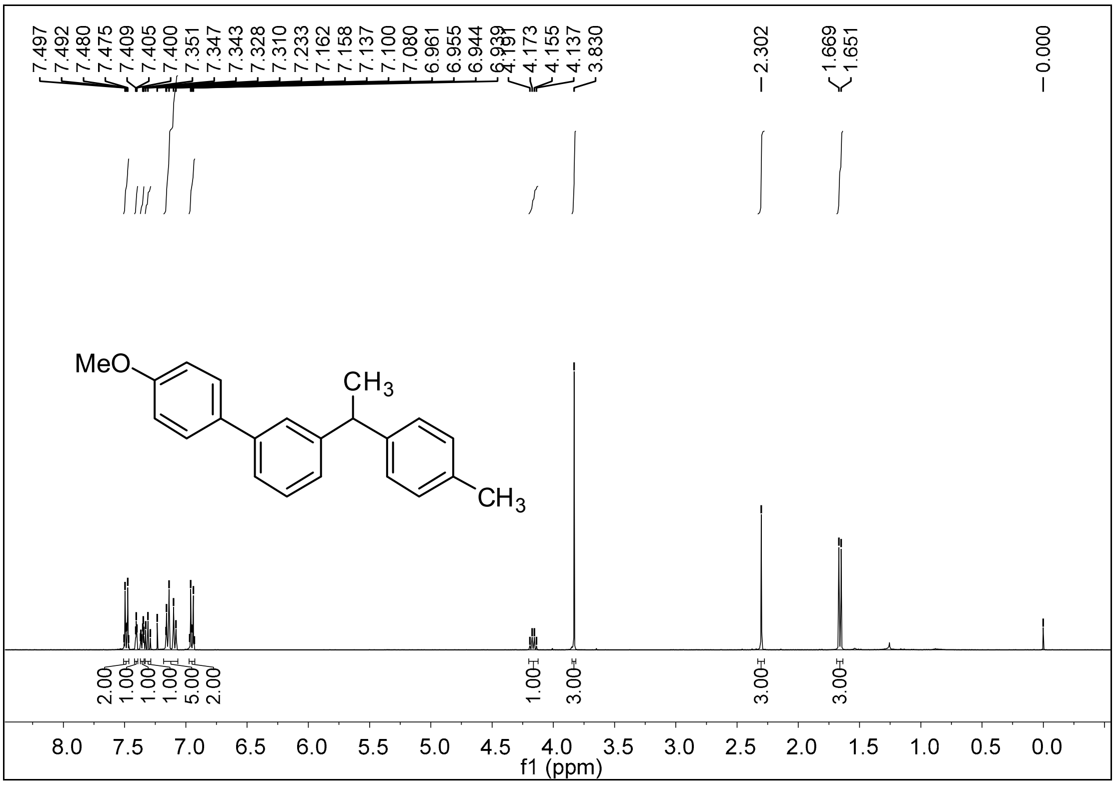

Figure S60

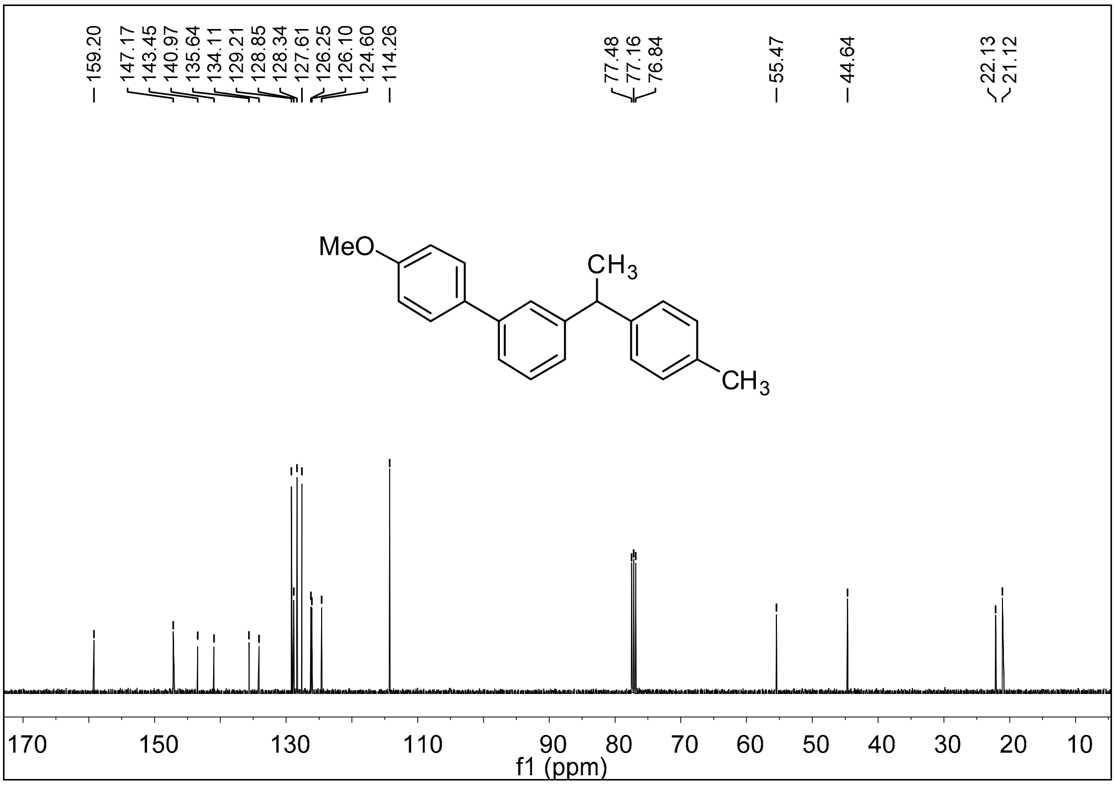

Figure S61

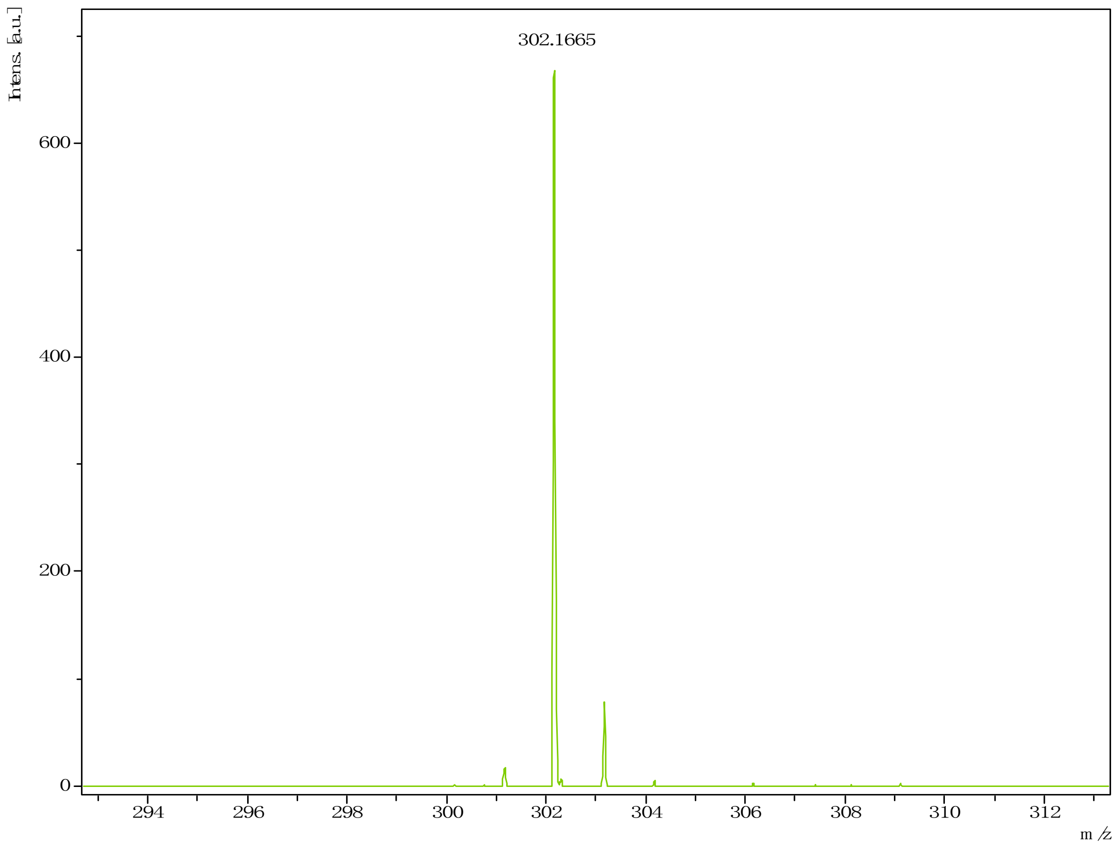

Figure S62

4-methoxy-4'-(4-methylbenzyl)-1,1'-biphenyl (3v)

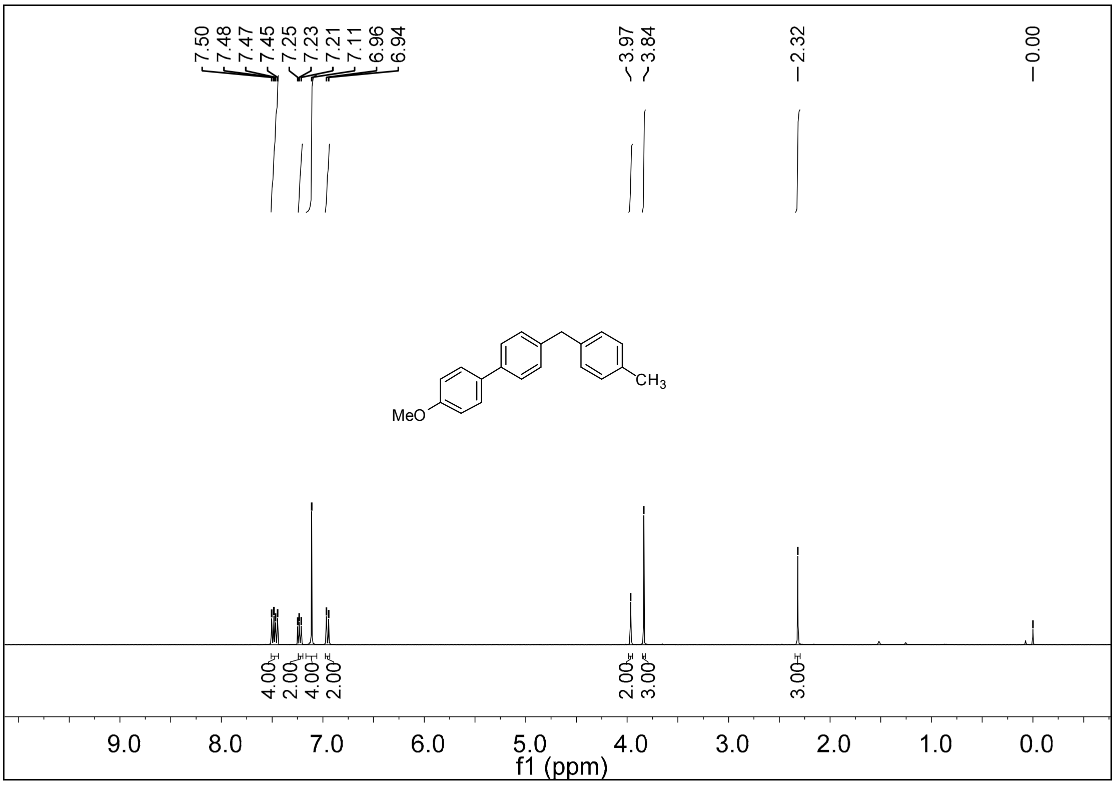

Figure S63

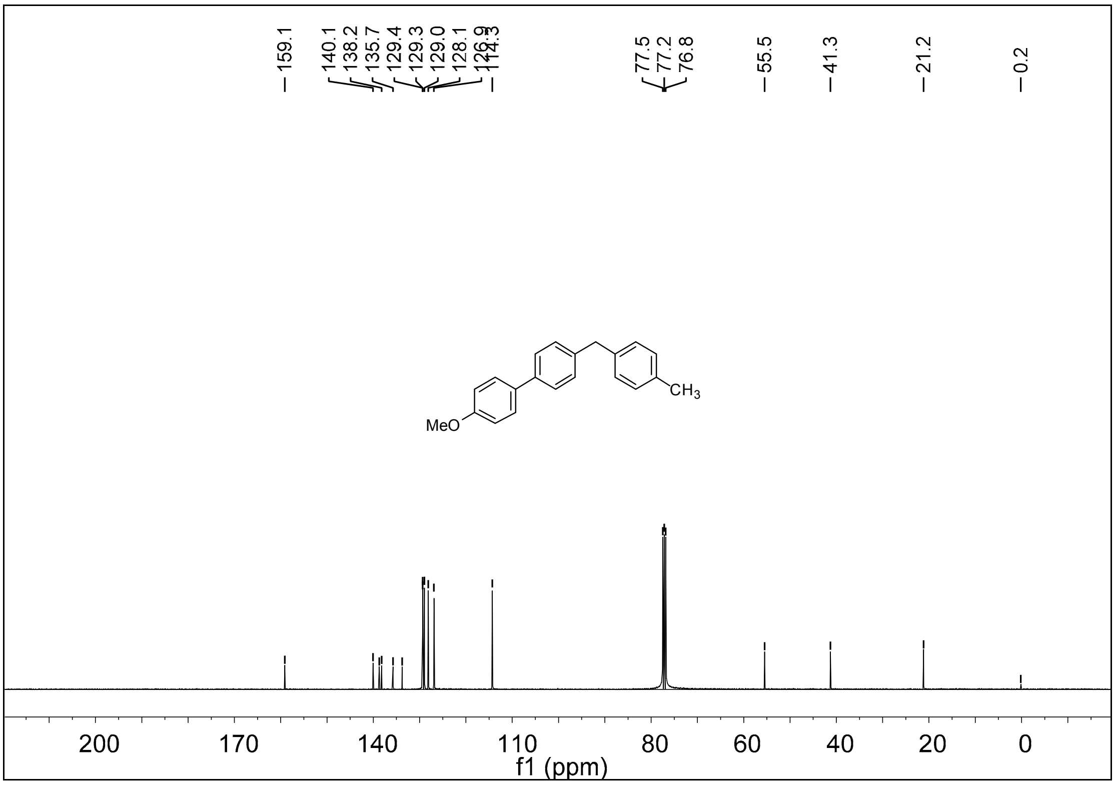

Figure S64

Elemental Composition Report **Page 1**

Tolerance = 0.7 mDa / DBE: min = -1.5, max = 50.0
Element prediction: Off

Monoisotopic Mass, Odd and Even Electron Ions
24 formula(e) evaluated with 1 results within limits (up to 70 best isotopic matches for each mass)
Elements Used:
C: 0-50 H: 0-100 O: 0-5
GCT Premier ZJU 26-Jun-2015 gn-h45 801 (3.890)
TOF MS EI+

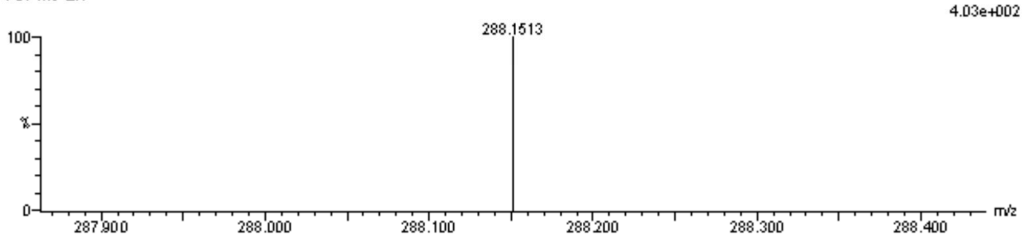

Minimum:				-1.5		
Maximum:		0.7	10.0	50.0		
Mass	Calc. Mass	mDa	PPM	DBE	i-FIT	Formula
288.1513	288.1514	-0.1	-0.3	12.0	5546214.0	C21 H20 O

Figure S65

4′-methoxy-2-(4-methylbenzyl)-1,1'-biphenyl (3w)

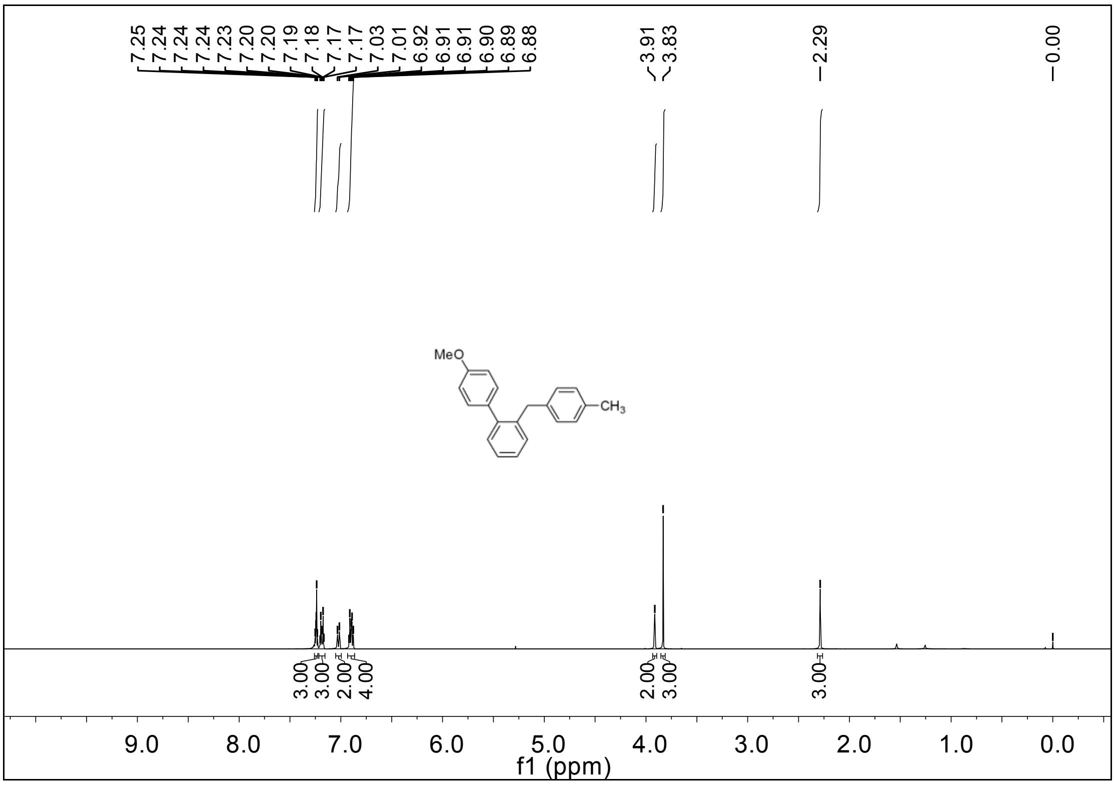

Figure S66

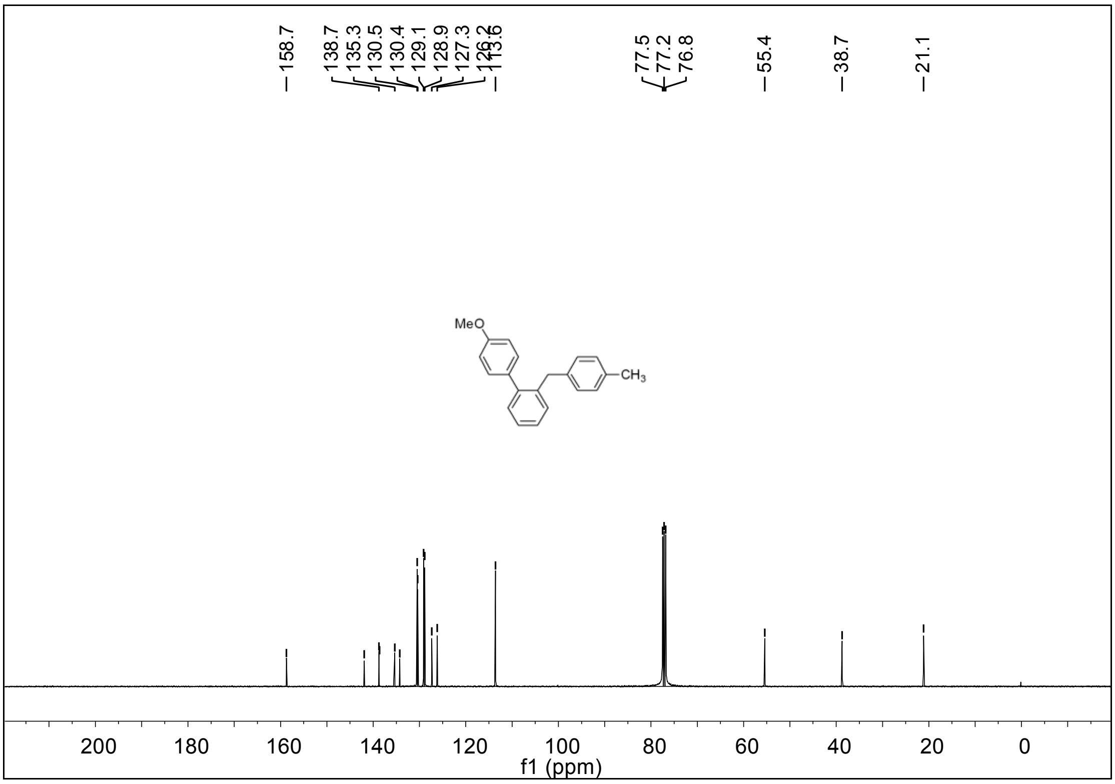

Figure S67

Elemental Composition Report **Page 1**

Tolerance = 0.7 mDa / DBE: min = -1.5, max = 50.0
Element prediction: Off

Monoisotopic Mass, Odd and Even Electron Ions
24 formula(e) evaluated with 1 results within limits (up to 70 best isotopic matches for each mass)
Elements Used:
C: 0-50 H: 0-100 O: 0-5
GCT Premier ZJU 26-Jun-2015 gn-h52 457 (2.628)
TOF MS EI+ 1.40e+003

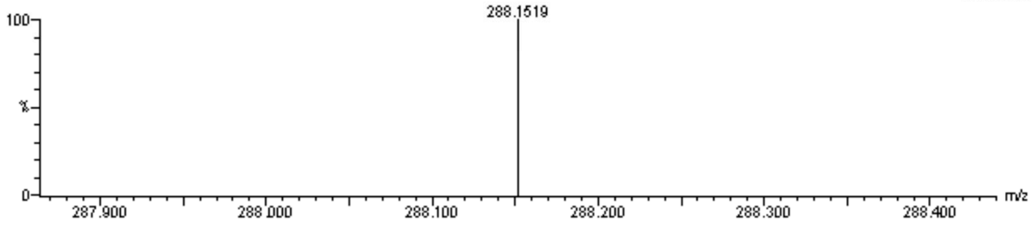

Minimum:				-1.5		
Maximum:		0.7	10.0	50.0		
Mass	Calc. Mass	mDa	PPM	DBE	i-FIT	Formula
288.1519	288.1514	0.5	1.7	12.0	5546711.5	C21 H20 0

Figure S68

4-(1-([1,1'-biphenyl]-4-yl)ethyl)pryidine (3x)

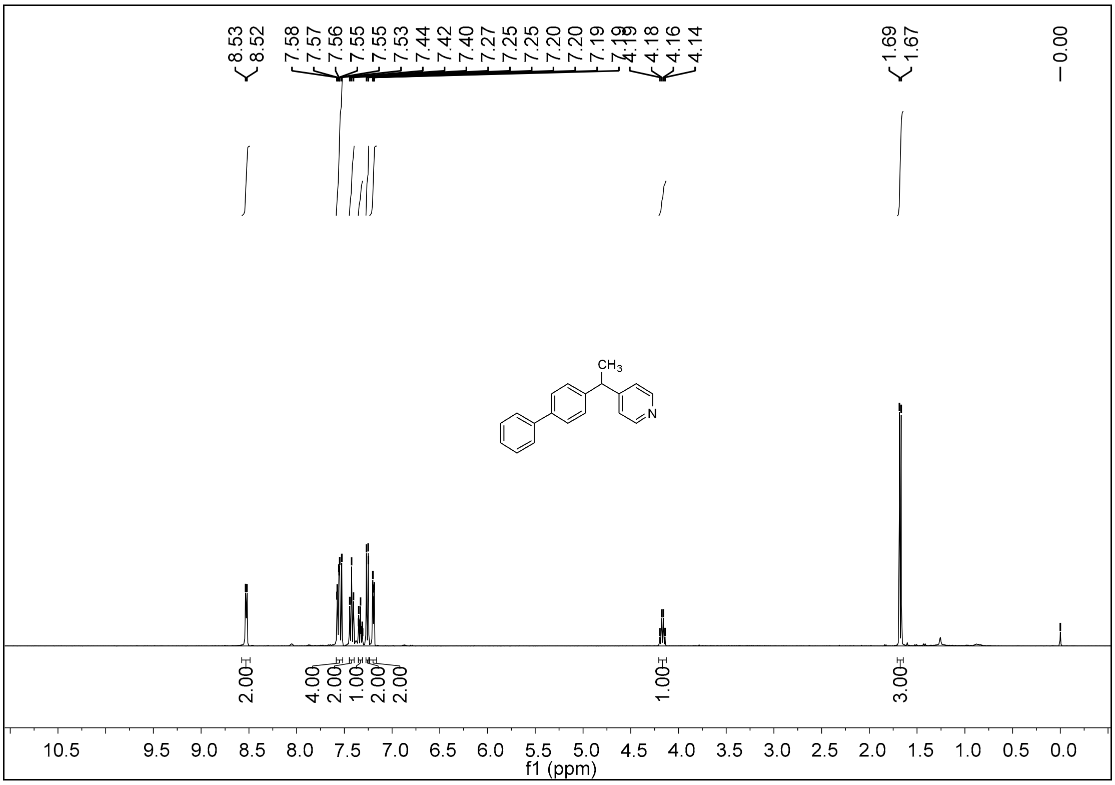

Figure S69

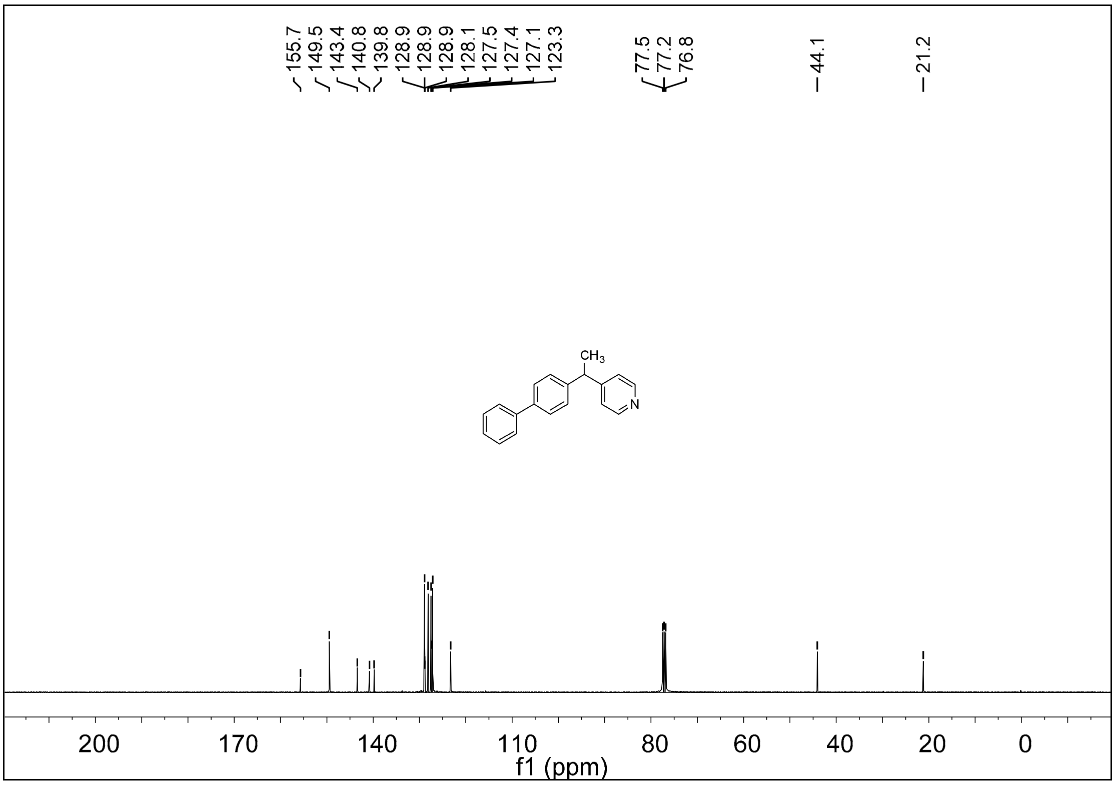

Figure S70

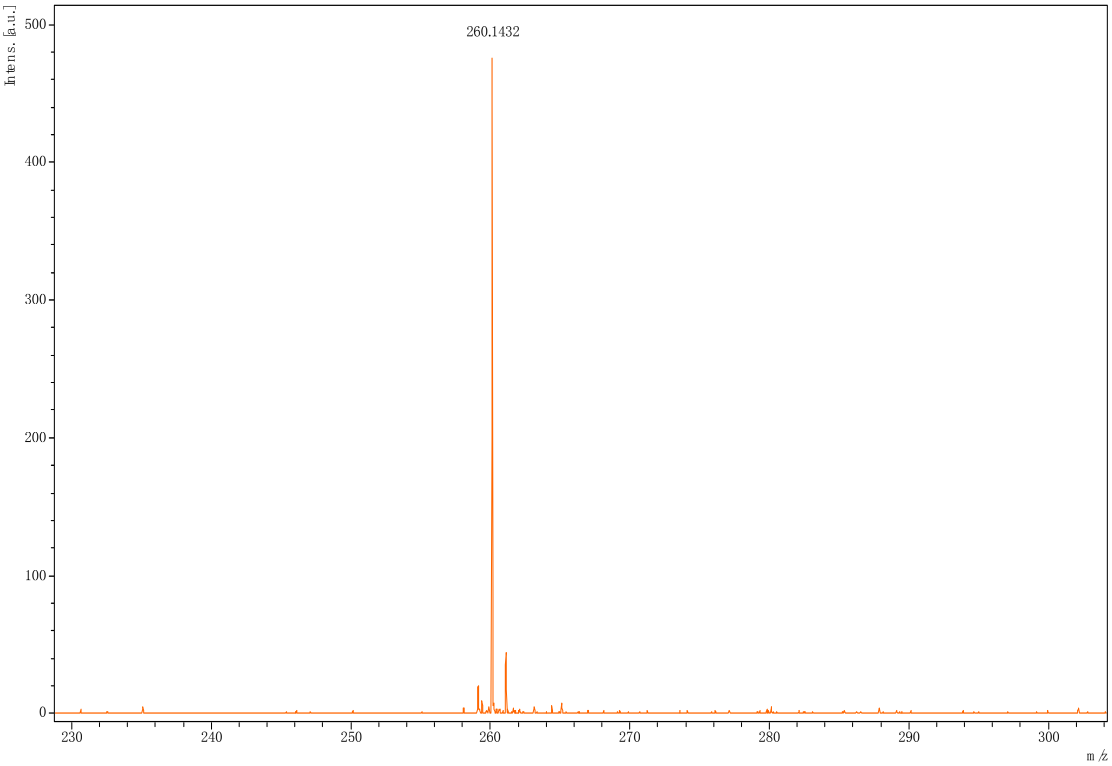

Figure S71

4-(1-(4'-methoxy-[1,1'-biphenyl]-4-yl)ethyl)pyridine (3y)

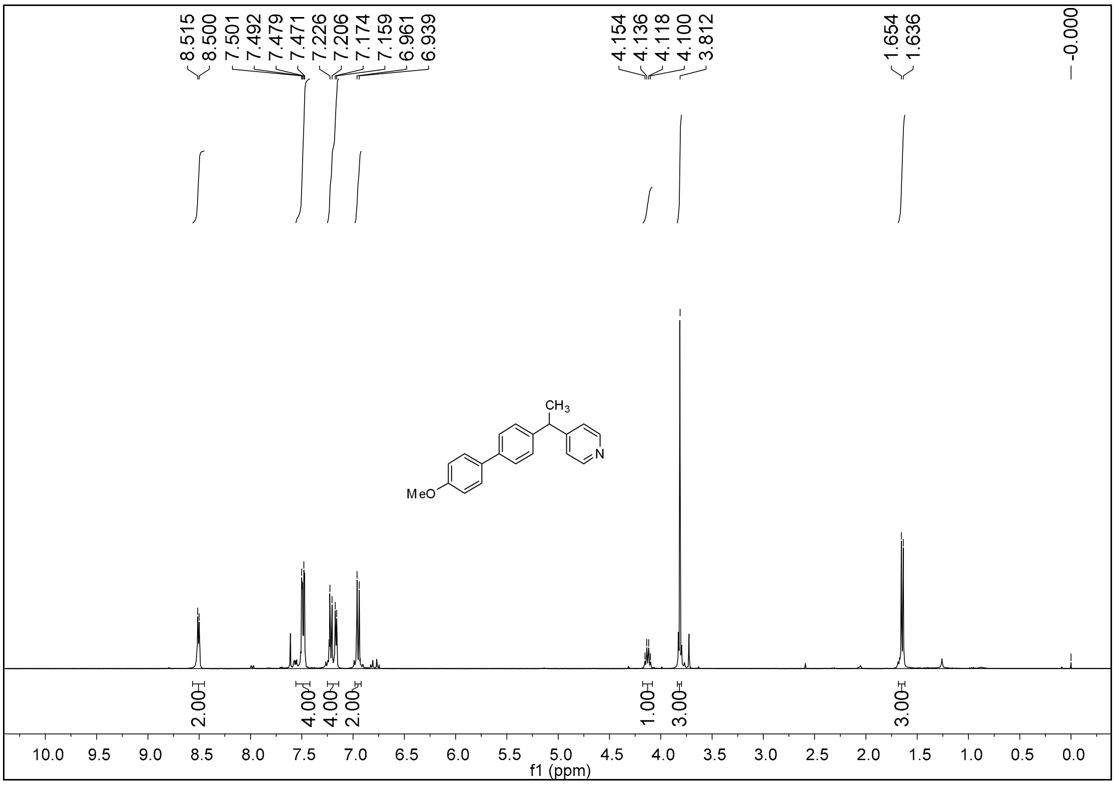

Figure S72

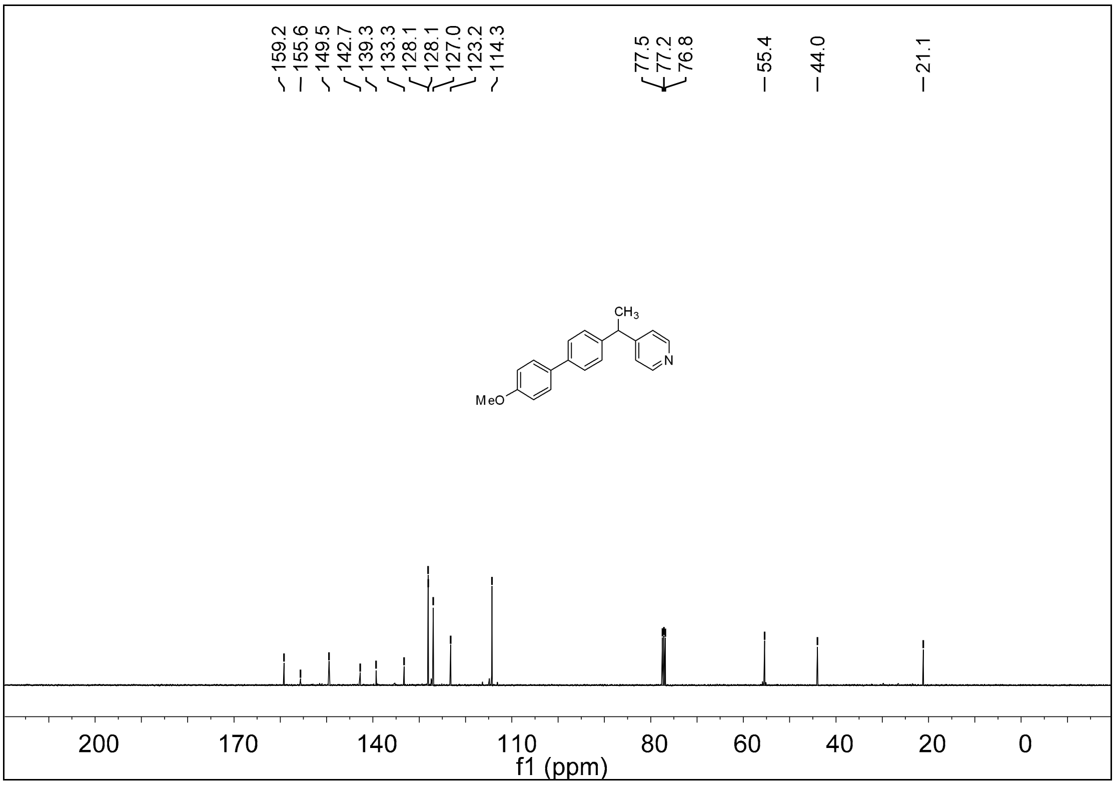

Figure S73

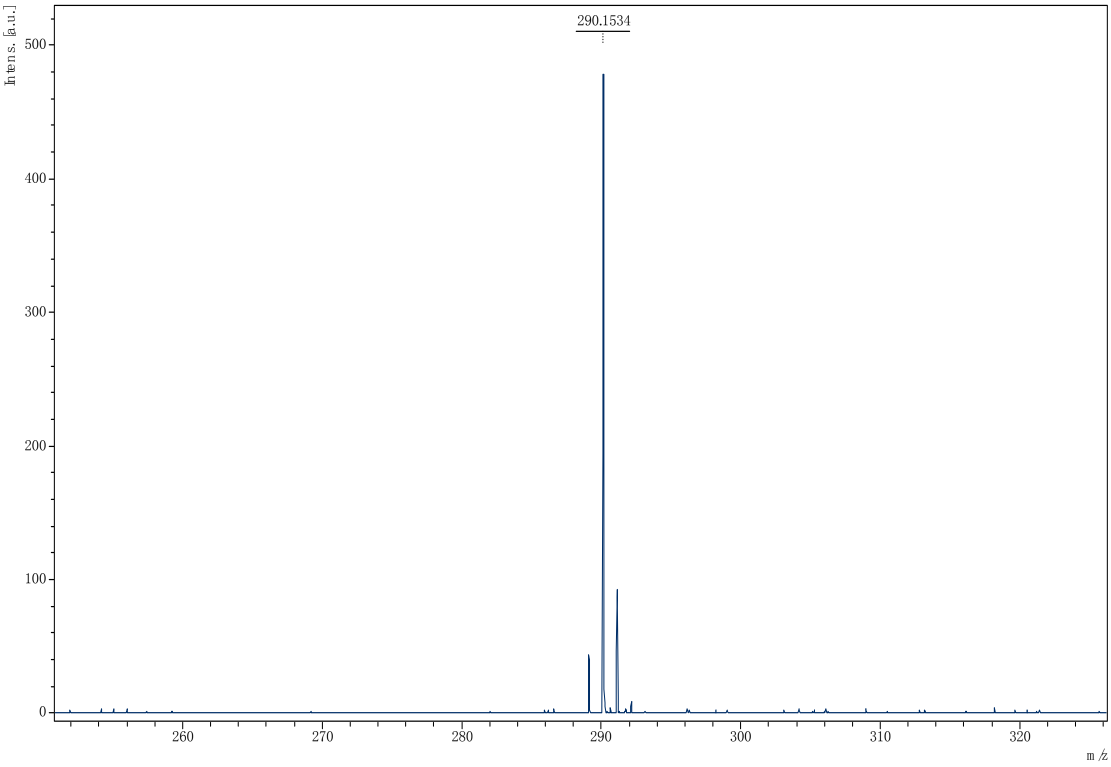

Figure S74
